# Expanding the genetic toolbox for the obligate human pathogen *Streptococcus pyogenes*

**DOI:** 10.3389/fbioe.2024.1395659

**Published:** 2024-06-07

**Authors:** Nina Lautenschläger, Katja Schmidt, Carolin Schiffer, Thomas F. Wulff, Karin Hahnke, Knut Finstermeier, Moïse Mansour, Alexander K. W. Elsholz, Emmanuelle Charpentier

**Affiliations:** ^1^ Max Planck Unit for the Science of Pathogens, Berlin, Germany; ^2^ Institut für Biologie, Humboldt-Universität zu Berlin, Berlin, Germany

**Keywords:** *Streptococcus pyogenes*, plasmid collection, inducible promoter, genetic engineering, reporter gene, scarless gene deletion, synthetic biology

## Abstract

Genetic tools form the basis for the study of molecular mechanisms. Despite many recent advances in the field of genetic engineering in bacteria, genetic toolsets remain scarce for non-model organisms, such as the obligatory human pathogen *Streptococcus pyogenes.* To overcome this limitation and enable the straightforward investigation of gene functions in *S. pyogenes*, we have developed a comprehensive genetic toolset. By adapting and combining different tools previously applied in other Gram-positive bacteria, we have created new replicative and integrative plasmids for gene expression and genetic manipulation, constitutive and inducible promoters as well as fluorescence reporters for *S. pyogenes*. The new replicative plasmids feature low- and high-copy replicons combined with different resistance cassettes and a standardized multiple cloning site for rapid cloning procedures. We designed site-specific integrative plasmids and verified their integration by nanopore sequencing. To minimize the effect of plasmid integration on bacterial physiology, we screened publicly available RNA-sequencing datasets for transcriptionally silent sites. We validated this approach by designing the integrative plasmid pSpy0K6 targeting the transcriptionally silent gene *SPy_1078*. Analysis of the activity of different constitutive promoters indicated a wide variety of strengths, with the lactococcal promoter P_*23*_ showing the strongest activity and the synthetic promoter P_*xylS2*_ showing the weakest activity. Further, we assessed the functionality of three inducible regulatory elements including a zinc- and an IPTG-inducible promoter as well as an erythromycin-inducible riboswitch that showed low-to-no background expression and high inducibility. Additionally, we demonstrated the applicability of two codon-optimized fluorescent proteins, mNeongreen and mKate2, as reporters in *S. pyogenes*. We therefore adapted the chemically defined medium called RPMI4Spy that showed reduced autofluorescence and enabled efficient signal detection in plate reader assays and fluorescence microscopy. Finally, we developed a plasmid-based system for genome engineering in *S. pyogenes* featuring the counterselection marker pheS*, which enabled the scarless deletion of the *sagB* gene. This new toolbox simplifies previously laborious genetic manipulation procedures and lays the foundation for new methodologies to study gene functions in *S. pyogenes,* leading to a better understanding of its virulence mechanisms and physiology.

## 1 Introduction

Studying the function of a gene of interest requires appropriate tools for modifying, deleting or inserting genetic information. To do so, plasmid-based systems have been developed that aim to facilitate point mutations, scarless deletions or allelic replacements (Ellinger, 2016). Various native or synthetic promoters can be used to modify the expression levels of a gene of interest to identify potential toxic or dose-dependent effects (Sorg et al., 2015; Popp et al., 2017; Schuster and Reisch, 2021). In addition, different enzyme-based and fluorescent reporters enable the investigation of gene expression profiles, protein localization, protein-protein interactions and the tracking of bacteria during infection or microbial community studies (Lagendijk et al., 2010; Vega et al., 2013; Lamb et al., 2018).

Genetic toolboxes have mainly been designed for highly-studied model organisms, such as *Bacillus subtilis*, or organisms useful for biotechnological purposes like Cyanobacteria (Ellinger, 2016). However, for many pathogenic bacteria, the genetic toolkit remains limited, despite their clinical relevance. One example is the obligatory human pathogen *Streptococcus pyogenes*, also known as Group A *Streptococcus* (GAS). Worldwide, GAS is responsible for over 700 million infections and around half a million deaths annually (Ferretti et al., 2001; Nguyen and McShan, 2014). Although there is extensive knowledge on many of the encoded virulence factors, a large percentage of the ∼1,800 genes remains to be studied (Cho et al., 2019). A better understanding of the physiological role of these genes could lead to the identification of potential new targets for drug and vaccine development.

Despite the availability of a number of plasmid systems for protein expression or genetic engineering in GAS, most current systems were developed decades ago and, since then, have rarely been optimized or expanded (Cho et al., 2019). To edit the genome of *S. pyogenes*, researchers have previously used methodologies based on single- and double-crossover homologous recombination (HR), facilitated by conditional plasmids with thermosensitive origins or the Cre-*lox* system, respectively (Sternberg and Hamilton, 1981; Sauer and Henderson, 1988; Cho et al., 2019). However, plasmid curing makes these approaches rather laborious. A faster approach uses so-called “suicide” plasmids that are unable to replicate in the target strains and insert into specific genomic sites via allelic replacement based on double-crossover HR, also introducing an antibiotic marker for selection (Radeck et al., 2013; Keller et al., 2019). To date, the only target-specific integrative plasmid described for *S. pyogenes* is pFW11e, which inserts into the *SPy_0535* locus (*S. pyogenes* strain SF370) that encodes a sugar-phosphate isomerase (Park et al., 2003).

Integrases derived from bacteriophages have also been used for site-specific insertion of DNA fragments into the bacterial genome. One example is the plasmid p7INT, which encodes the integrase and respective *attP* site from the *S. pyogenes* temperate phage T12 (McShan et al., 1998). This integrase recognizes the *attB* site located at the 3’ end of the transfer-messenger RNA (tmRNA) gene and performs a precise insertion without impairing the functionality (McShan et al., 1998). The application of this plasmid is limited to only a few *S. pyogenes* strains (e.g., NZ131, SF370, and HSC5) possessing a matching *attB* site, although a single-base mutation in the *attP* site has been shown to expand the host range (Cho et al., 2019).

For the establishment of more advanced genetic tools, such as a CRISPRi system enabling the study of essential genes (Liu et al., 2017), suitable genomic integration sites and inducible promoters are required. As a large part of the streptococcal genome remains uncharacterized, it is challenging to select different integration sites without disrupting important genes and regulatory sequences.

Furthermore, only two inducible promoters are commonly used in GAS, both of which have certain drawbacks. One such system is the nisin-controlled gene expression (NICE) system, derived from a cluster of genes involved in nisin production and conferring immunity against it in *Lactococcus lactis* (de Ruyter et al., 1996; Kuipers et al., 1998). Although the NICE system is functional in *S. pyogenes*, the sublethal concentrations of nisin required do not result in strong induction and background expression is high (Eichenbaum et al., 1998). The most frequently used inducible expression system is based on the P*tet* promoter, driving transcription of a gene of interest in the presence of (anhydro-)tetracycline. A version of P*tet* containing three operator sites (P*tet*(O)_3_) has been constructed for inducible gene expression in GAS, but has shown limitations in its dynamic range (Bugrysheva et al., 2011). Hence, it is first necessary to develop new, optimized systems for tunable gene expression and to determine suitable integration sites, before more advanced genetic engineering approaches can be implemented.

Reporter genes are essential for studying the expression of a gene or the sub-cellular localization of a protein of interest. The panel of reporter genes available for GAS is small, with a primary focus on the firefly luciferase (*ffluc*) or the β-glucuronidase (*gusA*) for assessing promoter activities (Biswas and Scott, 2003; Park et al., 2003; Loh and Proft, 2013; Lamb et al., 2018). Only few studies report the use of fluorescent proteins (FPs), such as GFP or mCherry, in *S. pyogenes* (Nerlich et al., 2009; Aymanns et al., 2011; Vega et al., 2013; Liang et al., 2019). Previous studies have argued that streptococcal growth conditions, such as low oxygen supply and acidification of the growth medium by lactic acid production negatively impact GFP function (Hansen et al., 2001; Aymanns et al., 2011). However, new generations of FPs with improved properties, e.g., higher stability at low pH, increased brightness and accelerated folding times, remain to be analyzed in GAS (Merzlyak et al., 2007; Hostettler et al., 2017).

In this study, we compiled a comprehensive genetic toolbox for *S. pyogenes* to enable the implementation of more advanced genetic engineering technologies in GAS. We developed a set of replicative and integrative plasmids that enable sub-cloning procedures in *Escherichia coli* and the expression of proteins of interest in *S. pyogenes*. We compared the activity of various constitutive promoters to determine the variability in promoter strengths and characterized novel inducible regulatory elements allowing tunable gene expression. Furthermore, two codon-optimized fluorescent reporters, mNeongreen and mKate2, were tested for their functionality in *S. pyogenes*. For this, a chemically defined medium has been adapted and evaluated that enables fluorescence measurements in plate reader and microscopy setups. Finally, we designed and tested a new plasmid system featuring the counterselection marker pheS* for the implementation of scarless gene deletions in *S. pyogenes*.

## 2 Materials and methods

### 2.1 Bacterial strains and growth conditions

*E. coli* DH5α, One Shot™ TOP10 or BW25113 (for reporter plasmids containing P_*lac*(*Spn*)_ and derivatives) were grown in Luria-Bertani (LB medium) at 37°C with aeration (180 r.p.m.) or on LB agar plates at 37°C (Supplementary Table S1). Carbenicillin (100 μg/mL), chloramphenicol (20 μg/mL), kanamycin (50 μg/mL) or erythromycin (300 μg/mL) were used for selection. Transformation of chemically competent *E. coli* was performed using a heat shock-based protocol as described previously (Chang et al., 2017). *S. pyogenes* SF370 (serotype M1) and derivatives (Supplementary Table S1) were grown in THY medium (30 g/L Todd-Hewitt Broth (Bacto, Becton Dickinson) with 2 g/L Yeast extract (Servabacter)), in GAS chemically defined medium [GAS CDM Powder from Alpha BioSciences supplemented with 1% w/v glucose, ferric nitrate nonahydrate (50 mg/L), ferrous sulfate heptahydrate (5 mg/L), manganese sulfate monohydrate (3.4 mg/L), NaHCO_3_ (2.5 g/L) and L-cysteine (708 mg/L)] (Gera and McIver, 2013), or on Trypticase Soy Agar (TSA) plates containing 5% sheep blood or THY agar plates at 37°C and 5% atmospheric CO_2_. For fluorescence measurements, *S. pyogenes* was cultured in RPMI4Spy medium [Gibco™ RPMI1640 medium with glutamine and without phenol red, 1x Nucleobase mix (20 mg/L Guanine, 20 mg/L Adenine and 20 mg/L Uracil), 1x BME Vitamin solution, 1x RPMI Amino Acids solution, 7 g/L Glucose, 0.02 mM HEPES buffer (pH 7.4), 10 mg/L Niacinamide]. *S. pyogenes* electrocompetent cells were prepared and transformed as previously described using BioRad 1 mm cuvettes and electroporation at 400 Ω, 25 μF and 1800 V (Caparon et al., 1991; Le Rhun et al., 2017). Chloramphenicol (20 μg/mL and 5 μg/mL for plasmids from pSpy2 series), kanamycin (300 μg/mL) or erythromycin (3 μg/mL) were added for selection when necessary.

### 2.2 Growth curves

*S. pyogenes* SF370 wildtype and derivatives were streaked on TSA blood agar plates with selective antibiotics if required. Alternatively, replicative plasmids were freshly transformed into *S. pyogenes* SF370 sucrose-competent cells. Strains or transformants were grown at 37°C and 5% CO_2_ overnight. The next day, overnight cultures were inoculated from single colonies in THY medium and incubated at 37°C with 5% CO_2_ overnight. Overnight cultures were harvested by centrifugation at 4,500 × g for 10 min and adjusted to an OD_620nm_ of 0.02 in THY medium. For maintenance of the replicative plasmids, corresponding selective antibiotics were supplemented at all times, while no antibiotics were applied for strains harboring integrative plasmids. To assess the effect of different chloramphenicol concentrations on the growth of the wildtype or *S. pyogenes* harboring pSpy1C, pSpy2C or pSpy3C, we supplemented different chloramphenicol concentrations (1 μg/mL, 2.5 μg/mL, 5 μg/mL, 10 mg/mL and 20 μg/mL) and 0.1% ethanol as control. Then, 200 µL of each sample were applied to a clear 96-well plate in technical triplicates together with the blank (medium only). Growth was measured every 20 min for 15 h in a plate reader (Synergy H1, BioTek) at 37°C and 5% CO_2_. Experiments were performed in biological triplicates. Statistical differences between generation times (G = ln (2)/µ where µ is the growth rate) were calculated using a two-sample t-test [n.s. = not significant, *p* < 0.05 = significant (*)].

### 2.3 Extraction of genomic DNA

Genomic DNA of *S. pyogenes* and *B. subtilis* PY79 was prepared using the NucleoSpin Microbial DNA Kit (Macherey Nagel) following the manufacturer’s protocol. The lysis step was performed using the FastPrep-24 5G from MP Biomedicals (*S. pyogenes* program 20 s, 2x).

### 2.4 Plasmid construction

All PCR reactions were conducted using either Q5 polymerase or Phusion™ High Fidelity Polymerase (Thermo Scientific) except for colony PCRs (Bergkessel et al., 2013), which were performed using OneTaq^®^ Polymerase (NEB). For plasmid assembly, we followed either the Golden Gate Assembly or Gibson Assembly^®^ strategy (Engler et al., 2008; Gibson et al., 2009). After transformation into *E. coli*, plasmid sequences were validated using Sanger sequencing (Microsynth Seqlab GmbH). Oligos, plasmids and DNA sequences of the most important genes and promoters from this study can be found in Supplementary Tables S2–S4, respectively. Newly constructed backbones and plasmids containing relevant inserts used in this study will be made available through Addgene.

#### 2.4.1 Replicative plasmid backbones

All replicative plasmid backbones were constructed using the Golden Gate Assembly strategy with the type II restriction enzyme *Esp*3I (NEB) according to the manufacturer’s protocol (Table 1). Plasmid backbones were designed based on the shuttle plasmids pIB166, pIB184-Km and pIB185 as well as pBAV1K-T5-*gfp* (Biswas et al., 2008; Bryksin and Matsumura, 2010; Hossain and Biswas, 2012). Plasmids pIB166 (Addgene plasmid # 90189), pIB184-Km (Addgene plasmid # 90195) and pIB185 (Addgene plasmid # 90196) were donated by Indranil Biswas. pBAV1K-T5-gfp was a gift from Ichiro Matsumura (Addgene plasmid # 26702).

**TABLE 1:** Replicative and integrative plasmid backbones developed in this study.

Plasmid	Type	Size in bp	PCN	Features
pSpy1C	replicative	4,917	high	pSH71 replicon, P_*lac*(*Eco*)__*mrfp* in MCS, Cm^R^
pSpy1K	replicative	4,693	high	pSH71 replicon, P_*lac*(*Eco*)__*mrfp* in MCS, Kan^R^
pSpy1E	replicative	4,801	high	pSH71 replicon, P_*lac*(*Eco*)__*mrfp* in MCS, Erm^R^
pSpy2C	replicative	6,869	low	pAMβ1 replicon, pUC origin, P_*lac*(*Eco*)__*mrfp* in MCS, Cm^R^
pSpy2E	replicative	6,644	low	pAMβ1 replicon, pUC origin, P_*lac*(*Eco*)__*mrfp* in MCS, Erm^R^
pSpy3C	replicative	4,047	high	pWV01* replicon, P_*lac*(*Eco*)__*mrfp* in MCS, Cm^R^
pSpy3K	replicative	3,741	high	pWV01* replicon, P_*lac*(*Eco*)__*mrfp* in MCS, Kan^R^
pSpy3E	replicative	3,978	high	pWV01* replicon, P_*lac*(*Eco*)__*mrfp* in MCS, Erm^R^
pSpy0C2	integrative	5,618	n.a.	pUC origin, P_*lac*(*Eco*)__*mrfp* in MCS, homology arms for integration into *amyA*, Cm^R^
pSpy0C4	integrative	5,256	n.a.	pUC origin, P_*lac*(*Eco*)__*mrfp* in MCS, homology arms for integration into *sagB*, Cm^R^
pSpy0K6	integrative	5,344	n.a.	pUC origin, P_*lac*(*Eco*)__*mrfp* in MCS, homology arms for integration into *SPy_1078*, Kan^R^
p7INT.1	integrative	5,035	n.a.	pUC origin, P_*lac*(*Eco*)__*mrfp* in MCS, T12 integrase and *attP* site, Erm^R^
pERASE	integrative	4,457	n.a.	pUC origin, P_*lac*(*Eco*)__*mrfp* in MCS, pheS* counterselection marker under control of P_*veg*_, Erm^R^

MCS, multiple cloning site; Cm^R^, chloramphenicol resistance; Kan^R^, kanamycin resistance; Erm^R^, erythromycin resistance; PCN, plasmid copy number (qualitative); n.a., not applicable.

All *Esp*3I recognition sites present in the backbones were removed by PCR mutagenesis using primer pairs OLEC9801/9802, OLEC9799/9800, OLEC9875/9876 and OLEC9831/9832, respectively. To construct pSpy1C (based on pIB166) and pSpy3K (based on pBAV1K-T5-*gfp*), the previously mutated plasmid backbones were amplified with the primer pairs OLEC9773/9774 and OLEC9829/9830, respectively. The multiple cloning site (MCS) featuring restriction enzyme recognition sites for EcoRI, XbaI, SpeI and PstI also contains a reporter cassette encoding the *mrfp* gene controlled by the P_*lac*(*Eco*)_ promoter. The P_*lac*(*Eco*)__*mrfp* fragment was amplified from pBS3C*lux* (Radeck et al., 2013) using OLEC9781/9782. pBS3Clux was a gift from Thorsten Mascher (Addgene plasmid # 55172). Restriction enzyme recognition sites were attached by PCR using OLEC9775/9776. The final MCS was then inserted into the backbones using Golden Gate Assembly.

For the construction of pSpy1K and pSpy1E, the pSpy1C backbone was amplified using OLEC9826/9776. The kanamycin and erythromycin resistance cassettes were amplified using OLEC9824/9825 and OLEC9827/9828 from pIB184-Km and pIB185, respectively. Golden Gate Assembly was performed to introduce the marker genes into the backbones.

To construct pSpy2K, we used OLEC9777/9778 to amplify the replicon and resistance cassette from pIB184-Km (*Esp*3I removed). For pSpy2E, the replicon and resistance cassette were amplified from pIB185 by PCR using OLEC9779/9780. The MCS was cloned into the backbones using Golden Gate Assembly. To create pSpy2C, the backbone of pSpy2K and the chloramphenicol resistance cassette from pIB166 were amplified by PCR using the primers OLEC9877/9878 and OLEC9835/9836, respectively. The fragments were joined using Golden Gate Assembly. As no colonies were obtained after transformation of the pSpy2 series, we hypothesized that *S. pyogenes* was not transformable with these plasmids, and exchanged the pIB185-derived replicon with the pAMβ1 replicon from pMSP3535. Primers OLEC14365/14366 were used to amplify a backbone part of pSpy2C (not functional) and OLEC14367/14368 to amplify the replicon from a pMSP3535 derivative (Bryan et al., 2000). The fragments were joined by Gibson Assembly^®^ to create pSpy2C. For pSpy2E, we amplified part of the backbone from pSpy2E (not functional) using OLEC14365/14369 and the pAMβ1 replicon from the pMSP3535 derivative using OLEC14370/14368. The fragments were again joined by Gibson Assembly^®^. We were unable to assemble the repaired pSpy2K plasmid.

For pSpy3C and pSpy3E, the plasmid backbone of pSpy3K was amplified with primers OLEC9833/9834. Overhangs for cloning were attached to both resistance cassettes, *cat* and *ermB*, using the oligo pairs OLEC9835/9836 and OLEC9837/9838, respectively. The previously amplified cassettes used for pSpy1C and pSpy1E served as templates. The plasmids were assembled using Golden Gate Assembly.

#### 2.4.2 Integrative plasmid backbones

We designed a set of integrative plasmids for use in *S. pyogenes* SF370 that integrate into different genomic loci as described in Table 1.

pSpy0C2 was cloned through Golden Gate Assembly using the type II restriction enzyme *Esp*3I (NEB). The MCS-*cat* fragment and the pUC origin were amplified using OLEC10311/10312 from pSpy1C and OLEC10315/10316 from pUC19-*mKate2∼ssrA*, respectively. Up- and downstream homologous regions of *amyA* were amplified from *S. pyogenes* genomic DNA using OLEC10930/10931 and OLEC10932/10933, respectively. All fragments were joined using Golden Gate Assembly. To construct pSpy0C4, the MCS-*cat* fragment and the pUC origin were amplified using OLEC11471/11472 from pSpy1C and OLEC11480/11476 from pUC19-*mKate2∼ssrA*, respectively. Up- and downstream homologous regions of the *sagB* gene were amplified from *S. pyogenes* genomic DNA using OLEC11473/11479 and OLEC11477/11535, respectively. Gibson Assembly^®^ was used to ligate all fragments. To clone pSpy0K6, the MCS was amplified from pSpy0C4 using OLEC14327/14328. The kanamycin resistance cassette was amplified from pLZ12Km2-P*23*R:TA:*ffluc* using OLEC14329/14330. Up- and downstream homology arms of *SPy_1078* were amplified from *S. pyogenes* genomic DNA with OLEC14331/14332 and OLEC14335/14336, respectively. The pUC origin was amplified from pUC19*-mKate2∼ssrA* using OLEC14333/14334. Up- and downstream homology arms and the origin were joined by LFH-PCR using OLEC14337/14335. The fragments were ligated using Gibson Assembly^®^. Additionally, we designed the integrative plasmid pSpy0C5 targeting the transcriptionally silent gene *SPy_1930* in *S. pyogenes* SF370, but were unable to obtain clones even with two different designs of the plasmid, potentially due to a stabilizing toxin-antitoxin locus just upstream (Supplementary Figures S1A, D).

p7INT was a gift from Prof. Michael Federle (University of Illinois Chicago, United States) (McShan et al., 1998). To obtain p7INT.1 featuring the same MCS as the remaining plasmids of the collection, we amplified the p7INT backbone using OLEC14308/14309. The MCS was amplified using OLEC14306/14307 from pSpy1C and both fragments were joined using Gibson Assembly^®^. A restriction enzyme recognition site in the integrase open reading frame was removed by PCR mutagenesis using OLEC14310/14311 to ensure that MCS restriction sites occur only once.

All plasmids, except p7INT (Supplementary Figure S1B) and p7INT.1, were linearized using *Alw*44I prior to transformation into *S. pyogenes* SF370 to favor double-crossover recombination (Heap et al., 2012; Wu et al., 2019). Integration of the plasmids was verified by PCR and the integration sites and distinct virulence genes (*ropB*, *csrRS*, *mga*) were sequenced using Sanger sequencing. Additionally, whole genome sequencing of all strains harboring the integrative plasmids was performed using Nanopore sequencing.

#### 2.4.3 Luciferase reporter plasmids

For the construction of the reporter plasmids harboring the *ffluc* reporter gene (*Photinus pyralis*)*,* we used either the Golden Gate Assembly strategy using *Eco*31I (Thermo Scientific™) or Gibson Assembly^®^.

To construct pSpy1C-*ffluc* (control), the *ffluc* gene was PCR-amplified using the oligos OLEC10182/10028 from pLZ12Km2-P*23*R:TA:*ffluc,* a gift from Thomas Proft (Addgene plasmid # 88900) (Loh and Proft, 2013), and assembled into the linearized (*Eco*31I) pSpy1C plasmid. To assess the activity of different constitutive promoters, the promoters P_*23*_ (*L. lactis*), P_*veg*_ (*B. subtilis*), P_*gyrA*(*Spy*)_ (*S. pyogenes*) and P_*xylS2*_ (synthetic promoter) were cloned upstream of *ffluc* into pSpy1C as detailed in Supplementary Table S5. Each inducible regulatory element was placed upstream of *ffluc* in pSpy1C as described in Supplementary Table S6. For some of the PCRs, we used the following plasmids as template: pEU8517 (Bugrysheva and Scott, 2010), pMSP3535 donated by Gary Dunny (Addgene plasmid # 46886) (Bryan et al., 2000), pPEPY-PF6-lacI (Addgene plasmid # 85589) and pJWV102-PL-dCas9 (Addgene plasmid # 85588) donated by Jan-Willem Veening (Liu et al., 2017).

To improve the signal-to-noise ratio during luciferase assays, an additional terminator was introduced upstream of all promoter-*ffluc* reporter fusions by PCR mutagenesis using OLEC11104/11105.

#### 2.4.4 Reporter plasmids featuring mNeongreen and mKate2

The codon-optimized (Grote et al., 2005) reporters *mKate2* and *mNeongreen* (gene synthesis by GenScript) were assembled into p7INT and other backbones as described in Supplementary Table S7. For the cloning of *mNeongreen* and *mKate2* into pSpy0C4, inserts and backbone were digested with *Eco*RI and *Pst*I (Thermo Scientific™). The fragments were ligated using T4 DNA Ligase (Thermo Scientific™), creating pSpy0C4-*mNeongreen ∼ ssrA* and pSpy0C4-*mKate2∼ssrA*.

The DNA sequence coding for the last three amino acids of the native SsrA tag sequence downstream of the *mNeongreen* reporter gene was modified from LAA (WT) to LDD or ASV by PCR using the primer pairs OLEC13042/13043 and OLEC13040/13041, respectively. As control, a stop codon (*) was introduced upstream of the *ssrA* tag to prevent any SsrA-driven proteolytic degradation. The stop codon was introduced by PCR using OLEC13044/13045 and OLEC13046/13047 for *mNeongreen* and *mKate2* reporter constructs, respectively.

#### 2.4.5 Construction of pERASE and deletion of *sagA* using pERASE-*ΔsagA*

Fragments for the assembly of pERASE, including the pUC origin (from pUC19-*mKate2∼ssrA*), erythromycin resistance cassette (from p7INT), the MCS (from pSpy0C4) and the pheS* counterselection marker under control of P_*veg*_ were amplified using primers OLEC14398/14523, OLEC14522/14403, OLEC14404/14405 and OLEC14524/14525, respectively. Fragments were assembled using Gibson Assembly^®^ and checked by Sanger sequencing. An optimal RBS was added upstream of pheS* by PCR mutagenesis using OLEC14726/14727. The nucleotide sequence for pheS*, encoding a variant of the phenylalanyl-tRNA-synthetase (α-subunit), was adapted to be less homologous to the native *pheS* locus in *S. pyogenes* to prevent recombination events. The pheS* DNA sequence was obtained by gene synthesis (service provided by GenScript).

To construct pERASE-*ΔsagA* for the exemplary deletion of *sagA*, we amplified the pERASE backbone using primers OLEC14883/14884. Fragments up and-downstream of *sagA* were amplified from *S. pyogenes* genomic DNA with OLEC14875/14876 and OLEC14877/14878, respectively. The three amplicons were assembled using Gibson Assembly^®^ and correct assembly was analyzed by restriction digestion and Sanger sequencing.

To delete *sagA*, pERASE-*ΔsagA* was transformed as described above into sucrose-competent *S. pyogenes* SF370 cells and integration events were selected on TSA blood agar plates with antibiotic selection at 37°C and 5% CO_2_ for 48 h. Clones were re-streaked onto selective TSA blood agar plates and incubated overnight. Single colonies were inoculated into THY medium without antibiotic selection and incubated until exponential growth phase (OD_620nm_ = 0.25) at 37°C and 5% CO_2_. Cultures were passaged three times in liquid THY (1:1,000 dilution) without antibiotics to enable curing of the plasmid. Dilutions (10^–3^) were plated on THY agar plates with 5 mM PCPA (4-chlor-DL-phenylalanine) and incubated overnight at 37°C and 5% CO_2_ to select for clones that lost plasmid. Around 70 clones were re-streaked onto TSA blood agar plates with or without antibiotic selection and on THY agar plates with PCPA and incubated for 48 h. Deletion of *sagA* will result in decreased hemolysis on TSA blood agar plates. Eight clones that demonstrated decreased hemolysis and three clones that did not were analyzed by PCR using OLEC14875/14878. For three of the eight clones indicating loss of *sagA* in the PCR, we confirmed the deletion by Sanger sequencing.

### 2.5 Luciferase assay to determine promoter activities

Plasmids were transformed into electrocompetent cells of the *S. pyogenes* SF370 wildtype or the LacI-expressing strain (SF370 *attB* (T12)::p7INT-P_*veg*__*lacI*). Cultures were started as described for the growth curve experiments and incubated at 37°C with 5% CO_2_; OD_620nm_ and luminescence were measured every 30 min in a plate reader (Cytation 3, BioTek). To detect luminescence, 10 μL of a 1 mg/mL luciferin stock solution were added to the samples immediately before the measurement (integration time = 1s, read height = 1 mm, white 96-well plate).

To test the activity of the inducible promoters over the course of growth, the corresponding inducer compounds were added right at the start of the experiment (t_0_). The promoters P_*nisA*_, P_*tet*_, P_*Zn*_, P_*lac(Spn)*_ and derivatives P_*lac*__(1–3)_, and P_*tre*_ were induced with 0.75 μg/mL nisin, 10 ng/mL anhydrotetracycline (AHT), 80 μM ZnSO_4,_ 1 mM IPTG, and 0.4% trehalose, respectively. MilliQ water served as a control. For induction of the erythromycin-inducible riboswitch, we added 3 ng/mL erythromycin or 0.1% ethanol as control.

To assess the dose-response of the inducible promoters, day cultures of strains harboring the control plasmid (without promoter) or the reporter plasmid with the inducible promoter were grown until the exponential growth phase (OD_620nm_ = 0.25). The cultures were split and induced with a range of concentrations of the inducer compound as well as 0.1% ethanol or distilled water as control. Growth and luminescence were measured as described above every 30 min for 3 h. All experiments were performed in biological triplicates.

To determine statistically significant differences between promoter activities, a Brown-Forsythe and Welch ANOVA test for multiple comparisons was performed.

### 2.6 Development of the RPMI-based chemically defined medium for plate reader-based fluorescence measurements

To develop a new chemically defined medium (CDM) with decreased autofluorescence, we utilized the cell culture medium RPMI1640 with glutamine and without phenol red. We assessed the growth of *S. pyogenes* SF370 in different RPMI1640-based media compositions as described under “growth curves” with minor adaptations. An additional washing step with 1x PBS solution was added before cell pellets were resuspended in the test media to remove any remaining THY medium. The initial recipes tested can be found in Supplementary Table S8.

The fluorescence signals of *S. pyogenes* cells expressing mNeongreen were compared in RPMI4Spy, THY and CDM as follows. Overnight cultures of the reporter and control strains were harvested by centrifugation at 4,500 x g for 10 min, washed with sterile 1x PBS solution and adjusted to an OD_620nm_ of 0.02 in the different media. Cultures were distributed into a black 96-well plate (in duplicates) with clear bottom and incubated in a plate reader (Synergy H1, BioTek). Growth (OD_620nm_) and mNeongreen fluorescence (500 nm ex, 530 nm em) were measured every 20 min for 15 h. For the comparison of mKate2 signals from different genomic loci, we proceeded as described above, but measured fluorescence using 588 nm ex, 633 nm em. Experiments were performed in biological triplicates. To compare the mKate2 signal from different genomic loci, statistical significance was analyzed using a Friedman test for multiple comparisons [n.s. = not significant, *p* < 0.05 = significant (*)].

### 2.7 Analyzing the influence of SsrA tag variants on mNeongreen stability

To analyze the effect of the SsrA tag variants on mNeongreen protein stability, we measured the decrease in fluorescence signal after inhibiting translation using spectinomycin. A growth curve experiment was set up as described previously with strains harboring the mNeongreen reporter fused to different SsrA tag variants as well as the control strain (no promoter) in RPMI4Spy (Supplementary Table S1). The strains were incubated in a plate reader, and growth (OD_620nm_) and fluorescence (500 nm ex, 530 nm em) were measured every 20 min until the exponential growth phase (OD_620nm_ = 0.2). Then, 100 μg/mL spectinomycin was applied to the samples, and growth and fluorescence were measured for 10 more hours. Experiments were performed in biological triplicates.

### 2.8 Fluorescence microscopy

Overnight cultures of the mNeongreen and mKate2 reporter strains as well as the corresponding control strains (no promoter) (Supplementary Table S1) were used to prepare day cultures in RPMI4Spy as described above (final OD_620nm_ of 0.02). Cultures were grown at 37°C and 5% CO_2_ until the exponential growth phase (OD_620nm_ = 0.25). Then, 5 μL of each culture were mounted onto an agarose pad (0.75% agarose in 1x PBS) for fluorescence microscopy. Microscopy images were obtained using an inverted fluorescence microscope (Leica Dmi8, DFC9000 GT VSC-D6212 camera) using a 100x phase-contrast lens, and exposure times of 300 m (mNeongreen, control) and 2 s (mKate2, control), respectively. Images were edited for publication using the Leica Application Suite X (LAS X) Software from Leica Microsystems. Experiments were performed in biological triplicates.

### 2.9 Identification of transcriptionally silent sites

RNA reads were mapped to the respective reference sequences of *S. pyogenes* strains SF370 (NCBI assembly GCF_000006785.2) and 5,448 (NCBI assembly GCF_900619585.1) using BWA mem2 V2.2 (Vasimuddin et al., 2019) and Samtools V1.15.1 (Li et al., 2009), each with default settings. The coverage for each position and RNA read file was extracted using the Python library Pysam V0.22.0 and its function pileup (Li et al., 2009). Every position of each coverage dataset was normalized by dividing its coverage by the median of the upper quartile of the coverage values of the whole dataset. Values above 1 were set to 1. For each position, the average across all strain-specific normalized coverages was calculated. Based on these strain-specific datasets, candidate regions were identified by masking prophage regions and annotated genes showing an average normalized expression of >0.02, including 250 bp upstream of every expressed gene. The remaining unmasked consecutive positions were categorized into unexpressed genes based on available strain annotation, unexpressed intergenic regions and true zero coverage regions. Unexpressed refers to a normalized average coverage of ≤0.02. All identified regions were further filtered for a minimum size of 100 bp (Supplementary Tables S9, S10). Finally, candidate regions were identified in companion strains by mapping region sequences using BWA mem2 and Samtools, each with default settings and extracting companion sequences utilizing Python’s Pysam library as described above. Then, every pair of sequences was aligned to each other using Biopython’s pairwise2 global alignment module (V1.81) followed by reporting the Hamming distance normalized by sequence length (Cock et al., 2009). Pairs with a score <90%, arbitrarily chosen, were considered paralogs (Supplementary Table S11).

The code is provided on GitHub (https://github.com/MPUSP/lautenschlaeger_silent_sites). External RNA-seq datasets analyzed for the identification of transcriptionally silent sites in *S. pyogenes* SF370 and M1T1 5,448 are partially available in the European Nucleotide Archive (ENA) or the gene expression omnibus (GEO) via the following SRP/DRP accession numbers: SRP066922, DRP008790, SRP389502, DRP007263, SRP267980, SRP266954, DRP004959, SRP073901, SRP119757 and SRP390529 or the NCBI BioProject identifiers: PRJNA304766, PRJDB13957, PRJDB8873, PRJNA640594, PRJNA638918, PRJDB8158, PRJNA319617, PRJNA412519, PRJNA297518. Datasets published by our laboratory were included, accessible under GEO accession numbers GSE84641, GSE40198 or BioProject accession PRJNA193607; at NCBI under accession number SRP149896.

### 2.10 Nanopore sequencing for strain validation

Genomic DNA of strains harboring the integrative plasmids pSpy0C2, pSpy0C4, pSpy0K6 and p7INT.1 was extracted using the Monarch^®^ HMW DNA Extraction Kit for Tissue (NEB) according to the manufacturers protocol for Gram-positive bacteria (low input) and sheared 15x with a G27 needle (B.Braun). Barcoded libraries were prepared using the Native Barcoding Kit (SQK-NBD114.24, Oxford Nanopore Technologies) according to the manufacturer’s protocol with 400 ng genomic DNA per sample, loaded on a MinION Flow Cell (R10.4.1, FLO-MIN114) and sequenced for 72 h on a MinION Mk1C (MinKNOW v23.04.8). POD5 files were basecalled and demultiplexed with the super accuracy model (v4.3.0) using Dorado (v0.5.0) with the “barcode-both-ends” option to reduce false-positives during barcode classification.

We developed a Snakemake workflow for the identification of variants in bacterial genomes (https://github.com/MPUSP/snakemake-ont-bacterial-variants) (Mölder et al., 2021). In brief, reads were filtered using Filtlong (v0.2.1, available on GitHub: https://github.com/rrwick/Filtlong) with options “keep_percent 90 --min_length 500 --min_mean_q 90” and aligned to the reference genome using NGMLR (v0.2.7) with default settings (Sedlazeck et al., 2018). Alignments files were sorted and indexed using Samtools (v1.9) and used for structural variants calling using Sniffles2 (v2.2) with standard settings and cuteSV (v2.1.0) with parameters suggested for ONT data (Li et al., 2009; Sedlazeck et al., 2018; Jiang et al., 2020; Smolka et al., 2024). Single nucleotide variants were identified using Clair3 (v1.0.5) with the alignment generated by NGMLR and using Medaka (v1.11.3, available on GitHub: https://github.com/nanoporetech/medaka), which internally uses minimap2 for read alignment (Sedlazeck et al., 2018; Zheng et al., 2022). Since we noticed putative assembly artefacts in the rRNA operons of the reference genome (data not shown), we additionally filtered the resulting variant files using VCFtools (v0.1.16) (Danecek et al., 2011) to exclude rRNA-tRNA operon regions (±10 bp): 17,055..29,081; 79,274..85,457; 264,339..269,670; 1,330,440..1,336,193; and 1,557,792..1,583,127 of NC_002737.2. NanoPlot (v1.42.0) and MultiQC (v1.19) were used for read quality control (Ewels et al., 2016; De Coster and Rademakers, 2023). Finally, identified variants were reported in a custom HTML report (RMarkdown v2.25) with alignments visualized for relevant regions using igv-reports (v1.10.0, available at GitHub: https://github.com/igvteam/igv-reports) (Robinson et al., 2011).

As both cuteSV and Sniffles2 had difficulties to accurately identify allelic replacements, we provided the reference genome of *S. pyogenes* SF370 (NC_002737.2) and the sequence of the transformed integrative plasmid to facilitate the detection of allelic replacements as translocations. Additionally, we re-performed the analysis using the expected genotype as reference.

## 3 Results

### 3.1 Standardized replicative plasmids for *S. pyogenes*

We have created a collection of plasmids for *S. pyogenes* that allows an easy transfer of inserts between backbones featuring different antibiotic markers and origins of replication with varying copy numbers (Table 1; Supplementary Table S3). All plasmids share the same MCS containing the *mrfp* gene under control of the P_*lac*(*Eco*)_ promoter that is flanked by restriction enzyme recognition sites and M13 primer binding sites. In *E. coli*, the mRFP reporter enables screening for clones harboring the plasmid with the desired insert, which appear as white colonies, while clones harboring the empty plasmid appear red. The M13 primer binding sites can be used for colony PCR and subsequent sequencing of the desired insert. We selected the replication origins pSH71, pAMβ1 and a previously engineered version of pWV01, pVW01*. While pSH71 and pWV01* are functional in both Gram-negative and Gram-positive bacteria, pAMβ1 is restricted to Gram-positive bacteria and requires the addition of a Gram-negative origin to allow for replication in *E. coli* (Duarte and Monteiro, 2021). The replicons pSH71 and pWV01* have been reported as high-copy number replicons in several Gram-positive bacteria, with pSH71 being present at around 200 copies per cell in *L. lactis* and pWV01* at 68 copies per cell in *B. subtilis* (Bryksin and Matsumura, 2010; Tauer et al., 2014). The original pAMβ1 replicon is commonly a low-copy replicon, with copy number between 7-9 copies per cell reported for *Streptococcus lactis* (Simon and Chopin, 1988). For plasmid maintenance, we selected three antibiotic resistance cassettes (*cat*, *ermB* and *aph(3′)-IIIa*) that are functional in both *E. coli* and *S. pyogenes*.

**FIGURE 1:**
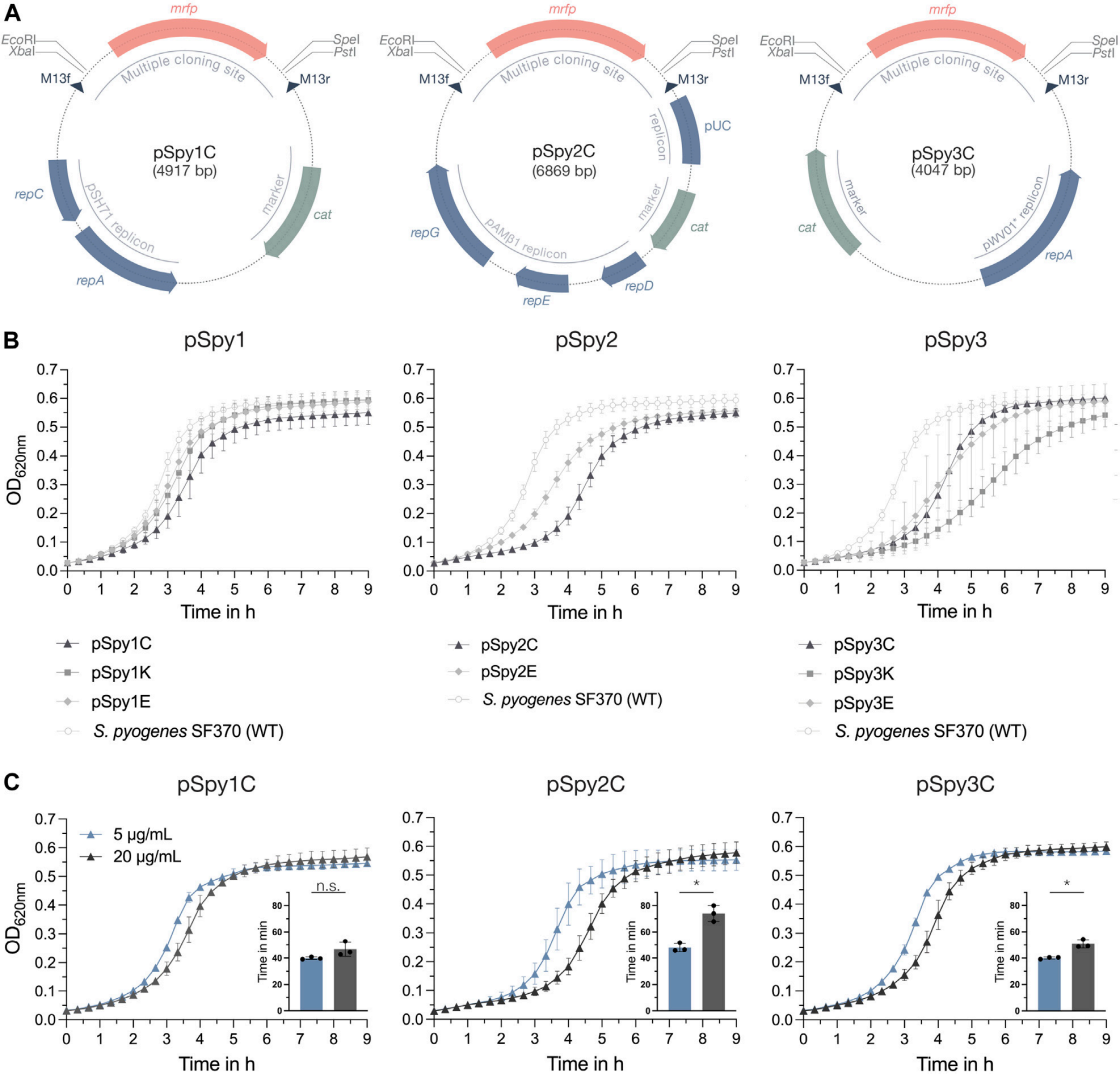
Novel replicative plasmids for use in *S. pyogenes*. **(A)** Representative schematic maps of the replicative plasmids pSpy1C (high-copy pSH71 replicon), pSpy2C (low-copy pAMβ1 replicon) and pSpy3C (engineered high-copy pWV01* replicon) for use in *S. pyogenes*. The origin of replication is indicated in blue, while the resistance cassette (*cat*) is highlighted in sage green. The specific restriction sites of the MCS flank the *mrfp* reporter gene, the expression of which is driven by P_*lac*(*Eco*)_ *,* enabling red-white screening of single colonies during subcloning in *E. coli*. The dark blue triangles indicate the binding sites for the M13f and M13r standard primers. **(B)** Growth, shown as OD_620nm_, of *S. pyogenes* SF370 transformed with the different replicative plasmids in THY medium with antibiotic selection. Growth curves of cultures harboring pSpy1C (triangle), pSpy1K (square) and pSpy1E (diamond) are depicted on the left, while the graph in the center shows the growth of *S. pyogenes* transformed with pSpy2C (triangle) and pSpy2E (diamond). The graph on the right displays the growth of *S. pyogenes* harboring pSpy3C (triangle), pSpy3K (square) and pSpy3E (diamond). Growth of the *S. pyogenes* SF370 wildtype in THY medium without antibiotic selection is indicated using round data points with a white fill. **(C)** Adaptation of the selective chloramphenicol concentration for each plasmid backbone. Shown are differences in the growth profiles and respective generation times in min (embedded bar graph) when *S. pyogenes* harboring pSpy1C, pSpy2C or pSpy3C is grown in the presence of 5 μg/mL (blue) or 20 μg/mL (anthracite) chloramphenicol. Growth is depicted as OD_620nm_ over time. Statistical differences between generation times were calculated using a two-sample t-test [n.s. = not significant, *p* < 0.05 = significant (*)]. Experiments were performed in biological triplicates and each measurement in technical duplicates.

The constructed pSpy1 plasmid series is based on the rolling-circle replicon (RCR) pSH71 and was combined with antibiotic resistance cassettes encoding for chloramphenicol (pSpy1C) (Figure 1A; Table 1), kanamycin (pSpy1K) and erythromycin (pSpy1E) (Biswas et al., 2008). Based on the theta replicon pAMβ1 (Clewell et al., 1974; Cho et al., 2019), we constructed the pSpy2 series comprising plasmids pSpy2C (Figure 1A; Table 1) and pSpy2E, featuring a chloramphenicol and erythromycin resistance cassette, respectively. We were unable to obtain the pSpy2K plasmid. Lastly, we designed the pSpy3 series using the RCR pWV01* (Bryksin and Matsumura, 2010), resulting in pSpy3C (Figure 1A; Table 1) (chloramphenicol resistance), pSpy3K (kanamycin resistance) and pSpy3E (erythromycin resistance).

All replicative plasmids were transformed into *S. pyogenes* SF370 and analyzed for their effect on growth in THY medium with selection. Our results showed that bacteria harboring plasmids of the pSpy1 series grew very similar (Figure 1B). Interestingly, bacteria harboring pSpy2C from the pSpy2 series had a longer lag phase than cells harboring pSpy2E (Figure 1B). *S. pyogenes* cells harboring pSpy3C and pSpy3E from the pSpy3 series had very similar growth profiles, whereas bacteria harboring pSpy3K showed a longer lag phase (Figure 1B). Plasmids can entail a fitness cost and consequently result in reduced bacterial growth. The effect on growth has been linked to different plasmid features, such as plasmid size, encoded resistance genes, copy number or high expression levels of encoded proteins (Mi et al., 2016). As the variations in the growth profiles seemed to be based on the different antibiotic resistances, we hypothesized that optimizing the selective antibiotic concentration could improve the growth of the strains. To demonstrate this, we grew *S. pyogenes* harboring pSpy1C, pSpy2C and pSpy3C as well as the wildtype without any plasmid in the presence of different concentrations of chloramphenicol. Due to the high copy number, the growth of *S. pyogenes* harboring pSpy1C was less affected at higher chloramphenicol concentrations and generation times were similar at 5 μg/mL and 20 μg/mL (Figure 1C; Supplementary Figure S2). In line with this, we observed a significantly shorter generation time of *S. pyogenes* harboring the low-copy plasmid pSpy2C at concentrations of 5 μg/mL chloramphenicol (Figure 1C), which is the minimum concentration required for growth inhibition of the wildtype (Supplementary Figure S2). Growing *S. pyogenes* harboring pSpy3C using 5 μg/mL chloramphenicol also significantly shortened the generation time (Figure 1C). Thus, adjustments to the concentration of the selective antibiotic to the plasmid copy number are required for optimal experimental conditions.

### 3.2 Novel integrative plasmids targeting specific loci in the streptococcal genome

**FIGURE 2:**
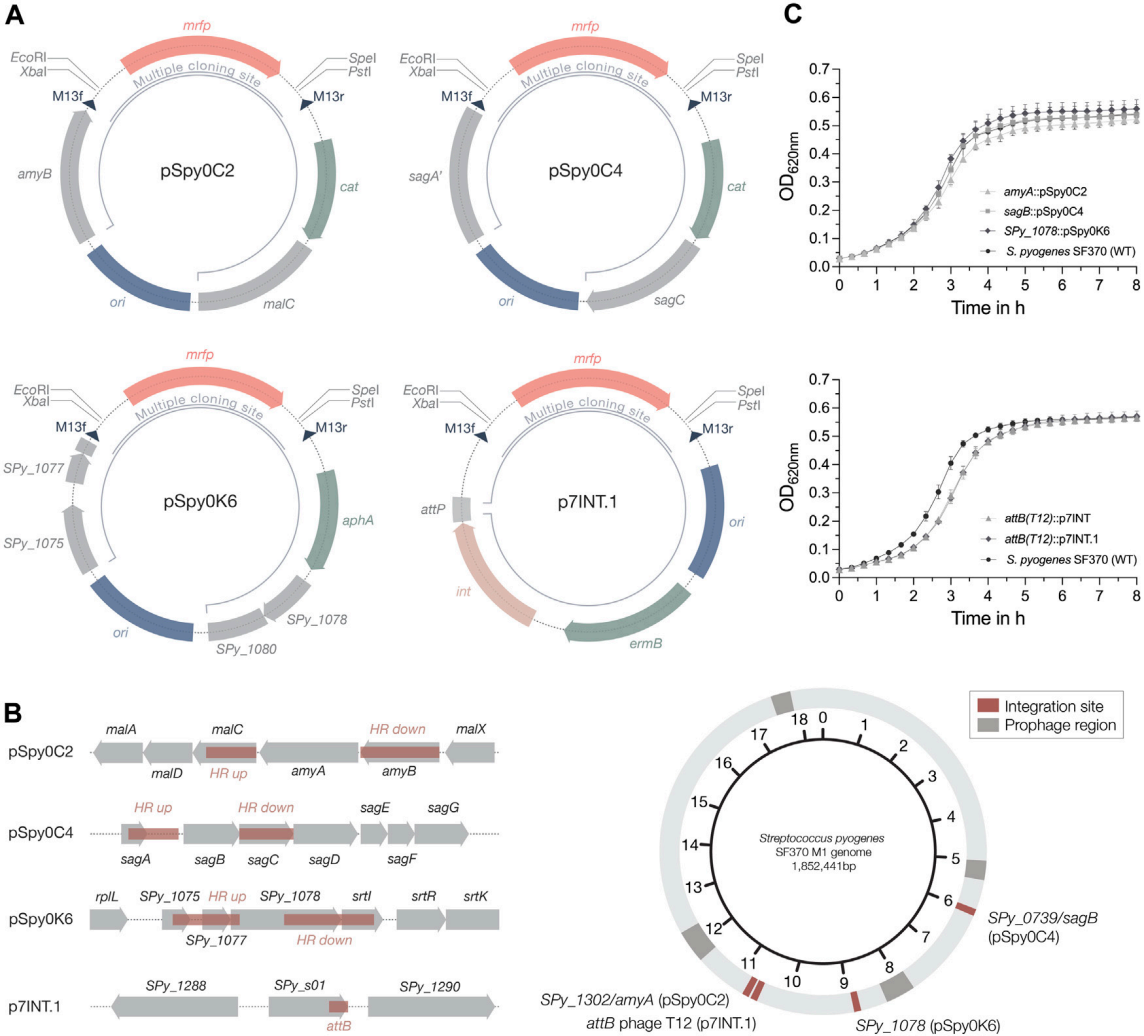
Plasmids for site-specific integration into the streptococcal genome. **(A)** Schematic plasmid maps of the three integrative plasmids designed for *S. pyogenes* SF370 and the integrase-based plasmid p7INT.1. The pUC replicon for propagation in *E. coli* is marked in blue. Resistance cassettes for chloramphenicol (*cat*), erythromycin (*ermB*) and kanamycin (*aph(3′)-IIIa*) are highlighted in sage green. DNA sequences with homology to the genome of SF370 are shown in grey. Restriction sites of the MCS flank the *mrfp* reporter gene under the control of P_*lac*(*Eco*)_, enabling red-white screening of clones containing the desired insert in *E. coli*. Dark blue triangles indicate the binding sites for the M13f and M13r standard primers. The inner black circular line marks the part of the plasmid that will integrate into the streptococcal genome. For the p7INT derivative p7INT.1, the pale pink color indicates the integrase gene (*int*) with the corresponding attachment site of prophage T12 (*att*P) marked in grey. **(B)** Left: Linear view of the genomic regions targeted by the integrative plasmids. Sites used for homologous recombination or insertion mediated by the integrase (*att*B) are marked in red. Genes are indicated in grey. Right: Circular map of the *S. pyogenes* SF370 genome indicating the localization of the different plasmid integration sites in red. Prophage regions are marked in dark grey. **(C)** Growth of *S. pyogenes* SF370 strains, shown as OD_620nm_, harboring different integrated plasmids in THY medium without antibiotics. The effect on growth of the integrative plasmids pSpy0C2 (triangle), pSpy0C4 (square) and pSpy0K6 (diamond) is shown in the top graph, while the bottom graph displays the growth of the strains harboring p7INT (triangle) and p7INT.1 (diamond) in the genome. An exemplary growth curve of the *S. pyogenes* SF370 wildtype is plotted (WT; black circles). Experiments were performed in biological triplicates and each measurement in technical duplicates.

Previously, only two integrative plasmids have been described for use in GAS, pFW11e and p7INT (McShan et al., 1998; Park et al., 2003). To enable the stable integration of multiple genes of interest into the streptococcal genome at different loci, we explored alternative integration sites. We first designed two integrative plasmids, pSpy0C2 and pSpy0C4, targeting the genes *amyA* and *sagB*, respectively, for insertion by homologous recombination (Figures 2A, B; Table 1). Disruption of the *amyA* or *sagB* locus will enable positive integration events to be screened by selection on specific TSA plates. Whereas allelic replacement of *amyA* leads to inhibited starch degradation on starch plates, the deletion of *sagB* results in the loss of hemolysis on TSA blood plates.

We performed growth curve experiments to assess the effect of plasmid integration on growth. Strains harboring the novel integrative plasmids grew very similar compared to the *S. pyogenes* wildtype in THY medium without antibiotics, suggesting that these allelic replacements have no effect on growth under this condition (Figure 2C). To propose alternative integration sites that do not disrupt transcribed genes, we screened the genome for transcriptionally silent sites suitable for integration, similar to the reported integrative vectors for *Streptococcus pneumoniae* (Keller et al., 2019). To do this, we used published RNA-seq datasets from *S. pyogenes* SF370 and the more clinically relevant M1T1 5,448 strain grown under various conditions (Supplementary Tables S9, S10). We then analyzed, which of the obtained sites are present in both strains, and could therefore be targeted using the same plasmid (Supplementary Table S11). Among those sites, we identified the gene *SPy_1078* as a transcriptionally silent site in *S. pyogenes* SF370 (Supplementary Table S9). This gene contains frameshift mutations resulting in several early stop codons. Consequently, we designed the plasmid pSpy0K6 featuring homologous regions to target *SPy_1078* for allelic replacement (Figures 2A, B; Table 1). Following successful integration of pSpy0K6, we conducted growth curve experiments. The strain harboring pSpy0K6 grew very similar to the wildtype in THY medium without antibiotics, indicating that disruption of *SPy_1078* had no effect on growth under the conditions tested (Figure 2C).

To make the already available p7INT plasmid (Supplementary Figure S1B) compatible with our plasmid collection, we exchanged the MCS featuring *lacZ* to fit our MCS design, resulting in p7INT.1 (Figure 2A; Table 1). When growing the strains in THY medium without antibiotics, we noticed a growth delay of approximately 20 min for strains harboring p7INT or its derivative p7INT.1 compared to the wildtype (Figure 2C). This observation could depend on the growth conditions tested and is not caused by plasmid integration at the tmRNA locus as we have shown that SsrA-mediated proteolysis is functional in strains harboring p7INT-based constructs (Figure 6). The specific genome integration of the pSpy0 series and p7INT.1 was validated by nanopore sequencing for two independent clones obtained.

Next, we sought to determine the host range of the newly designed integrative plasmids. We conducted multiple sequence alignments of the *amyA*, *sagB* and *SPy_1078* integration sites found in various streptococcal serotypes. To this end, we compared the SF370 and M1T1 5,448 genomic regions to genome sequences representative of M3, M4, M5, M6, M12, M18, M28, M44, M49 and M53 serotypes. We found that the *amyA* locus of SF370 is 100% conserved in M1T1 5,448, 99.88% conserved in M28 GAS6180% and 99.95% conserved in the M4 GAS10750 strain (Supplementary Table S12), whereas *amyA* is not encoded in other serotypes. The *sagB* locus is well conserved between SF370, M1T1 5,448 and M28 GAS6180 (100% identity) (Supplementary Table S13). While the SF370 *sagB* locus is highly conserved in M49 NZ131 (99.51% identity), M12 MGAS9429 (99.57% identity), M4 MGAS10750 (99.76%) and M44 STAB901 (99.57%), we observed less conservation (<85% identity) with other serotypes (Supplementary Table S13). *SPy_1078*, similar to *amyA*, does not seem to be widely distributed among different serotypes. We found high conservation of this locus between SF370 and M1T1 5,448 (99.95% identity), M44 STAB901 (99.27% identity) and M12 MGAS9429 (99.37% identity) (Supplementary Table S14). Our analysis indicates that the integrative plasmids are most likely functional in other serotypes with 100% sequence conservation, but may also be applicable in serotypes with a slightly deviating DNA sequence homology from the integration site (e.g., > 99% sequence conservation).

Taken together, we here present three novel integrative plasmids that can be used in combination with the p7INT.1 plasmid to create strains harboring multiple genetic constructs of interest, e.g., for complex regulatory switches.

### 3.3 Characterization and adaptation of constitutive promoters

**FIGURE 3:**
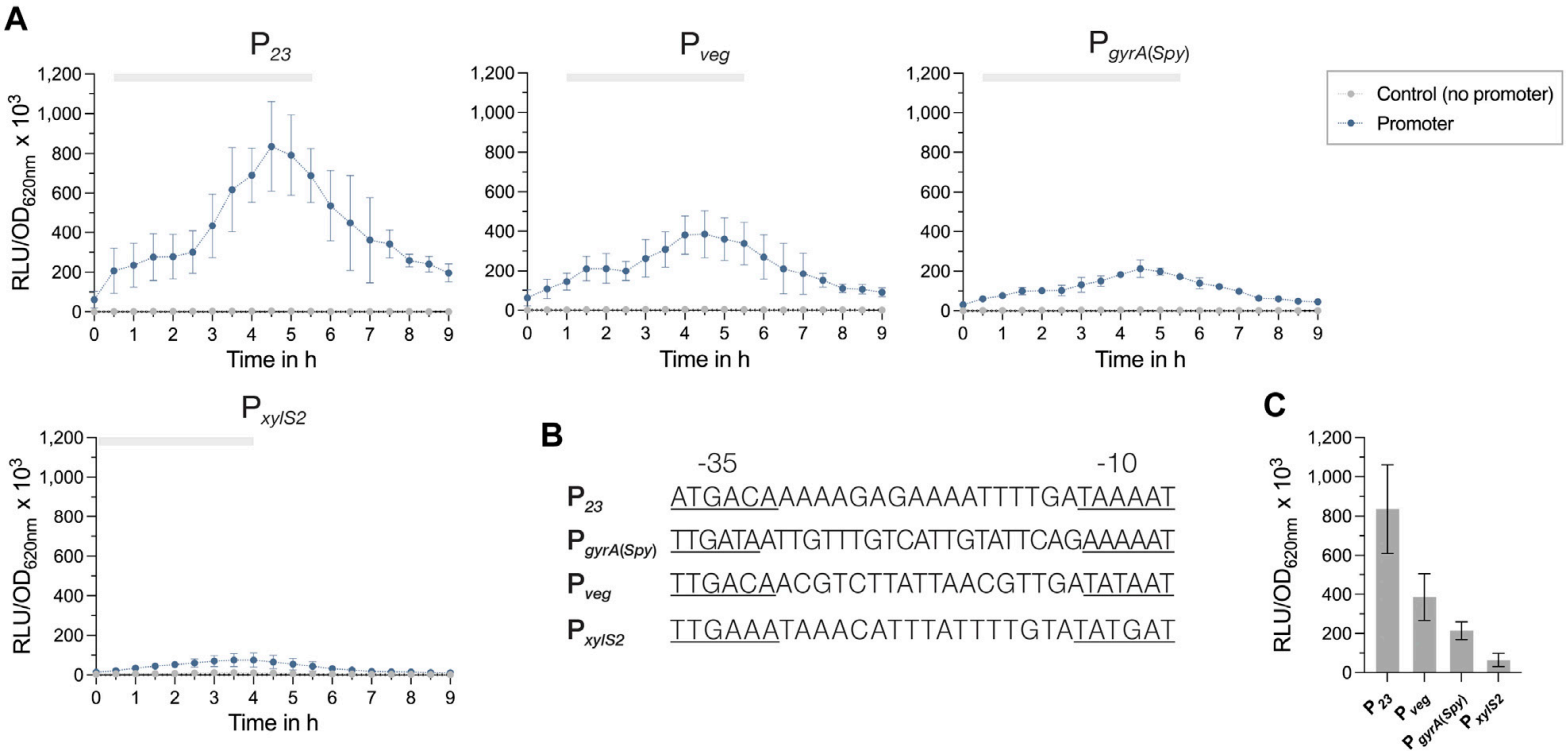
Activities of constitutive promoters for use in *S. pyogenes*. **(A)** Promoter activities of the four tested constitutive promoters P_*23*_, P_*veg*_, P_*gyrA*_ and P_*xylS2*_ in *S. pyogenes* SF370 grown in THY medium are shown as relative luminescence units normalized by optical density (RLU/OD_620nm_). Promoter activities of the reporter strains (blue) and the control without a promoter (grey) were measured every 30 min over the course of growth using the luciferase assay. The grey bar at the top of each graph marks the timespan of the exponential growth phase. **(B)** DNA sequences of the constitutive promoters P_*23*_, P_*veg*_, P_*gyrA*_ and P_*xylS2*_ comprising the −35 region, the spacer and the −10 region. The −35 and −10 regions are underlined. **(C)** Comparison of the mean promoter activities (RLU/OD_620nm_) of P_*23*_, P_*veg*_, P_*gyrA*_ and P_*xylS2*_ after 4.5 h of growth in THY. Experiments were performed in biological triplicates and each measurement in technical duplicates.

Constitutive promoters drive the stable expression of a protein of interest over the course of growth or under changing environmental conditions. To establish a set of promoters with different strengths, we assessed the activities of the lactococcal P_*23*_ promoter, the native P_*gyrA*(*Spy*)_ promoter from *S. pyogenes*, the synthetic promoter P_*xylS2*_, and the P_*veg*_ promoter from *B. subtilis* by fusing each regulatory element (Figure 3B) to the luciferase reporter gene *ffluc* (*P. pyralis*) (Que et al., 2000; Biswas et al., 2008; Radeck et al., 2013; Xie et al., 2013). The functionality of P_*23*_ and P_*veg*_ has previously been demonstrated in *S. pyogenes* (Biswas et al., 2008; Loh and Proft, 2013). However, activity has only been measured for a few time points. The P_*xylS2*_ promoter was originally tested in combination with a XylR repressor as an inducible system for *Streptococcus mutans* (Xie et al., 2013). We did not observe a response of this promoter to xylose (data not shown), which had already been observed in a recent study exploring the activity of the P_*xylA*_ promoter in GAS (Eichenbaum et al., 1998). As the P*xylS2* promoter showed constant activity in the absence of xylose, we decided to test whether this promoter could serve as a heterologous constitutive promoter for *S. pyogenes*.

All promoters exhibited their highest activity towards the end of the exponential growth phase at around 4.5 h of growth, followed by a slow decline in the promoter signal (Figure 3A). The comparison of the promoter activities from exponential cultures (4.5 h of growth) indicated that P_*23*_ had by far the highest activity, followed by the promoters P_*veg*_ and P_*gyrA*(*Spy*)_ (Figure 3C). Interestingly, the synthetic promoter P_*xylS2*_ showed the lowest activity (Figure 3C). For all promoter constructs, except P_*xylS2*_, we noticed a slight effect on the growth, as the cultures grew marginally slower than the control without promoter (Supplementary Figure S3A).

Next, we investigated whether we could modulate the activity of P_*veg*_ by varying the spacer sequence between the −35 and −10 region of the promoter to create a set of P_*veg*_ derivatives with different levels of activity. It has been shown for *E. coli* that modifying the spacer length or changing nucleotides within the spacer sequence can reduce the promoter strength (Klein et al., 2021). In the first version of P_*veg*_ (P_*veg* (1)_)), we introduced two nucleotide changes to the 17 bp between −35 and −10 (G to A and T to G) that also affect the extended −10 box sequence (Supplementary Figure S3C). For the second derivative (P_*veg* (2)_), we extended the spacer length from 17 bp to 19 bp through the insertion of two adenines, while for the third derivative (P_*veg* (3)_), we deleted two nucleotides resulting in a spacer of 15 bp (Supplementary Figure S3C). The introduced modifications resulted in a decrease in promoter activity that was not significantly different from that of the native promoter. Changing two nucleotides (P_*veg* (1)_) and shortening the spacer sequence (P_*veg* (3)_) decreased the activity by 17.2% and 10.3%, respectively (Supplementary Figures S5B, D). The extension of the spacer sequence (P_*veg* (2)_) showed the strongest effect, lowering promoter activity by 19.4% (Supplementary Figures S3B, D).

### 3.4 Inducible regulatory elements with high inducibility and low background expression

Next, we aimed to assess the activity of different inducible promoters in *S. pyogenes*. Both of the previously used inducible elements, P_*tet*_ and P_*nisA*_, have demonstrated sub-optimal performance due to high background noise or low inducibility (Supplementary Figures S4, S5) (Eichenbaum et al., 1998; Bugrysheva et al., 2011). Inspired by the work of Sorg and colleagues, who used the native trehalose- (P_*tre*_) and zinc-inducible promoters (P_*czcD*_) found in *S. pneumoniae* (Sorg et al., 2015), we took advantage of the homologous systems from *S. pyogenes* SF370. Both promoters were placed upstream of the *ffluc* reporter gene in the replicative plasmid pSpy1C and their activity was analyzed.

**FIGURE 4:**
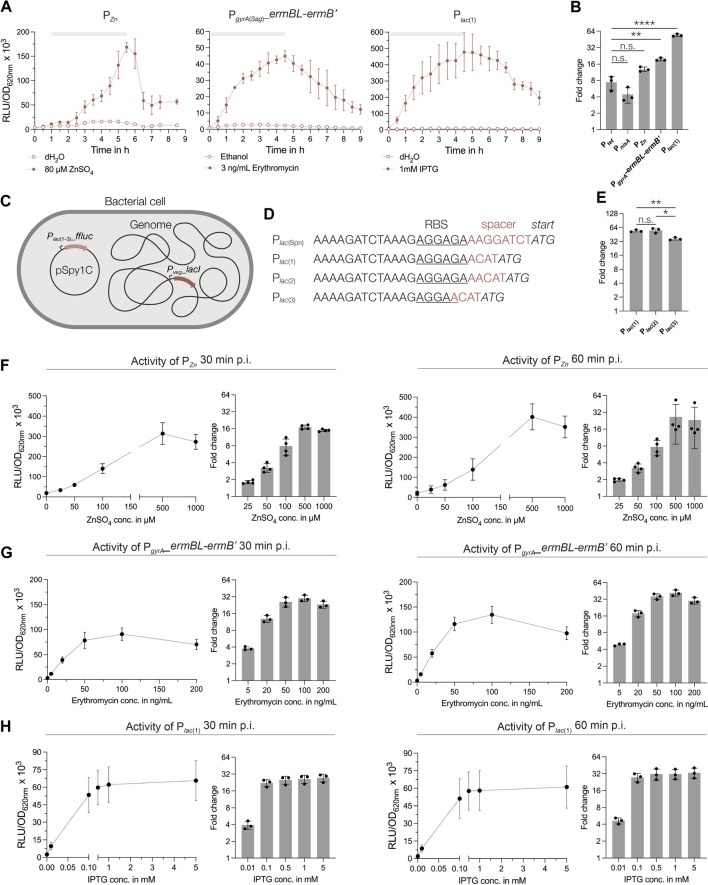
Characterization of novel inducible regulatory elements in *S. pyogenes*. **(A)** Activities of the zinc-inducible promoter, the erythromycin-inducible riboswitch and the IPTG-inducible promoter in *S. pyogenes* SF370 grown in THY medium. Promoter activities are shown in relative luminescence units normalized by optical density (RLU/OD_620nm_). Filled data points represent the signal in the presence of the respective inducer (80 µM ZnSO_4_, 3 ng/mL erythromycin and 1 mM IPTG), while empty data points show the signal in the presence of the control (distilled water or ethanol). The grey bar at the top of each graph marks the timespan of the exponential growth phase. **(B)** Comparison of the maximum fold change in signal reached during exponential growth in the presence of the respective inducers for P_*tet*_, P_*nisA*_, P_*Zn*_, P_*gyrA*(*Sag*)__*ermBL*-*ermB′* and P_*lac*_ (Ellinger, 2016). A Brown-Forsythe and Welch ANOVA test for multiple comparisons was performed to analyze statistical significance (n.s. = not significant; significance is indicated as follows: *p* < 0.05 = **, *p* < 0.0005 = ****). **(C)** Schematic drawing of the IPTG-inducible system for GAS consisting of the *lacI* repressor under control of the P_*veg*_ promoter integrated into the genome and the plasmid-based reporter harboring the P_*lac*(*Spn*)_ variants [P_*lac* (1–3)_] and the *ffluc* reporter. **(D)** DNA sequences of the modified translation initiation regions downstream of the P_*lac*(*Spn*)_ promoter [P_*lac*_ (1–3)] with changes in the spacer between RBS (underlined) and ATG (italics) highlighted in red. **(E)** Comparison of the fold changes of P_*lac*_ (1–3) after 4.5 h of growth in THY in the presence of 1 mM IPTG. A Brown-Forsythe and Welch ANOVA test for multiple comparisons was performed to analyze statistical significance [n.s. = not significant, *p* < 0.05 = significant (*), *p* < 0.005 = very significant (**)]. **(F–H)** Dose-response curves of each inducible regulatory element after induction with increasing concentrations of the respective inducer. Dose-response curves and fold changes for the respective inducer concentrations are shown for 30 min and 60 min post induction in exponential growth phase in THY medium. Experiments were performed in biological triplicates and each measurement in technical duplicates.

We did not observe an effect on growth by the inducer compounds (Supplementary Figures S4, S5). However, some of the constructs harboring the inducible promoters grew slightly slower than the controls without the promoter (Supplementary Figures S4, S5). For P_*tre*_, we did not detect an increase in promoter activity after addition of 0.4% trehalose (Supplementary Figure S4A). Instead, the signal decreased during the exponential growth phase, potentially due to a catabolite repression mechanism in the presence of glucose. In contrast, the activity of the zinc-inducible promoter increased 12.8-fold when 80 µM ZnSO_4_ were added to the culture (Figure 4A). P_*Zn*_ therefore showed a higher maximum fold change than the previously used inducible promoters P_*tet*_ and P_*nisA*_ (Figure 4B). We also tested other metal-inducible promoters, such as the promoter of the copper-specific exporter CopA (Young et al., 2015; Dao et al., 2023) and the cation-diffusion facilitator (CDF) family transporter MntE, a paralog of the zinc exporter CzcD reported to transport manganese (Rosch et al., 2009; Turner et al., 2015). We noticed only a modest induction of P_*copA*_ after the addition of 80 µM CuSO_4_ and no induction of P_*mntE*_, when 80 µM MnSO_4_ were added (Supplementary Figure S6).

Recently, a study reported the use of an IPTG-inducible expression system in *S. pneumoniae* (Liu et al., 2017). Based on this system, we created a reporter plasmid harboring the *lacI* repressor and the P_*lac*(*Spn*)_ promoter driving expression of the *ffluc* reporter (pSpy1C-PF6_*lacI*-P_*lac*(*Spn*)__*ffluc*). When we tested this construct, we observed low background expression and high inducibility upon induction with 1 mM IPTG (Supplementary Figure S4). We also noticed a growth delay caused by the construct, indicating that the expression of LacI from the high-copy plasmid pSpy1C had negative effects on streptococcal growth (Supplementary Figure S4). Thus, we optimized the construct and integrated the *lacI* repressor gene under control of P_*veg*_ into the genome using p7INT (Figure 4C; Supplementary Table S1). The LacI-expressing strain was then transformed with three *ffluc* reporter plasmids containing different modifications downstream of the P_*lac*(*Spn*)_ promoter (Sorg et al., 2015; Ellinger, 2016; Schuster and Reisch, 2021), such as changes in the length of the spacer and RBS sequence (Figure 4D). These modifications were introduced as no viable *E. coli* clones were obtained with the original P_*lac*(*Spn*)_ sequence. Genomic integration of the *lacI* gene improved the growth of the strains, although not back to wildtype level (Supplementary Figure S7C. The P_*lac*(*Spn*)_ promoter versions P_*lac* (1)_ and P_*lac* (2)_ showed very similar activity profiles over the course of growth after addition of 1 mM IPTG, with a maximum induction of 54-fold and 55.8-fold in the exponential growth phase, respectively (Figures 4A, E; Supplementary Figure S7). The P_*lac* (3)_ promoter showed a lower signal and reduced background noise, resulting in a 36.5-fold increase in signal during the exponential growth phase (Figure 4E; Supplementary Figure S7). When comparing the maximum induction levels reached by each inducible regulatory element, the promoters P_*lac* (1)_ (and P_*lac* (2)_) displayed the highest fold change overall and a significantly higher induction compared to P_*tet*_ (Figures 4B, E).

Finally, we examined the activity of an erythromycin-sensitive riboswitch in *S. pyogenes*, which was successfully applied in a recent study (Wulff et al., 2023). The P_*gyrA*(*Sag*)_ promoter from *Streptococcus agalactiae*, together with the erythromycin-responsive 5′UTR of the *ermB* gene, was placed upstream of the *ffluc* reporter in pSpy1C. Upon induction with 3 ng/mL erythromycin, the luminescence signal increased up to 19.5-fold during the exponential growth phase and showed low background expression in the absence of the inducer (Figure 4A). It is worth mentioning that erythromycin concentrations for induction should not exceed 3 ng/mL at low cell densities (e.g., OD_620nm_ = 0.02), whereas up to 50 ng/mL can be used without affecting growth when samples are induced during the exponential growth (e.g., OD_620nm_ = 0.25) (Figures 4A, G; Supplementary Figures S4, S5, S8).

Next, we selected the three regulatory elements P_*lac* (1)_, P_*Zn*_ and P_*gyrA*(*Sag*)_ *_ermBL-ermB*’ to analyze their dose-response characteristics. The reporter strains were grown to the exponential growth phase and then induced with increasing concentrations of the respective inducer. We observed a dose-dependent response of P_*Zn*_ for concentrations between 25 μM and 500 µM ZnSO_4_ (Figure 4F) already 30 min post induction. Higher concentrations, such as 1 mM ZnSO_4_, resulted in slightly inhibited growth, and thus, in a reduction in signal (Figure 4F; Supplementary Figure S8). We obtained the maximum fold induction for P_*Zn*_ using 500 µM ZnSO_4_, resulting in a 17- and 26.5-fold change of the signal at 30 min and 60 min post induction, respectively (Figure 4F). Maximum activity of P_*Zn*_ was reached at around 60 min post induction (Supplementary Figure S8).

The erythromycin-inducible riboswitch responded in a dose-dependent manner to concentrations between 5 ng/mL and 100 ng/mL of the antibiotic (Figure 4G). We observed a weak growth inhibitory effect for erythromycin concentrations above 100 ng/mL (Supplementary Figure S8). Consequently, the fold change in signal did not increase much further for concentrations above 50 ng/mL and even decreased slightly when 200 ng/mL erythromycin were supplemented (Figure 4G). Therefore, we recommend a maximum inducer concentration of 50 ng/mL for cultures in exponential growth phase, resulting in a 25.8- and 35.9-fold change in signal at 30 min and 60 min post induction, respectively (Figure 4G).

The P_*lac* (1)_ promoter showed the fastest response, with maximum induction reached already 30 min post addition of IPTG, irrespective of the inducer concentration (Supplementary Figure S8). While adding 0.01 mM IPTG triggered only a 3.9-fold increase in signal at 30 min post induction, the fold-change increased and varied only slightly when concentrations between 0.1 mM and 5 mM IPTG were applied (22.3 to 27.5-fold) (Figure 4H). These values increased only marginally after 60 min with 27.5 to 33.3-fold for concentrations ranging from 0.1 mM to 5 mM, respectively (Figure 4H). Expectedly, the addition of IPTG, even at high concentrations, had no inhibitory effect on growth (Supplementary Figure S8).

In summary, we found that P_*lac* (1)_, P_*Zn*_ and P_*gyrA*(*Sag*)_ *_ermBL-ermB*’ enable inducible gene expression in *S. pyogenes,* showing high inducibility, dose-dependency and low-to-no background expression.

### 3.5 Optimizing the application of fluorescence reporters in *S. pyogenes*

Early studies on the use of GFP in *S. pyogenes* suggested that growth conditions, such as low oxygen and acidification of the growth medium, contradict the function of FPs (Aymanns et al., 2011). Only a few studies subsequently demonstrated the use of FPs, such as mCherry or GFP, to label GAS to monitor infection progression or determine protein localization (Nerlich et al., 2009; Vega et al., 2013; Liang et al., 2019). However, the performance of newer generations of fluorescent reporters with improved features has yet to be evaluated.

When we initially attempted to analyze the performance of the fluorescent reporter mNeongreen in the plate reader, we were unable to detect a signal in THY medium. Indeed, it has been described previously for Group B Streptococci (GBS) that fluorescence measurements are difficult due to the strong autofluorescence of the growth medium (Sullivan and Ulett, 2018). Thus, to enable continuous fluorescence measurements in the plate reader, we developed a growth medium with reduced autofluorescence.

**FIGURE 5:**
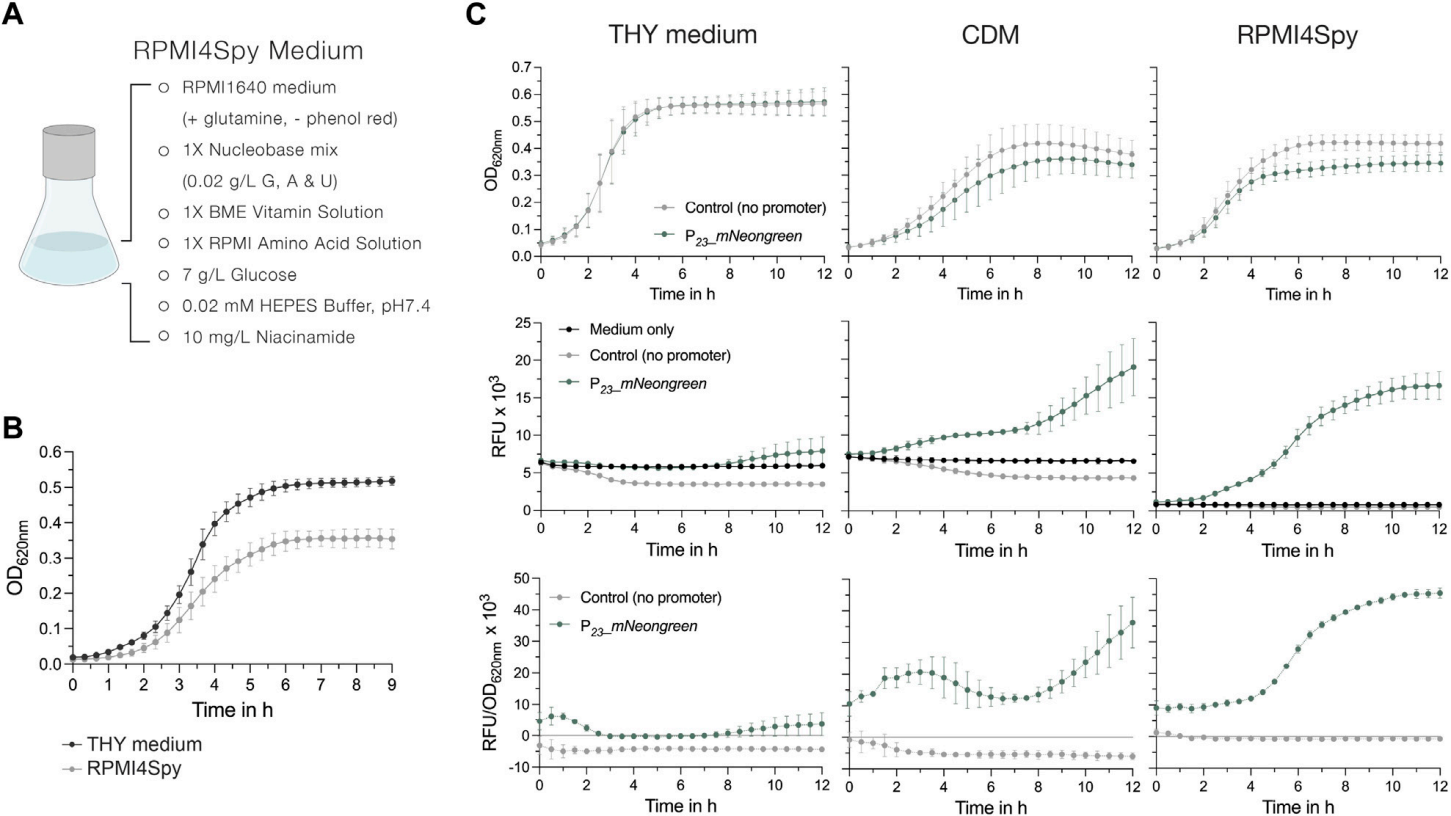
The chemically defined medium RPMI4Spy enables fluorescence measurements in plate readers. **(A)** Composition of the RPMI4Spy growth medium. RPMI1640 (with glutamine and without phenol red) forms the base of this medium. **(B)** Growth of the *S. pyogenes* SF370 wildtype shown as OD_620nm_ in THY medium compared to RPMI4Spy. **(C)** Top row: Growth (OD_620nm_) of the mNeongreen reporter strain (green) and the control strain harboring mNeongreen without any promoter in THY medium (left), chemically defined medium (CDM) (center) and the RPMI4Spy medium (right). Middle row: Relative fluorescence units (RFUs) measured for medium only (black), the mNeongreen reporter strain (green) and the control strain harboring mNeongreen without any promoter (grey) in THY medium (left), CDM (center) and the RPMI4Spy medium (right). Bottom row: Relative fluorescence units calculated and normalized by optical density (RFU/OD_620nm_) measured for the mNeongreen reporter strain (green) and the control strain harboring mNeongreen without any promoter in THY medium (left), CDM (center) and the RPMI4Spy medium (right). Experiments were performed in biological triplicates and each measurement in technical duplicates.

Earlier, Schulz and colleagues adapted the cell culture medium RPMI1640 to facilitate growth of *S*. *pneumoniae* (Schulz et al., 2014; Hirschfeld et al., 2020). RPMI1640 is available without phenol red and its colorless appearance presented a good starting point for the adaptation of a novel chemically defined medium for *S. pyogenes*. We established several formulations until *S. pyogenes* growth in this medium was comparable to that in other commonly used chemically defined medium (CDM) (Supplementary Figure S9; Supplementary Table S8). The final recipe, adapted from a formulation published while we worked on the composition (Brouwer et al., 2020), contains a Nucleobase mix, the commercially available BME vitamin and RPMI amino acid solutions, glucose and niacinamide, and is further buffered with HEPES buffer (Figure 5A). When we compared the growth of *S. pyogenes* SF370 in THY and RPMI4Spy, bacteria grew to a higher final optical density in the nutrient-rich THY medium, reaching a maximum OD_620nm_ of ∼0.5 in THY and ∼0.35 in RPMI4Spy (Figure 5B). However, the growth curve profiles appeared similar, showing neither a prolonged lag phase nor an early onset of cell death in the stationary growth phase (Figure 5B).

Next, we assessed the differences in fluorescence signals of the mNeongreen reporter strain grown in THY, CDM or RPMI4Spy. In both chemically defined media, CDM and RPMI4Spy, the mNeongreen reporter strain showed reduced growth, indicating a potential fitness burden in the defined media due to the high expression of mNeongreen compared to the control (Figure 5C). This was not observed in nutrient-rich THY medium (Figure 5C). When comparing the relative fluorescence units (RFU) of the growth medium itself with the control and reporter strain, we found that the signal of medium and reporter in THY medium overlap, creating the impression that the reporter strain is not producing mNeongreen (Figure 5C). Despite the autofluorescence of the CDM, we detected an increase in mNeongreen signal after several hours of growth (Figure 5C). We hypothesize that, in comparison to THY, CDM contains lower concentrations of autofluorescence-causing vitamins, such as riboflavin (Surre et al., 2018). Once riboflavin is taken up and used by the bacteria, the autofluorescence decreases, which is not observed in the medium control without bacteria (Figure 5C). In line with this, the signal of the control strain was lower than the medium autofluorescence, resulting in negative values when subtracting the blank and interfering with the subsequent calculation of a normalized fluorescence (Figure 5C). However, the RFU values of control strain and medium for RPMI4Spy were highly similar, and mNeongreen fluorescence was well distinguishable during growth (Figure 5C). Taken together, our results demonstrate that RPMI4Spy enables *S. pyogenes* to grow at optical densities similar to those of the chemically defined medium (CDM) previously used, and exhibits reduced autofluorescence that allows continuous fluorescence measurements in a plate reader.

**FIGURE 6:**
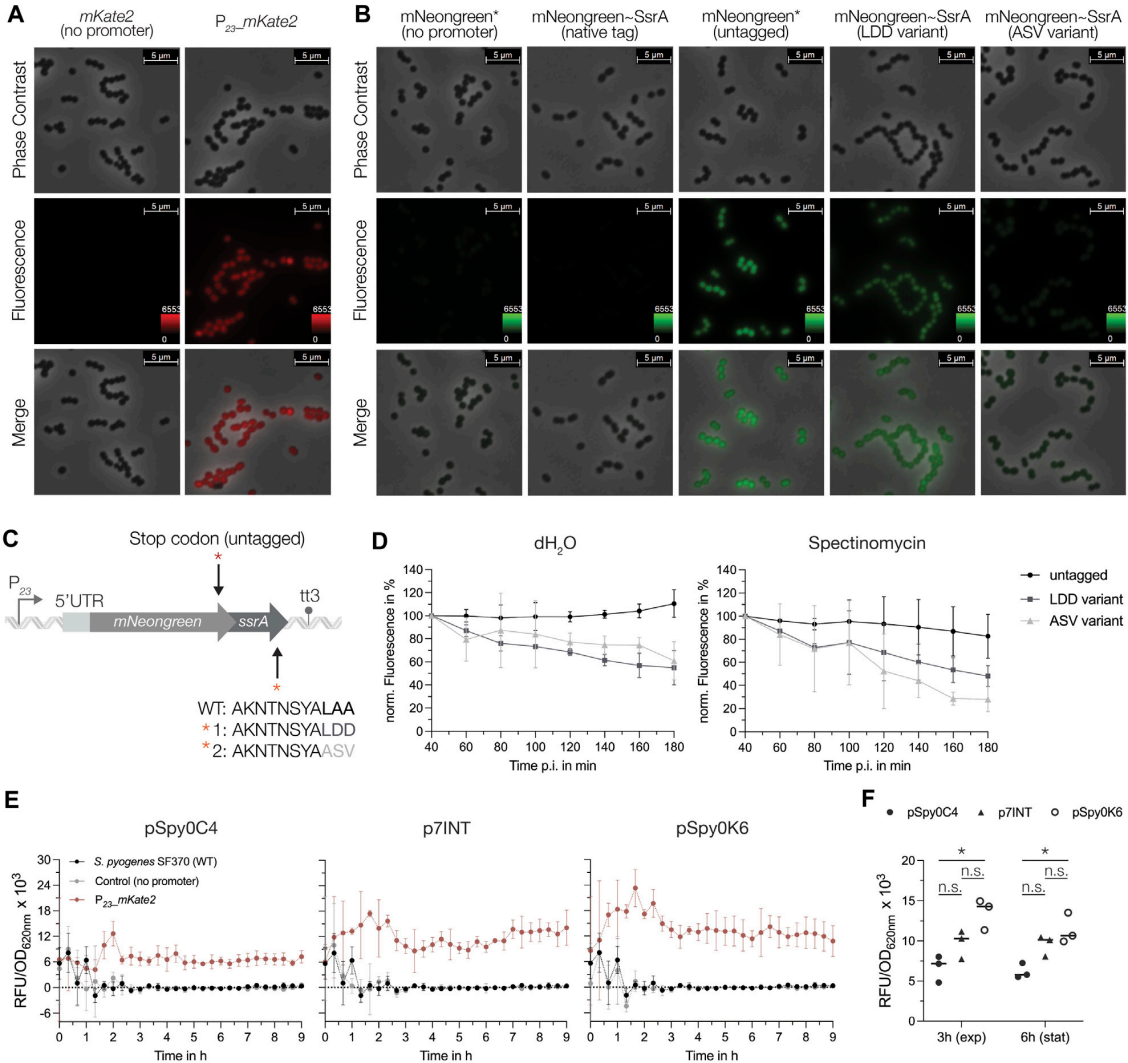
Characterization of the mNeongreen and mKate2 fluorescence reporters in *S. pyogenes*. **(A)** Fluorescence microscopy with the mKate2 reporter strain (right panel) and the respective control harboring the *mKate2* gene without the promoter (left panel). The top and middle rows show images from phase contrast and fluorescence channel, respectively. The bottom row displays a merged image of both channels. Representative images of three replicates are shown. **(B)** Fluorescence microscopy with the mNeongreen reporter strain (untagged reporter, third panel), mNeongreen fused to different SsrA degradation tag variants (native tag in the second, LDD variant tag in the fourth and ASV variant tag in the fifth panel) and the respective control strain harboring the *mNeongreen* gene without the promoter and without the SsrA tag (first panel). Top and middle rows show images from phase contrast and fluorescence channel, respectively. The bottom row displays a merged image of both channels. Representative images of three replicates are shown. **(C)** Schematic of the genetic construct design of the SsrA tag fusions to mNeongreen (native tag ending in LAA, variant 1 with an LAA to LDD mutation and variant 2 with LAA to ASV mutation) and the control harboring a stop codon (TAA) preceding the coding sequence of the *ssrA* tag. **(D)** Translation inhibition assay using spectinomycin as inhibitor to monitor the degradation of mNeongreen fused to the different SsrA tag variants over time. Left: Development of the mNeongreen signal after addition of distilled water (dH_2_O) as control. Right: Changes in mNeongreen signal after translation inhibition using 100 μg/mL Spectinomycin. **(E)** The mKate2 reporter signal (RFU/OD_620nm_) of *S. pyogenes* strains expressing the reporter from different genomic sites: pSpy0C4 (*sagB*), p7INT (*attB* (T12)) and pSpy0K6 (*SPy_1078*). The fluorescence signals are depicted as RFU/OD_620nm_ for the strain expressing mKate2 (red), the control harboring mKate2 without a promoter (grey) and the SF70 wildtype (black) for each individual integration site. **(F)** Comparison of the fluorescence signal (RFU/OD_620nm_) produced by the three different genomic integration sites in the exponential growth phase (3 h) and early stationary growth phase (6 h). Statistical significance was analyzed using a Friedman test for multiple comparisons [n.s. = not significant, *p* < 0.05 = significant (*)]. Experiments were performed in biological triplicates and each measurement in technical duplicates.

Next, we explored the use of the two codon-optimized FPs mKate2 and mNeongreen, which are characterized by enhanced stability at lower pH and increased brightness. To test their functionality in *S. pyogenes*, both reporter genes under the control of P_*23*_ and the respective control harboring each reporter gene without the promoter were integrated using p7INT (Supplementary Table S1). We then grew the strains in RPMI4Spy medium and took samples at the exponential growth phase for fluorescence microscopy. Cells expressing the mNeongreen or mKate2 reporter displayed a detectable green and red fluorescence, respectively, while the control strains without the promoter exhibited no fluorescence (Figures 6A, B). The fluorescence intensity varied slightly between individual cells (Figures 6A, B).

When using FPs as reporters for gene expression, their high stability can be disadvantageous for the detection of transient changes in transcriptional activity. To overcome this problem, unstable variants have been developed in which the FP is fused to a degradation tag, thereby taking advantage of SsrA tag-mediated proteolysis (Andersen et al., 1998; Hentschel et al., 2013). It has been shown that the last three amino acids of the SsrA tag influence the degradation rate of a protein (Andersen et al., 1998; Flynn et al., 2001). Thus, based on the native SsrA tag sequence from *S. pyogenes* (AKNTNSYALAA), we designed different SsrA tag variants that were fused to mNeongreen including (Ellinger, 2016) an untagged control with a stop codon introduced upstream of the native tag (mNeongreen*) (Schuster and Reisch, 2021), a mutant in which the last three amino acids were changed from LAA to LDD (LDD variant) and (Sorg et al., 2015) a mutant in which the last three amino acids were modified to ASV (ASV variant) to modulate stability of the reporter (Figure 6C; Supplementary Table S1). We then compared the effects of the SsrA tag variants on the fluorescence signal using microscopy. No fluorescence was observed when mNeongreen was fused to the native SsrA tag, indicating a high degradation rate of the reporter (Figure 6B). In comparison to the untagged mNeongreen (mNeongreen*), fluorescence of the FP tagged with the LDD variant appeared less bright, indicating a slight destabilization (Figure 6B). Interestingly, tagging mNeongreen with the ASV variant led to even higher destabilization, with cells emitting almost no fluorescence under the microscope (Figure 6B). To investigate this further, we determined the decrease in signal upon global inhibition of translation using spectinomycin (Figure 6D). Both, the LDD variant and the ASV variant tag resulted in a decline in fluorescence of about 52% and 72%, respectively, 180 min after the treatment (Figure 6D). For the untagged control, the signal decreased only marginally upon spectinomycin treatment (∼17% at 180 min p.i.), while the fluorescence continued to increase when distilled water was added, indicating high stability of the untagged mNeongreen protein under normal growth conditions (Figure 6D).

Finally, we assessed the effect of the genomic context on reporter gene expression. Therefore, we compared the fluorescence signals of the mKate2 reporter from three different integration sites: *sagB*, *attB* (T12) and *SPy_1078* (Figure 2B). To do so, *mKate2* was cloned into pSpy0C4, p7INT and pSpy0K6 with or without the P_*23*_ promoter, respectively (Supplementary Table S1). We then measured the fluorescence signal over the course of growth in a plate reader. Our data indicate a weak but steady expression of mKate2 from the *sagB* locus (Figure 6E). When integrated into the *attB* site of the T12 phage, the fluorescence signal appeared slightly stronger and increased gradually over time (Figure 6E). Interestingly, integration of the reporter into *SPy_1078* resulted in the highest fluorescence signal (Figure 6E). However, this signal began to decrease towards stationary growth phase (Figure 6E). The differences in signal between the integration sites became more apparent when we directly compared the mKate2 signals from the exponential (3 h) and the early stationary growth phase (6 h). Indeed, integration using pSpy0K6 resulted in the highest fluorescence signal (Figure 6F). However, this signal decreased at 6 h, whereas it remained stable when pSpy0C4 or p7INT were used for integration (Figure 6F). The growth of *S. pyogenes* did not appear to be affected by the different integrated constructs (Supplementary Figure S10). We conclude that the integration site, and thus, the surrounding genomic context may influence the heterologous expression of a gene of interest. Depending on the objective of an experiment, the genomic location of the construct must be chosen accordingly.

### 3.6 pERASE for scarless gene deletions in *S. pyogenes*

For the implementation of gene deletions in *S. pyogenes*, researchers have recently mainly relied on allelic replacement of the gene with an antibiotic resistance cassette, plasmids harboring thermosensitive replicons or the Cre-*lox* system. Particularly the last two strategies involve a time-consuming and laborious process for plasmid curing or Cre-*lox* cassette removal. Additionally, the latter procedure leaves a *lox* scar site in the genome, which in some scenarios might disrupt important regulatory sequences.

**FIGURE 7:**
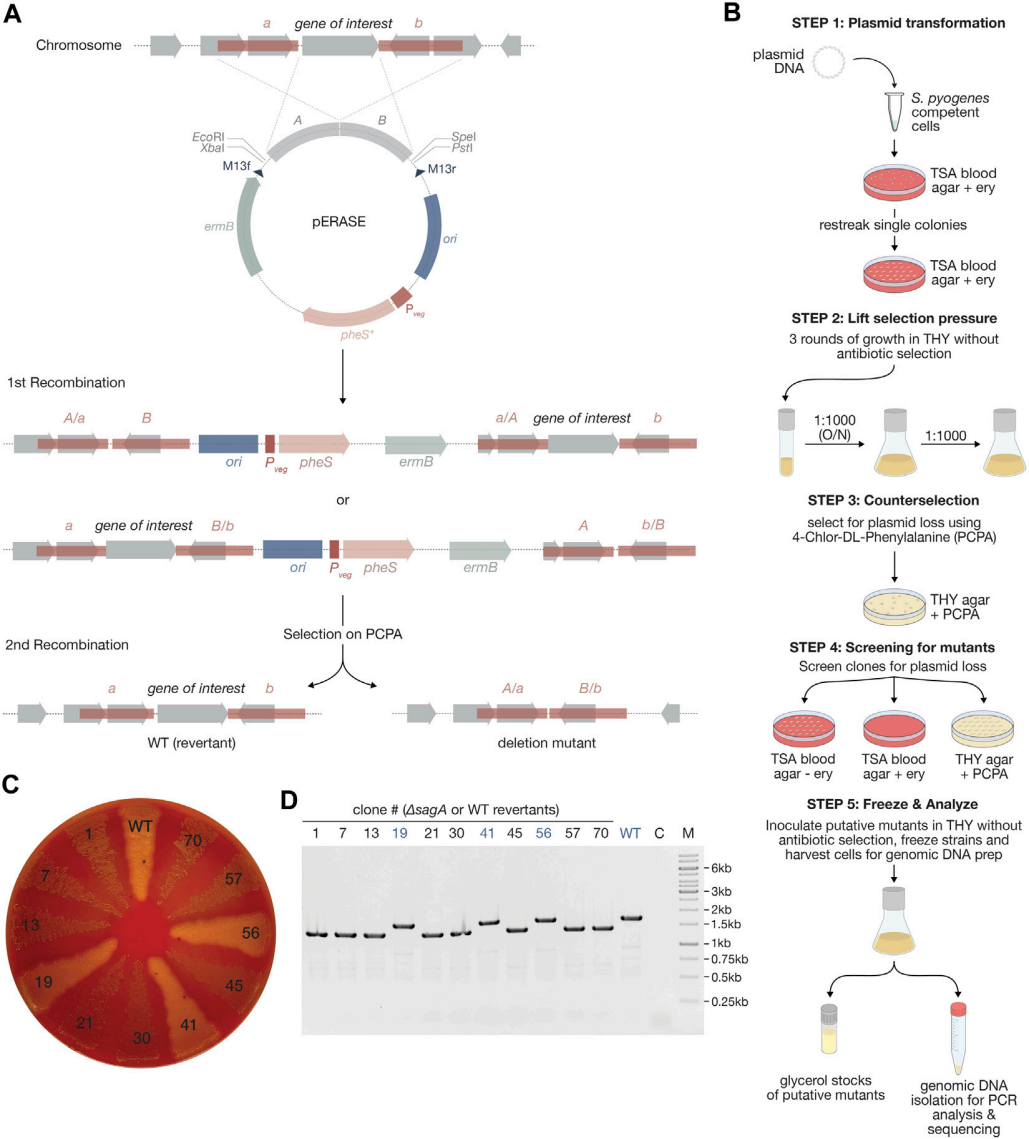
Creating scarless gene deletions using pERASE. **(A)** Schematic representation of the pERASE plasmid, subsequent recombination events after transformation into *S. pyogenes* and plasmid curing using selection on 4-chloro-phenylalanine (PCPA) agar plates. **(B)** Workflow showing the individual steps for mutant generation with pERASE in *S. pyogenes*. After transformation into *S. pyogenes*, clones with the integrated plasmid are selected on TSA blood agar with erythromycin. The plasmid is cured from the genome using three passages in liquid THY medium without antibiotic selection. Clones that have lost the plasmid are selected on THY agar plates containing 5 mM PCPA. Putative mutants growing on PCPA plates are then streaked onto TSA agar plates with and without erythromycin, as well as on THY agar plates with PCPA. For mutants with no visible phenotype, such as the loss of hemolysis, a colony PCR step can be introduced here. Several putative mutants are inoculated into THY medium to prepare glycerol stocks and isolate genomic DNA for PCR analysis. **(C)** TSA blood agar plate showing the loss of hemolysis of the putative *sagA* mutants compared to the *S. pyogenes* wildtype (WT) and wildtype revertants such as clones 19, 41 and 56. **(D)** Agarose gel showing PCR amplicons of the *sagA* region. A DNA band of ∼1.4 kb indicates the loss of *sagA*, while a band of 1.7 kb in size indicates that *sagA* is still present in the genome. WT = wildtype; C = Control without template; M = Marker (1 kb GeneRuler™ from Thermo Scientific™).

To accelerate this process and enable straightforward selection of putative scarless deletion mutants, we designed the gene editing plasmid pERASE. This plasmid features the same MCS as the other plasmids in this collection, an erythromycin resistance cassette and the high-copy origin pUC replicating in Gram-negative bacteria (Figure 7A; Supplementary Figure S1C). Moreover, the plasmid harbors the pheS* counterselection marker encoding a modified phenylalanyl-tRNA-synthetase (α-subunit) of which expression is controlled by P_*veg*_ (Figure 7A; Supplementary Figure S1C). The *pheS* gene sequence was obtained from the *S. pyogenes* genome and adapted by introducing silent mutations to the *pheS* coding sequence to prevent recombination events at the native streptococcal *pheS* locus. Moreover, two mutations were inserted to create a pheS* variant which, when expressed, incorporates the toxic halogenated analogue of phenylalanine (4-chloro-phenylalanine, short PCPA or 4CP) into proteins during translation, leading to cell death. Selection on agar plates containing PCPA therefore only allows the growth of mutants that have lost the plasmid (Figure 7A). As PCPA itself is toxic at a certain concentration, we performed an initial test experiment using TSA agar plates containing various concentrations of PCPA and found that 0.6% PCPA (30 mM) resulted in growth inhibition of the *S. pyogenes* SF370 wildtype (data not shown). Thus, a lower concentration (5 mM) was selected to ensure that only bacteria still harboring the plasmid in the genome had their growth suppressed, while deletion mutants and wildtype revertants could grow.

To test the applicability of pERASE for generating scarless gene deletions in *S. pyogenes*, we aimed to delete the *sagA* gene encoding the toxin streptolysin S. Deletion of this gene results in a non-hemolytic phenotype on TSA blood plates compared to the hemolytic wildtype strain. To this end, we cloned pERASE-*ΔsagA*, containing homologous fragments upstream and downstream of *sagA*. After transformation of the plasmid into *S. pyogenes* SF370, we selected clones that had undergone homologous recombination and integrated the plasmid on TSA blood plates containing erythromycin (Figure 7B). After one passage on selective TSA blood plates, we inoculated single clones into THY medium without antibiotics to allow for a second recombination event and subsequent loss of the plasmid (Figure 7B). We performed three passages in liquid culture before selecting for plasmid loss on THY agar plates containing 5 mM PCPA (Figure 7B). Several clones (*n* = 70) growing in the presence of PCPA were streaked onto TSA blood plates with and without erythromycin and onto THY agar plates with 5 mM PCPA (Figure 7B). Putative deletion mutants and wildtype revertants were expected to grow on TSA blood plates without antibiotics and on the PCPA plates, but not on TSA blood plates with erythromycin. We identified 17 out of the 70 clones (∼24%) that exhibited a loss of hemolysis on TSA blood plates. From these 17 clones, we selected eight showing no hemolysis, while from the remaining 53 clones displaying a hemolytic phenotype, we selected three as controls (potential wildtype revertants) for PCR analysis (Figures 7C, D). The absence of *sagA* in these eight clones was proven by PCR and Sanger sequencing of the three final clones. The results were consistent with the observed non-hemolytic phenotype (Figures 7C, D). This demonstrates that pERASE can indeed be applied to generate clean deletion mutants within just 8 days as compared to protocols using thermosensitive shuttle vectors where mutant generation takes about 2 weeks (Boukthir et al., 2023).

## 4 Discussion

### 4.1 *E. coli*-*S. pyogenes* shuttle plasmids for rapid cloning and heterologous gene expression

In this study, we generated a novel set of low- and high-copy replicative plasmids and demonstrated applicability of pSpy1C for the creation of reporter plasmids and the subsequent assessment of promoter activities in GAS.

It is noteworthy that RCR derivatives, such as pSH71 and pWV01*, are considered less stable when harboring inserts larger than 8 kb, whereas theta replicons like pAMβ1 show higher stability (De Vos et al., 1994). Depending on the construct of interest to be designed, the corresponding plasmid backbone must be selected thoughtfully.

When *S. pyogenes* was transformed with the empty plasmids*,* we found that the cultures displayed varying growth profiles. One reason for a plasmid-caused growth delay could be a divergent directionality of genes encoded on the plasmid, leading to interference of replication and transcription processes (Mirkin and Mirkin, 2005; Srivatsan et al., 2010). However, this did not seem to be the case as all open reading frames encoded on, e.g., pSpy2C show the same directionality. We suspect this observation to be an effect of the plasmid copy number (PCN) and, consequently, a gene dosage effect of the antibiotic resistance-conferring enzyme encoded on each plasmid, given that growth profiles varied between antibiotic markers. It has been shown that higher PCNs are associated with increased resistance levels against antibiotics (San Millan et al., 2015). Consistent with this, we observed a faster generation time of *S. pyogenes* harboring the low-copy plasmid pSpy2C and pSpy3C when the chloramphenicol concentration was decreased, while still ensuring plasmid maintenance. Further adaptation of the selective concentrations of kanamycin and erythromycin could help to optimize experimental growth conditions for the remaining plasmids of the collection.

### 4.2 Integrative plasmids allow for the stable and site-specific integration of genes of interest into the streptococcal genome

To enable the stable integration of genetic elements into the genome and thereby avoid PCN effects on the expression of a gene of interest, integrative plasmids have been widely applied in various bacterial model organisms (Radeck et al., 2013; Keller et al., 2019). To our knowledge, only two site-specific integrative plasmids for use in GAS have been described: pFW11e integrating into a gene coding for a sugar phosphate isomerase (*SPy_0535*) and p7INT inserting at the 3′end of the tmRNA locus (Podbielski et al., 1996; McShan et al., 1998; Loh and Proft, 2013). Compared to integrative plasmids previously used, pSpy0C2 and pSpy0C4 allow direct selection of integration events on starch and blood plates, respectively. It is worth noting that both genes, *amyA* and *sagB,* are involved in streptococcal virulence, which needs to be taken into account when performing infection experiments (Shelburne et al., 2009; Molloy et al., 2011). As an alternative, we designed pSpy0K6 integrating into a transcriptionally silent locus, offering two main advantages (Ellinger, 2016): the integrated construct is not affected by read-through from strongly transcribed surrounding genomic regions (Sauer et al., 2016; Schuster and Reisch, 2021) the integration does not disrupt a functional gene required for growth or virulence.

The integration sites for pSpy0C2, pSpy0C4 and pSpy0K6 are highly conserved between SF370 and the more clinically relevant M1T1 5,448 strain. We also found GAS strains of other serotypes, such as M28 MGAS6180 or M12 MGAS9429, to have high DNA sequence identity with the integration sites, potentially expanding the host range of these plasmids to other streptococcal strains. Shortening the plasmid-encoded homologous regions required for recombination could result in higher sequence homology with other serotypes, and thus, a broader host range. Previous research suggests the use of homologous regions between 250 and 500 bp in length, although longer sequences typically achieve higher recombination efficiencies (Cho et al., 2019). Because of the different resistance cassettes, these plasmids can also be used in combination, enabling more advanced genetic modifications. Finally, the provided list of transcriptionally silent sites found in SF370 and M1T1 5,448 will enable other research groups to design further site-specific integrative plasmids.

### 4.3 Novel promoters for controlled gene expression in *S. pyogenes*

In this study, we assessed the activity of different constitutive promoters and tested potential novel inducible systems for tunable gene expression in GAS. Our side-by-side comparison of different constitutive promoters highlighted P_*23*_ and P_*veg*_ as the strongest promoters, while P_*gyrA*_ and P_*xylS2*_ showed weaker activities, covering a wide range of expression levels that researchers can select from. While strong promoters are advantageous when studying the effect of gene overexpression on the cell or to obtain higher yields in protein purification procedures, low expression may be preferred when studying toxic proteins or poorly soluble proteins (Duzenli et al., 2020). To further increase the variety in promoter strengths, we modified the promoter P_*veg*_ by mutating the spacer sequence between the −35 and −10 region. Although we observed a decrease in promoter activity, the modifications did not result in a significant reduction of the promoter strength. Consistent with these results, a recent study demonstrated for a set of *E. coli σ* ^70^-promoters that changes in spacer length from 15 bp to 19 bp have no significant impact on promoter strength, whereas longer spacers (>19 bp) result in significantly lower expression levels (Klein et al., 2021).

As the number of inducible regulatory elements for *S. pyogenes* is very limited, we sought to evaluate different inducible promoters for use in GAS. The two commonly used inducible promoters, P_*tet*_ and P_*nisA*_, showed high leakage and low inducibility in our experiments, respectively. In contrast, the three novel inducible regulatory systems described here outperformed the previous systems, demonstrating high inducibility, no-to-low background expression and a dose-dependent response to different inducer concentrations. The P_*lac*_ promoter performed best when the LacI repressor was constitutively expressed from the streptococcal genome, consistent with previous observations made in *S. pneumoniae* (Keller et al., 2019). We hypothesize that high LacI expression levels from replicative plasmids could result in unspecific binding of the repressor to DNA sequences with high similarity to LacI operator sites, potentially affecting the expression of genes involved in metabolism, and consequently, growth. Nevertheless, we observed a slight influence on growth for the strain expressing LacI from the chromosome, independently of the presence of the reporter plasmid. Potentially, replacing P_*veg*_ driving *lacI* transcription with a weaker promoter, e.g., P_*xylS2*_, could resolve the minor growth defect. However, the reduced expression of the LacI repressor may result in a higher background noise of the promoter when used in combination with a high-copy reporter plasmid.

Compared to, e.g., P_*lac* (1)_, our data demonstrated a high tunability of P_*Zn*_ induction levels, making this promoter interesting for the study of dose-dependent effects. It is noteworthy that a tight control of metal ion homeostasis is critical for growth and survival of GAS in the human host, and thus, inducer concentrations need to be selected appropriately (Ong et al., 2018).

RNA-based regulatory systems, such as riboswitches, are usually highly conserved between bacteria in comparison to protein-based inducible expression systems and, consequently, are better transferrable (Barrick and Breaker, 2007). Here, we have successfully demonstrated the applicability of an erythromycin-inducible riboswitch in *S. pyogenes*. We like to highlight that this system is not the only RNA-based regulatory system applicable to GAS, as another study reported a theophylline-sensitive synthetic riboswitch for use in *S. pyogenes,* showing high inducibility and very low basal expression in the absence of the inducer (Topp et al., 2010).

Attempts to establish the trehalose-inducible promoter (P_*tre*_) did not prove successful, probably due to carbon catabolite repression. We hypothesize that glucose utilization by GAS led to downregulation of this promoter during exponential growth. Previous research suggests that the trehalose operon is repressed by the catabolite control protein A (CcpA), despite a direct binding by CcpA could not be proven (DebRoy et al., 2021). Potentially, induction could be observed in a growth medium devoid of glucose or fructose, which were shown to be the preferred carbon sources for GAS (Ribardo and McIver, 2006).

For all luciferase reporter strains, we observed a decrease in luminescence towards the stationary growth phase. It was proposed that the luminescence decline observed in the transition and stationary growth phase could result from the associated decrease in intracellular ATP levels, subsequently leading to less availability of ATP for the conversion of the substrate D-luciferin to oxyluciferin and light (Figure 3A) (Wiles et al., 2005; Loh and Proft, 2014). When stationary phase cultures are being studied, ATP-independent reporters, such as the luciferases from *Renilla reniformis* or *Gaussia princeps* could be applied (Wu et al., 2015).

### 4.4 mNeongreen and mKate2 as reporters for gene expression in GAS

Although a wide range of luciferases exists, these reporters are limited in their application as they, e.g., do not support studying protein localization. To provide alternative reporters for *S. pyogenes*, we aimed to assess the functionality of fluorescent reporters of newer generations with improved properties, such as increased brightness and higher stability at acidic pH.

Interestingly, although GFP and its derivatives have been widely used in many other Gram-negative and Gram-positive bacteria, the number of studies using fluorescence reporters in GAS is very limited (Nerlich et al., 2009; Aymanns et al., 2011; Liang et al., 2019). Here, we demonstrated that new generations of fluorescence proteins, such as mNeongreen and mKate2, can indeed be applied in GAS and, in combination with the new genetic toolset, could serve to understand protein localization, gene expression or protein-protein interactions.

Previous research already highlighted difficulties in measuring fluorescence signals in Group B Streptococci due to growth medium autofluorescence (Sullivan and Ulett, 2018). The causes of this autofluorescence are vitamin components in the medium, such as riboflavin that is mainly responsible for green autofluorescence (Surre et al., 2018). One possibility to circumvent this problem is to wash and resuspend the cells in 1x PBS buffer to measure fluorescence. However, the required washing step is time-consuming and results in deviations from the original optical density of the culture. The RPMI4Spy medium was adapted from a previously published recipe in which growth of the GAS clinical isolate HKU16 was demonstrated (Brouwer et al., 2020). The greatly decreased autofluorescence of RPMI4Spy enabled the monitoring of fluorescence signals from bacteria expressing mNeongreen or mKate2 over the course of growth, indicating no decrease in fluorescence signal when cells transitioned to the stationary phase. Finally, RPMI4Spy opens up the use of methodologies that have previously been only rarely applied on GAS, such as time-lapse fluorescence microscopy (O Neill et al., 2016).

When using fluorescent proteins as a readout for gene expression, their high stability can constitute a disadvantage as transient changes in gene expression, particularly downregulation, may not be accurately represented. Therefore, we tested the modulation of mNeongreen protein stability by adding different SsrA degradation tag variants. We found that mutating the last three amino acids of the native SsrA tag sequence altered mNeongreen stability to varying degrees. Although we barely detected a fluorescence signal for the ASV variant, the signal of the LDD variant differed only slightly from the untagged reporter. Our data on the ASV variant mainly reflect results from previous studies in *Mycobacterium* and *E. coli* showing a similar degree of destabilization, however, the overall fluorescence intensity of this variant is strongly decreased in *S. pyogenes* (Andersen et al., 1998; Blokpoel et al., 2003; Allen et al., 2007; Hentschel et al., 2013). This indicates that many factors play a role in the design of such degradation tags, including the complete SsrA tag sequence, the microorganism used as chassis, and thus, the present degradation machinery (Flynn et al., 2001). No signal was detected from mNeongreen fused to the native tag, which is also in agreement with previous research (Hentschel et al., 2013).

When we assessed the signal of the mKate2 reporter from different genomic locations, we noticed that integrations into *SPy_1078* using pSpy0K6 resulted in the highest signals. It has been shown that the proximity of a heterologous gene to the origin of replication influences its expression levels due to gene dosage effects during replication (Block et al., 2012; Sauer et al., 2016). In this case, all integration sites are located quite far from the origin of replication, and thus, expression is expected to be lower. The replication terminus of *S. pyogenes* SF370 is not well characterized, but a *dif*-like termination sequence identical to that in *E. coli* was identified, starting at position 929,320 (Ferretti et al., 2001). Interestingly, of the three integration sites evaluated, *SPy_1078* is the closest to the terminus and should, according to previous studies, demonstrate the lowest expression. As this was not the case, gene dosage cannot be the explanation here. A recent study was similarly unable to correlate gene dosage effects with expression levels of a fluorescent reporter expressed from different genomic regions in *E. coli* (Bryant et al., 2014). The authors found that, although the same promoter was placed in front of the reporter, the RNA polymerase occupancy varied between the different genomic sites resulting in different fluorescence intensities (Bryant et al., 2014). In line with this, recent findings showed that transcriptional regulation in bacteria is also mediated by chromosome re-modelling via nucleoid associated proteins (NAPs), making genes accessible or inaccessible to transcription depending on different growth conditions, such as stress (Rashid et al., 2023). The changes in mKate2 fluorescence observed between the exponential and early stationary phase could result from such chromatin re-modelling. Consequently, a variety of parameters can influence the expression of a heterologous gene from a specific genomic locus.

It remains to be investigated whether alternative reporters, e.g., fluorescent RNA aptamers such as Spinach or Mango, may also be applied in GAS (Paige et al., 2011; Dolgosheina et al., 2014; Ouellet, 2016). Such systems could be advantageous for the study of bacterial transcription, as the signal is directly proportional to RNA levels.

### 4.5 pERASE facilitates gene editing in GAS

Several studies showed the applicability of the pheS* counterselection marker for genome editing in various bacterial species (Kristich et al., 2007; Xie et al., 2011; Schuster et al., 2019; Gao et al., 2022). According to a recent review, pheS* has already been successfully applied in the genetic manipulation of *S. pyogenes* by Caparon and Port (unpublished) (Cho et al., 2019). However, genetic engineering in GAS currently still relies on plasmids harboring thermosensitive origins, transposon-based systems or the Cre-*lox* strategy (Le Breton and McIver, 2013; Le Rhun et al., 2017; Cho et al., 2019).

Unlike thermosensitive shuttle vectors, such as the recently published pBFK, pERASE offers several advantages, such as its minimal size, no need for temperature shifts, a counterselection marker to screen for plasmid loss and the possibility of red-white screening during cloning in *E. coli* (Boukthir et al., 2023). Particularly the use of a counterselection marker reduces the workflow for mutant generation to 8 days as demonstrated for the clean deletion of *sagA*. We believe that this time period can be further shortened by reducing the number of rounds for plasmid curing in liquid THY or by adding PCPA already at this stage. The latter approach was previously shown for counterselection after transformation into *S. pneumoniae* and the protocol could therefore be further optimized (Sorg et al., 2020). Moreover, we believe that pERASE can be applied not only for gene deletions but also for the introduction of point mutations, small tags or the insertion of genes.

## Data Availability

The datasets presented in this study can be found in online repositories. The names of the repository/repositories and accession number(s) can be found below: https://www.ebi.ac.uk/ena, PRJEB72852.
