# Supplementary material for: Expanding the genetic toolbox for the obligate human pathogen Streptococcus pyogenes
Source: Front Bioeng Biotechnol. 2024 Jun 7;12:1395659. doi: 10.3389/fbioe.2024.1395659 (PMC11190166; doi:10.3389/fbioe.2024.1395659)
Supplement: Supplementary file 1 [file DataSheet1.pdf]

Supplementary Material

Expanding the genetic toolbox for the obligate human pathogen *Streptococcus pyogenes*

Nina Lautenschläger[1], Katja Schmidt[1], Carolin Schiffer[1], Thomas F. Wulff[1], Karin Hahnke[1], Knut Finstermeier[1], Moïse Mansour[1], Alexander Elsholz[1] and Emmanuelle Charpentier*[1,2]

[1]Max Planck Unit for the Science of Pathogens, Charitéplatz 1, 10117 Berlin, Germany
[2]Institut für Biologie, Humboldt-Universität zu Berlin, Philippstraße 13, 10115 Berlin, Germany

*** Correspondence:**
Prof. Dr. Emmanuelle Charpentier
research@emmanuelle-charpentier.org

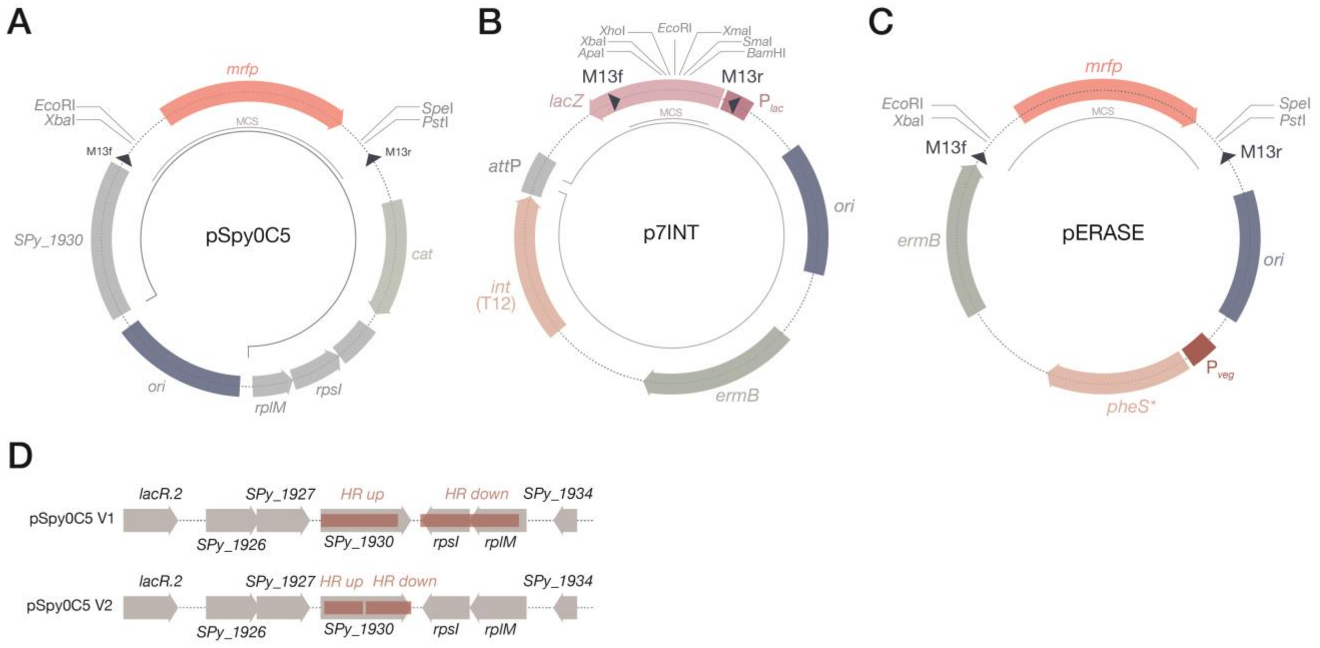

Supplementary Figure 1. Plasmid maps of the integrative plasmids pSpy0C5, p7INT and the genome editing plasmid pERASE. **(A)** Map of the pSpy0C5 version 1 plasmid. The pUC replicon for propagation in *E. coli* is marked in blue. The resistance cassette for chloramphenicol (*cat*) is highlighted in sage green. For pSpy0C5, DNA sequences with homology to the genome of SF370 are shown in grey. Restriction sites of the MCS flank the *mrfp* reporter gene under control of $P_{lac(Eco)}$, enabling red-white screening of clones containing the desired insert in *E. coli*. Small dark blue triangles indicate the binding sites for the M13f and M13r standard primers. The inner black circular line marks the part of the plasmid that will integrate into the streptococcal genome. **(B)** Plasmid map of the integrase-based plasmid p7INT. The MCS features a *lacZ* reporter gene, highlighted in pink, allowing for blue-white screening in *E. coli*. The integrase gene *int* and the respective *attP* site derived from bacteriophage T12 are highlighted in pale pink and grey, respectively. The pUC replicon for propagation in *E. coli* is marked in blue. The resistance cassette for erythromycin (*ermB*) is highlighted in sage green. The inner black circular line marks the part of the plasmid that will integrate into the streptococcal genome. **(C)** Map of the pERASE plasmid used to generate scarless gene deletions in *S. pyogenes*. pERASE contains the *mrfp* reporter for red-white screening in *E. coli* (salmon red) and the *pheS** counterselection marker (pale pink) allowing to screen for plasmid loss, and thus, deletion of the gene on 5 mM PCPA (4-Chlor-DL-Phenylalanine) agar plates. **(D)** Genomic context of the locus in the *S. pyogenes* SF370 genome targeted for integration by pSpy0C5 V1 (as in Supplementary Figure A) and V2. Sites selected to enable homologous recombination of pSpy0C5 are highlighted in red. All attempts to transform both versions of the plasmid were unsuccessful, potentially due to the stabilizing properties of the putative toxin-antitoxin locus upstream (*SPy_1926-SPy_1927*).

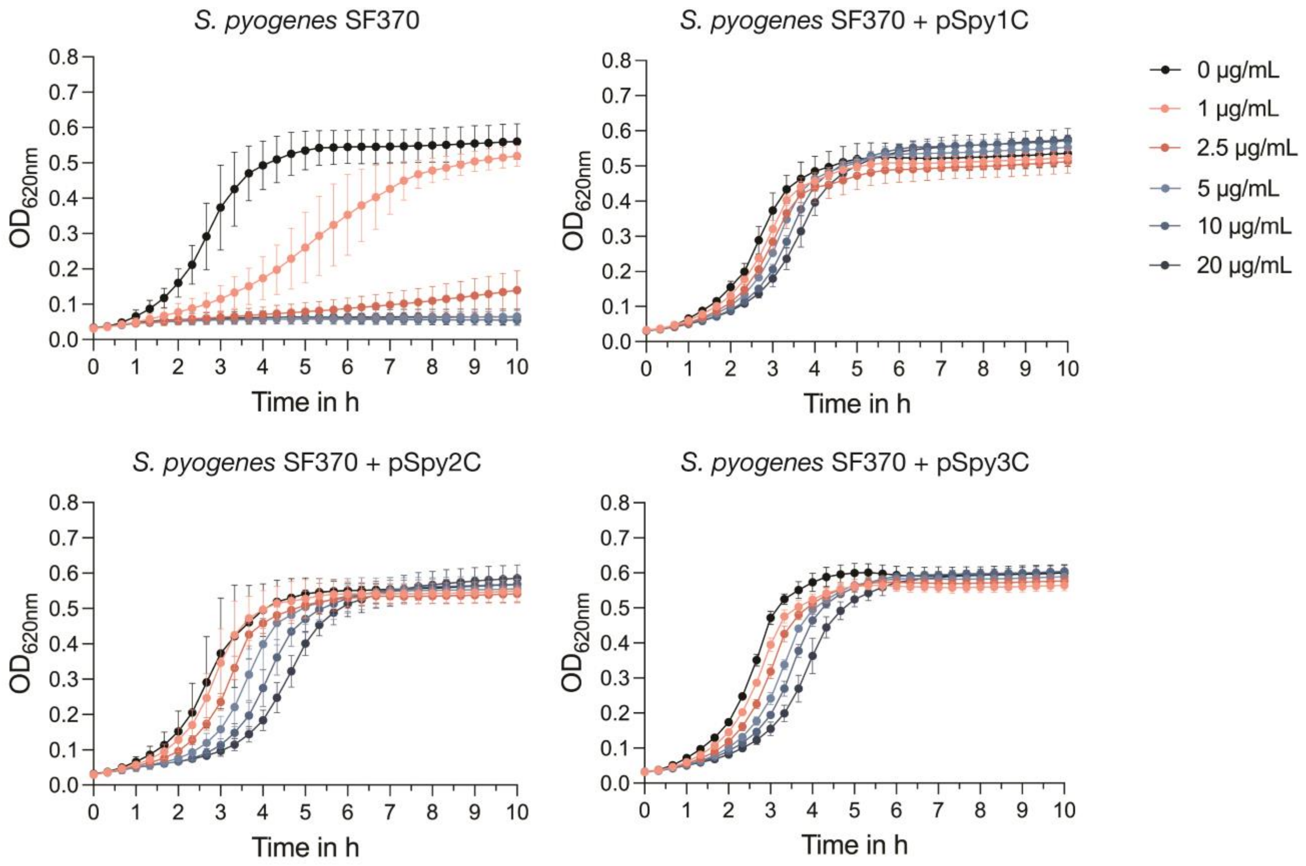

Supplementary Figure 2. Adjustments of chloramphenicol concentrations for the maintenance of replicative plasmids in *S. pyogenes* (EC2514) in THY medium. Growth in OD_{620nm} is shown for the *S. pyogenes* SF370 wildtype strain (top left) and the wildtype strain transformed with the replicative plasmids pSpy1C (top right), pSpy2C (bottom left) and pSpy3C (bottom right). Chloramphenicol was added to the cultures at the beginning of growth (t=0) at different concentrations (1 µg/mL, 2.5 µg/mL, 5 µg/mL, 10 µg/mL and 20 µg/mL), indicated by the legend at the top right. Ethanol served as a control (0 µg/mL). Experiments were performed in biological triplicates with technical replicates each.

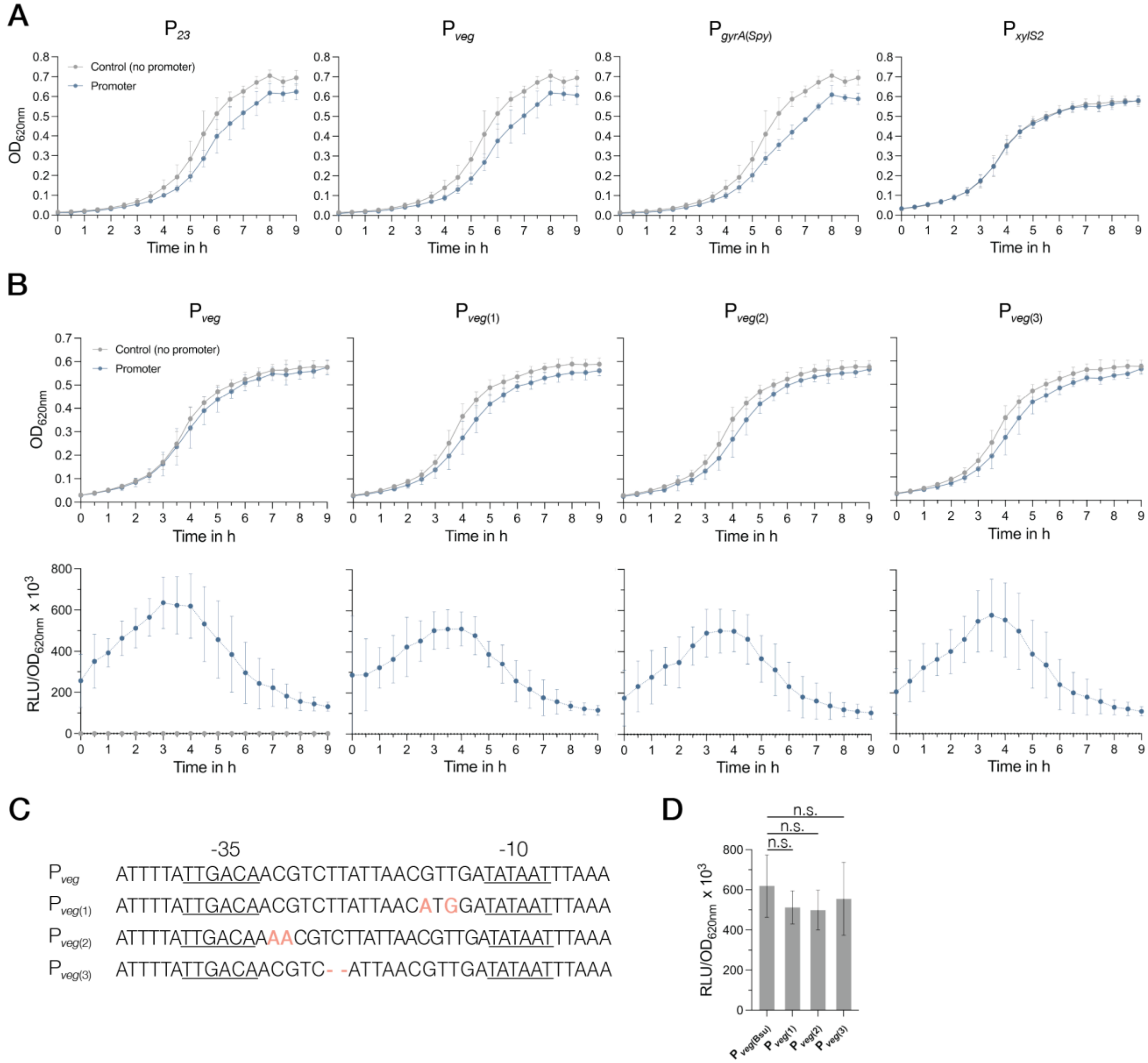

Supplementary Figure 3. *S. pyogenes* growth (solid lines) and luminescence (dotted lines) signal of the luciferase reporter strains harboring different constitutive promoters (blue) in comparison to the control without promoter (grey). **(A)** Growth (shown in OD_{620nm}) of *S. pyogenes* strains harboring either the control plasmid (pSpy1C-*ffluc*) or pSpy1C featuring the fusions of different constitutive promoters to the luciferase reporter *ffluc* (e.g. pSpy1C-P_{23}*_ffluc*). **(B)** Growth, shown in OD_{620nm} (top panel), and promoter activities, shown in relative luminescence units normalized by optical density (RLU/OD_{620nm}) (bottom panel) of strains harboring luciferase reporter plasmids with different variants of the P_{veg} promoter (1-3). **(C)** DNA sequences of the native P_{veg} promoter and its derivatives (1-3) including the -35 region, the spacer and the -10 region. The respective changes to the spacer sequence to obtain $P_{veg(1)}$ (G-to-A and T-to-G mutation), $P_{veg(2)}$ (insertion of two adenines) and $P_{veg(3)}$ (deletion of two thymines) are highlighted in apricot. **(D)** Comparison of the mean promoter activities (RLU/OD_{620nm}) of the native P_{veg} promoter and its derivatives $P_{veg(1)}$, $P_{veg(2)}$, and $P_{veg(3)}$ after 4 hours of growth in THY. Statistical significance was analyzed using a Brown-Forsythe and Welch ANOVA test for multiple comparisons (n.s. = not significant). Experiments were performed in biological triplicates and measurements in technical duplicates.

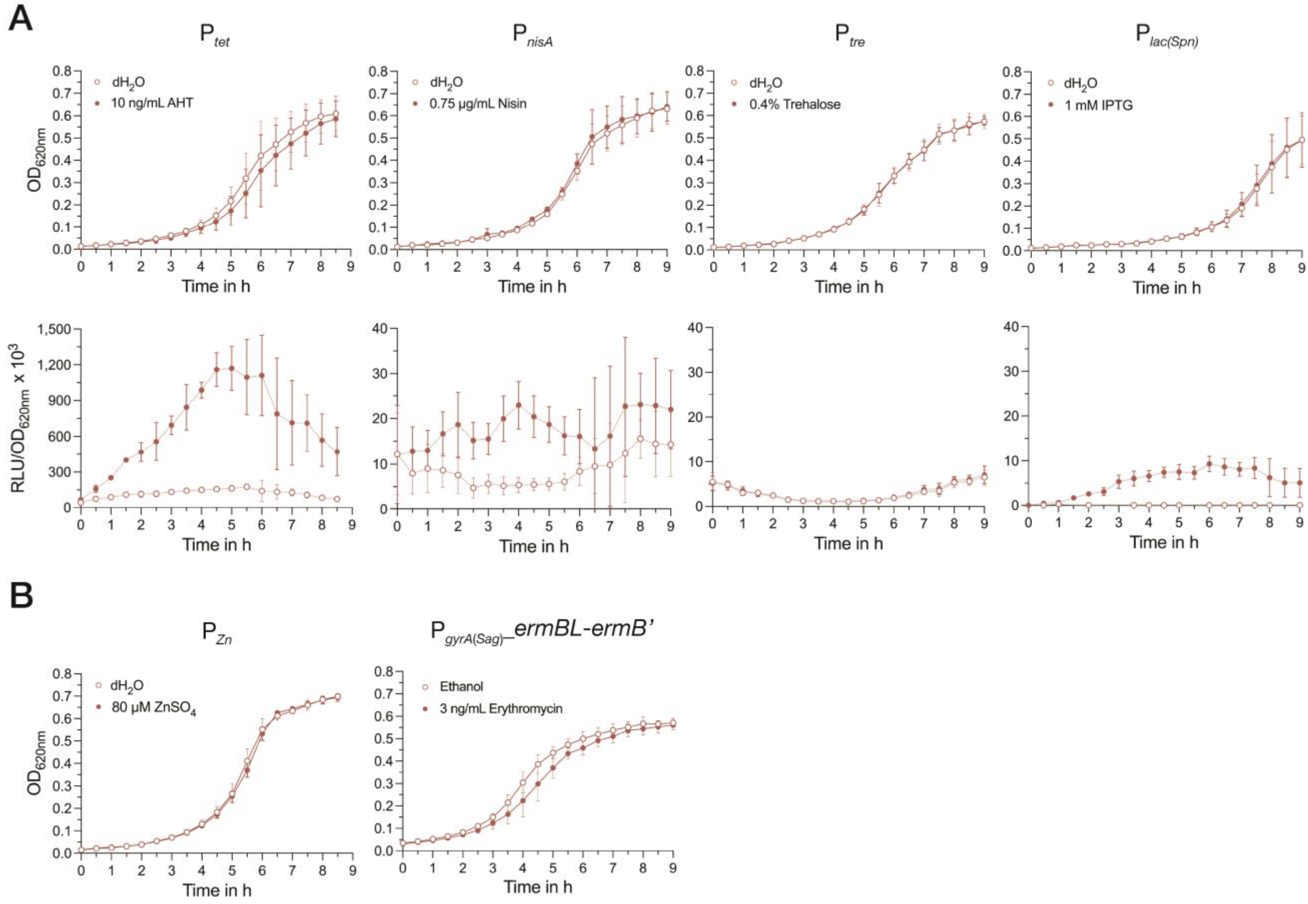

Supplementary Figure 4. Growth (solid lines) and luminescence signal (dotted lines) of the *S. pyogenes* reporter strains featuring different inducible promoters. **(A)** Growth of the reporter plasmids harboring the inducible promoter systems P_{tet}, P_{nisA}, $P_{lac(Spn)}$ and P_{tre} is shown in OD_{620nm} over time in the top row, while the luminescence signal is shown in relative luminescence units normalized by optical density (RLU/OD_{620nm}) in the bottom row. Cultures were induced with the respective inducer compounds or the control at the beginning of growth (t=0) (P_{tet}: 10 ng/mL anhydrotetracycline, P_{nisA}: 0.75 µg/mL nisin, P_{tre}: 0.4% trehalose, $P_{lac(Spn)}$: 1 mM IPTG, distilled water served as negative control). Data are shown for the uninduced condition (data points with white fill) and the induced condition (data points with red fill). **(B)** Growth of the strains harboring reporter plasmids with the inducible regulatory systems P_{Zn} and $P_{gyrA(Sag)}$_*ermBL-ermB'* shown as OD_{620nm} over time in the absence or presence of the respective inducer compounds (P_{Zn}: 80 µM $ZnSO_4$ and $P_{gyrA(Sag)}$_*ermBL-ermB'*: 3 ng/mL erythromycin). Data are shown for the uninduced condition (data points with white fill) and the induced condition (data points with red fill). Distilled water served as a negative control for P_{Zn} induction and ethanol was used as a negative control for $P_{gyrA(Sag)}$_*ermBL-ermB'*. Experiments were performed in biological triplicates and measurements in technical duplicates.

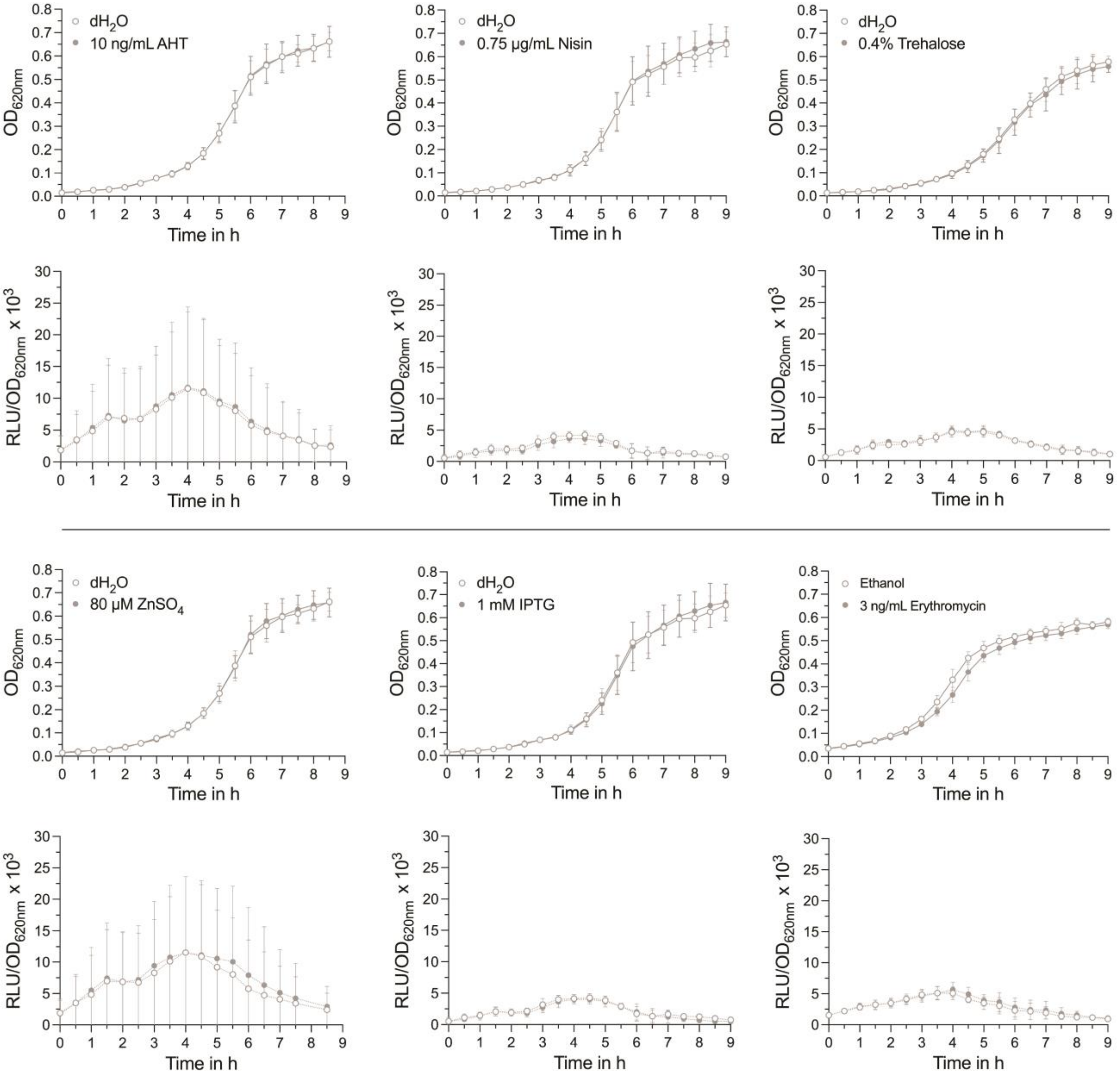

Supplementary Figure 5. Growth (solid lines) and luminescence signal (dotted lines) of the *S. pyogenes* control strains harboring pSpy1C-*ffluc* (no promoter) in the presence (data points with grey fill) and absence (data points with white fill) of different inducer compounds (P_{tet}: 10 ng/mL anhydrotetracycline, P_{nisA}: 0.75 µg/mL nisin, P_{tre}: 0.4% trehalose, P_{Zn}: 80 µM $ZnSO_4$, $P_{lac(Spn)}$: 1 mM IPTG, $P_{gyrA(Sag)}$*_ermBL-ermB'*: 3 ng/mL erythromycin). Distilled water (dH_2O) or ethanol were used as negative controls for induction. Growth is shown in OD_{620nm} over time in the first and third row. The luminescence signal is shown in relative luminescence units normalized by optical density (RLU/OD_{620nm}) in the second and fourth row. Experiments were performed in biological triplicates and measurements in technical duplicates.

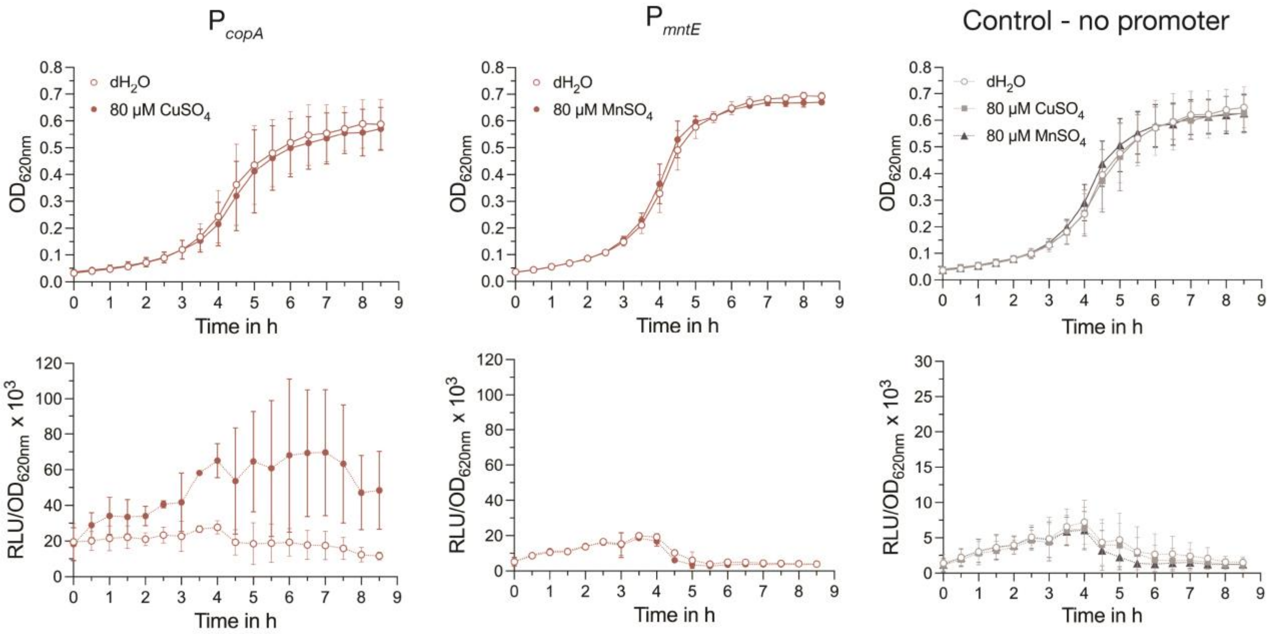

Supplementary Figure 6. Promoter activities of the metal-inducible promoters P*copA* and P*mntE* in the presence or absence of $CuSO_4$ and $MnSO_4$. Growth in OD_{620nm} (top row and solid lines) and luminescence signal in relative luminescence units normalized by optical density (RLU/OD_{620nm}) (bottom row and dotted lines) of the *S. pyogenes* reporter strains harboring the metal-inducible promoters P*copA* or P*mntE* or the control (no promoter) in the presence (data points with red or grey fill) and absence (data points with white fill) of different inducer compounds (80 µM $MnSO_4$ and 80 µM $CuSO_4$). Distilled water (dH_2O) was used as a negative control for induction. Experiments were performed in biological triplicates and measurements in technical duplicates.

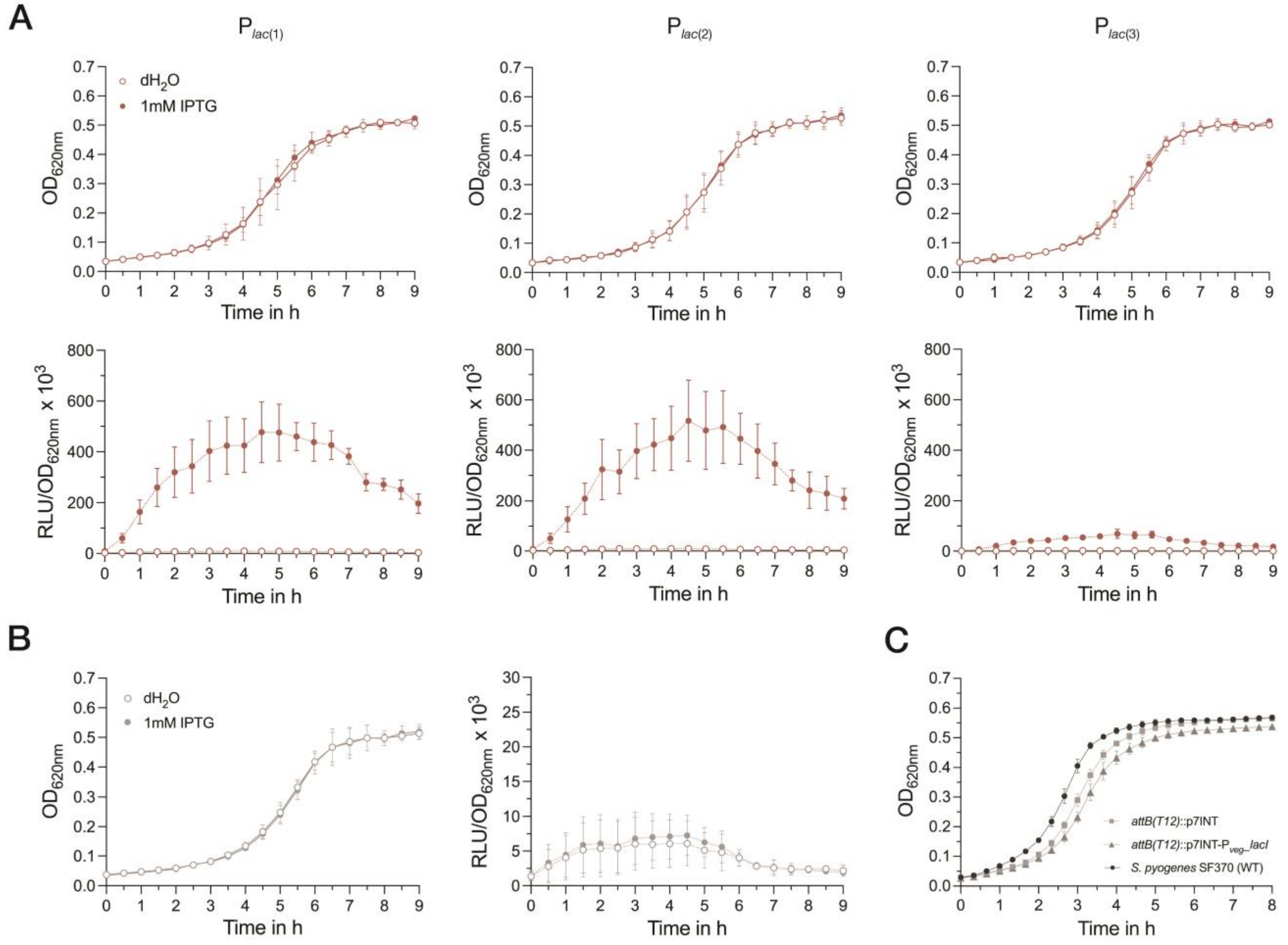

Supplementary Figure 7. Activity of IPTG-inducible P*lac(Spn)* promoters harboring different modifications within the translation initiation region in a LacI-expressing *S. pyogenes* strain. **(A)** *S. pyogenes* growth (solid lines) and luminescence signal (dotted lines) of the reporter strains harboring different metal-inducible promoters and the control (no promoter) in the presence (data points with red fill) and absence (data points with white fill) of 1 mM IPTG. Distilled water (dH_2O) was used as a negative control for induction. Growth is shown in OD_{620nm} over time (top row), while the luminescence signal is shown in relative luminescence units normalized by optical density (RLU/OD_{620nm}) (bottom row). **(B)** Growth shown in OD_{620nm} (left) and luminescence signal depicted in RLU/OD_{620nm} (right) for the control strain harboring the reporter plasmid without a promoter in the presence (data points with grey fill) and absence (data points with white fill) of 1 mM IPTG. **(C)** Growth curves of the LacI expression strain (EC3732) compared to *S. pyogenes* wildtype (EC2514) and *S. pyogenes* containing the empty p7INT plasmid (EC3241). Experiments were performed in biological triplicates and measurements in technical duplicates.

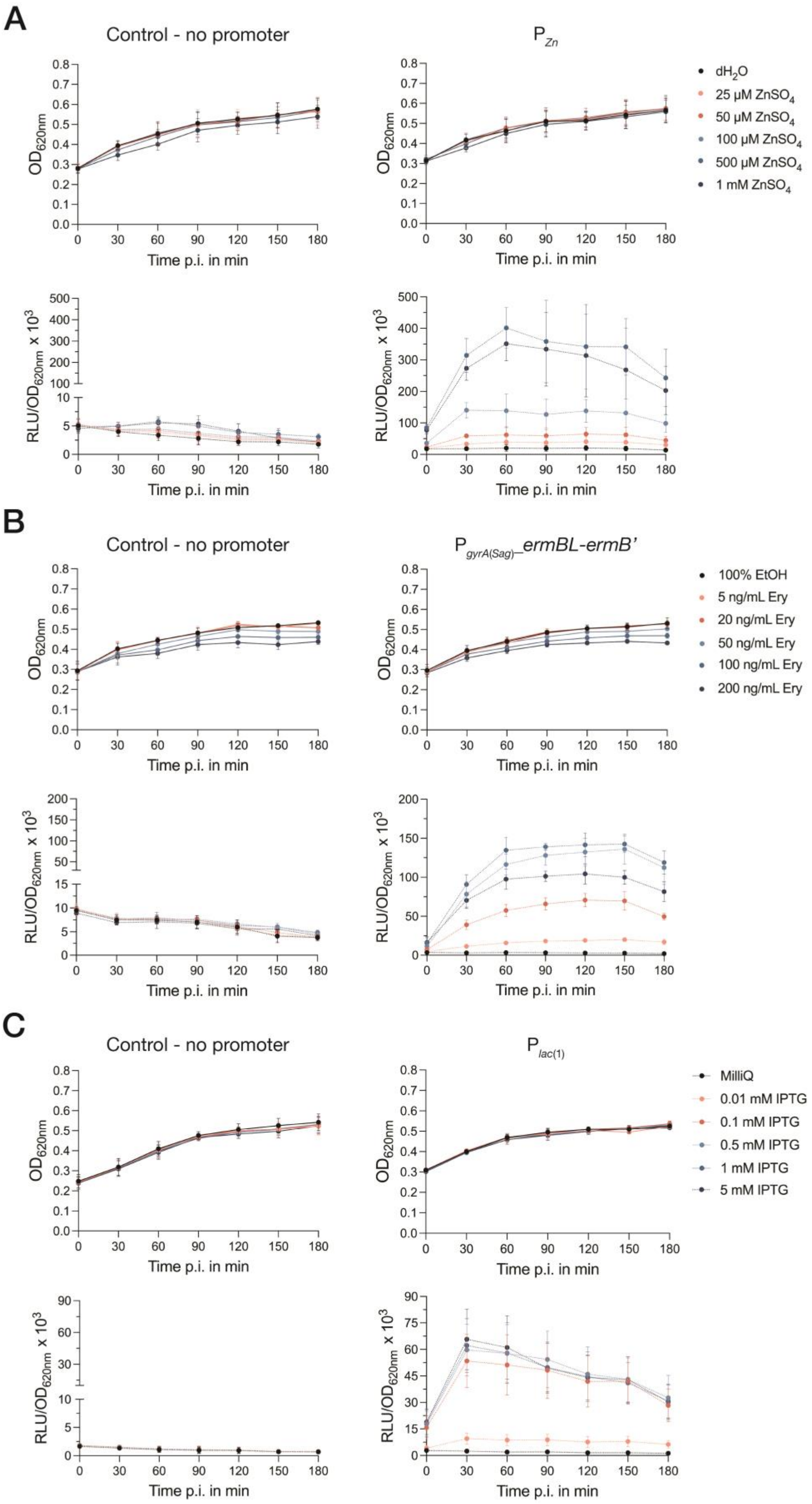
A
Control - no promoter
P Zn
dH2O
25 µM ZnSO4
50 µM ZnSO4
100 µM ZnSO4
500 µM ZnSO4
1 mM ZnSO4
OD620nm
RLU/OD620nm x 10^3
Time p.i. in min
B
Control - no promoter
P gyrA(Sag)–ermBL-ermB'
100% EtOH
5 ng/mL Ery
20 ng/mL Ery
50 ng/mL Ery
100 ng/mL Ery
200 ng/mL Ery
OD620nm
RLU/OD620nm x 10^3
Time p.i. in min
C
Control - no promoter
P lac(1)
MilliQ
0.01 mM IPTG
0.1 mM IPTG
0.5 mM IPTG
1 mM IPTG
5 mM IPTG
OD620nm
RLU/OD620nm x 10^3
Time p.i. in min

Supplementary Figure 8. Growth and luminescence signal of the three inducible systems P_{Zn}, $P_{gyrA(Sag)\_}$*ermBL-ermB'* and $P_{lac(1)}$ in response to increasing inducer concentrations. The legend to the right of each panel shows the applied inducer concentrations. Growth and luminescence signals are shown in OD_{620nm} or relative luminescence units normalized by optical density (RLU/OD_{620nm}) on the y-axis, while the time post induction (p.i.) is indicated on the x-axis. **(A)** *S. pyogenes* growth (top row and solid lines) and luminescence signal (bottom row and dotted lines) of the strain harboring either the control plasmid without promoter (left) or the zinc-inducible promoter (right). **(B)** *S. pyogenes* growth (top row and solid lines) and luminescence signal (bottom row and dotted lines) of the strain harboring either the control plasmid without promoter (left) or the erythromycin-inducible riboswitch (right). **(C)** *S. pyogenes* growth (top row and solid lines) and luminescence signal (bottom row and dotted lines) of the strain harboring either the control plasmid without promoter (left) or the reporter plasmid with the IPTG-inducible $P_{lac(1)}$ promoter (right). Experiments were performed in biological triplicates and measurements in technical duplicates.

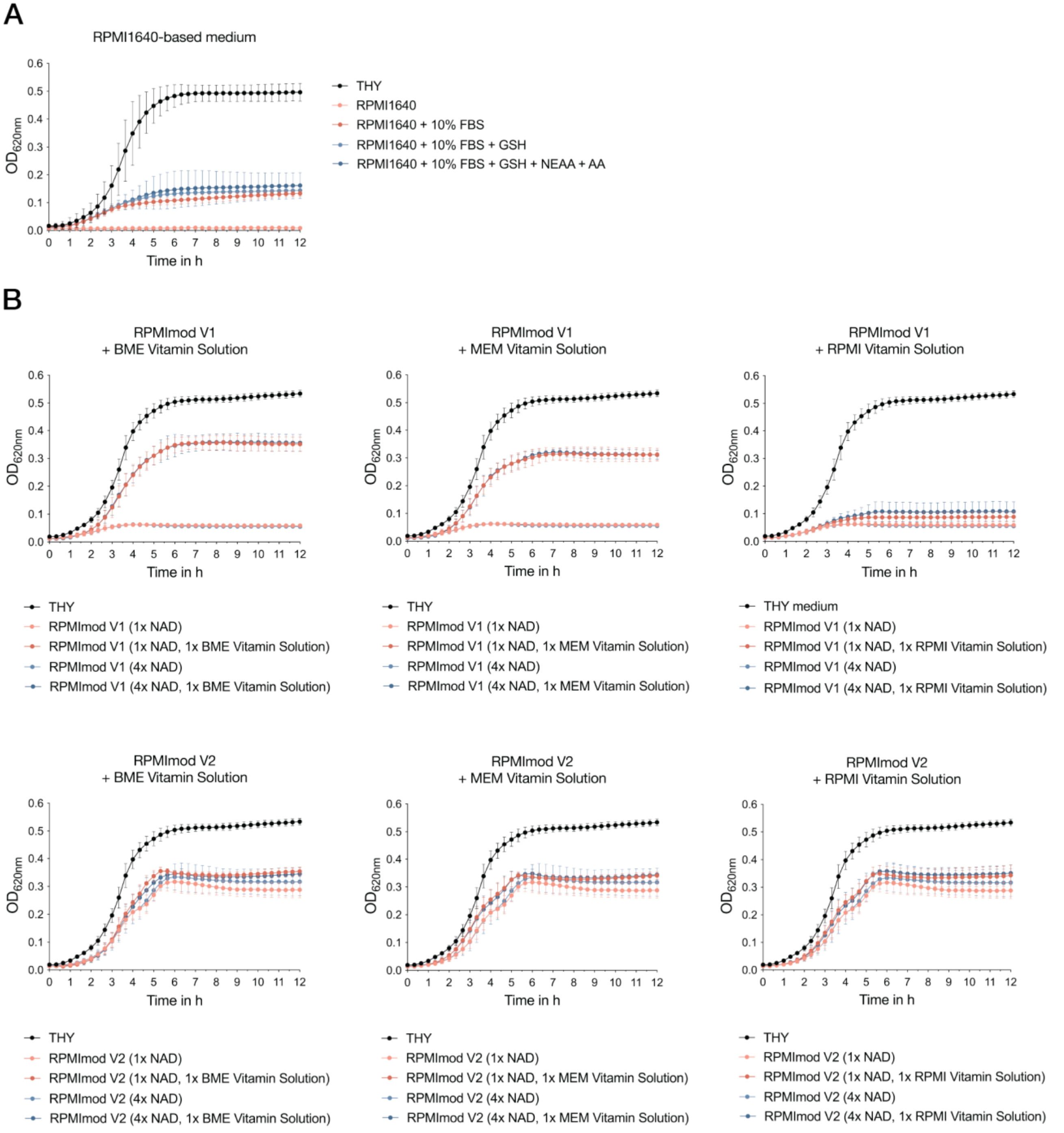

Supplementary Figure 9. Growth of *S. pyogenes* SF370 in different formulations of the RPMI4Spy chemically defined medium. The growth is shown in OD_{620nm} on the y-axis, while the time in hours is indicated on the x-axis. **(A)** Initial test experiment using RPMI1640 cell culture medium supplemented with glutathione, fetal bovine serum, RPMI non-essential amino acid solution or RPMI amino acid solution or combinations thereof. Abbreviations: GSH = Glutathione, NEAA = non-essential amino acids, AA = amino acids, FBS = fetal bovine serum **(B)** Test experiments with different RPMI4Spy medium formulations supplemented with either the commercial RPMI amino acid solution (V1) or a self-prepared amino acid mix (V2). Both basic formulations were further supplemented with different vitamin solutions (BME Vitamin Solution, MEM Vitamin Solution or RPMI Vitamin Solution) and

two different concentrations of niacinamide (NAD). Experiments were performed in biological triplicates. Abbreviations: RPMImod = modified RPMI1640.

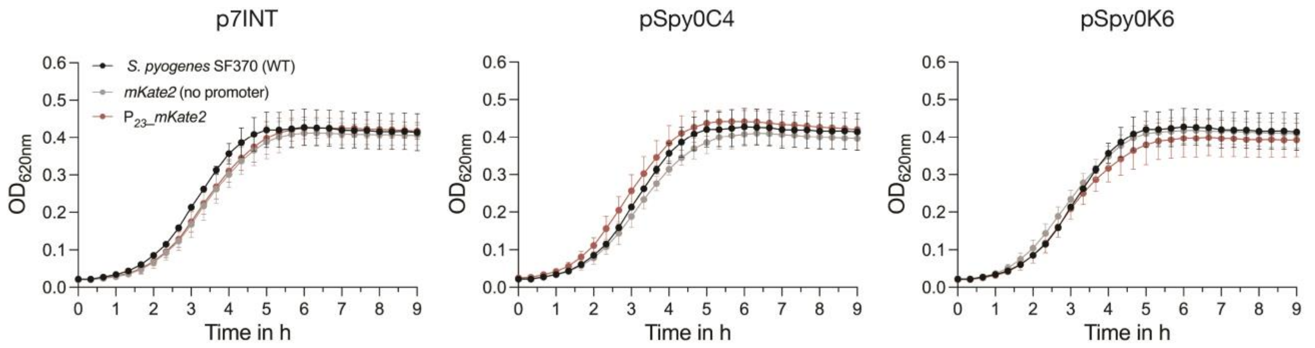

Supplementary Figure 10. Growth of strains harboring either the mKate2 reporter (P*23_mKate2*, EC3391) or the negative control without a promoter (no promoter, EC3480) integrated at different genomic locations compared to the *S. pyogenes* wildtype (EC2514). The reporter was integrated at the tmRNA locus (*attB*(T12)) using p7INT (left), into the *sagB* gene using pSpy0C4 (center) and into a transcriptionally silent open reading frame (*SPy_1078*) using pSpy0K6 (right). The growth is shown in OD_{620nm} on the y-axis, while the time in hours is indicated on the x-axis. Experiments were performed in biological triplicates and each measurement in technical duplicates.

Supplementary Table 1. Bacterial strains used and created in this study. 'Code' refers to the internal strain numbering system used in our laboratory. Symbols: (~) indicates translational fusions; (*) indicates an introduced stop codon (TAA)

Code	Strain Name	Features	Source	Alias Used
		**Wildtype strains**		
EC1	DH5*α*	Cloning host: *E. coli* K12 *fhuA2* Δ(*argF*-*lacZ*)U169 *phoA glnV44* Φ80 Δ(*lacZ*)M15 *gyrA96 recA1 relA1 endA1 thi-1 hsdR17*	New England Biolabs	
RDN204	OneShot™ TOP10	F-*mcrA* Δ(*mrr*-*hsd*RMS-*mcr*BC) Φ80*Lac*ZΔM15 Δ *Lac*X74 *rec*A1 *ara*D139 Δ(*araleu*) 7697 *gal*U *gal*K *rps*L (StrR) *end*A1 *nup*G	Invitrogen	
EC2621	BW25113 *lon*::*kanR*	*Δ(araD-araB)567 ΔlacZ4787*(::rrnB-3) *λ- rph-1 Δ(rhaD-rhaB)568 hsdR514 lon*::*kanR*	Hansen *et al.*, 2012 (1)	
EC2705	*Bacillus subtilis* PY79	wildtype, *trpC2* prototroph	Youngman *et al.*, 1984 (2)	
EC2514	*S. pyogenes* SF370	wildtype M1 strain	*Streptococcus pyogenes* Rosenbach ATCC700294	wildtype
		**Strains with empty integrative plasmids**		
EC3217-3218	EC2514 *amyA*::pSpy0C2	*amyA*::pSpy0C2	this study	n/a
EC3220-3221	EC2514 *sagB*::pSpy0C4	*sagB*::pSpy0C4	this study	n/a
EC3729-3730	EC2514 *Spy_1078*::pSpy0K6	SP*y_1078*::pSpy0K6	this study	n/a
EC3241-3242	EC2514 *attB*(T12)::p7INT	*attB*(T12)::p7INT (integration does not impair functionality)	this study	n/a
EC3741-3742	EC2514 *attB*(T12)::p7INT.1	*attB*(T12)::p7INT.1	this study	n/a
		**Reporter strains**		
EC3364-3365	EC2514 *attB*(T12)::p7INT-P_{23}*_mNeongreen~ssrA*	*attB*(T12)::p7INT-P_{23}*_mNeongreen~ssrA*, Erm^R	this study	native SsrA tag
EC3385-3386	EC2514 *attB*(T12)::p7INT-P_{23}*_mNeongreen*~ssrA*	*attB*(T12)::p7INT-P_{23}*_mNeongreen*~ssrA*, Erm^R	this study	mNeongreen reporter (untagged)

Code	Strain Name	Features	Source	Alias Used
EC3388-3389	EC2514 *attB*(T12)::p7INT-P_{23}_*mNeongreen~ssrA* (LDD)	*attB*(T12)::p7INT-P_{23}_*mNeongreen~ssrA* (LAA to LDD), Erm^R	this study	LDD variant
EC3486-3487	EC2514 *attB*(T12)::p7INT-P_{23}_*mNeongreen~ssrA* (ASV)	*attB*(T12)::p7INT-P_{23}_*mNeongreen~ssrA* (LAA to ASV), Erm^R	this study	ASV variant
EC3489-3490	EC2514 *attB*(T12)::p7INT-*mNeongreen*~STOP ~*ssrA*	*attB*(T12)::p7INT-*mNeongreen*~ssrA*, Erm^R	this study	mNeongreen control
EC3391-3392	EC2514 *attB*(T12)::p7INT-P_{23}_*mKate2*~ssrA*	*attB*(T12)::p7INT-P_{23}_*mKate2*~ssrA*, Erm^R	this study	mKate2 reporter
EC3480-3481	EC2514 *attB*(T12)::p7INT-*mKate*~ssrA*	*attB*(T12)::p7INT-*mKate*~ssrA*, Erm^R	this study	mKate2 control
EC3735-3736	EC2514 *SPy_1078*::pSpy0K6-P_{23}_*mKate2*~ssrA*	*SPy_1078*::pSpy0K6-P_{23}_*mKate2*~ssrA*, Kan^R	this study	n/a
EC3738-3739	EC2514 *SPy_1078*::pSpy0K6-*mKate2*~ssrA*	*SPy_1078*::pSpy0K6-*mKate2*~ssrA*, Kan^R	this study	n/a
EC3429-3430	EC2514 *sagB*::pSpy0C4-P_{23}_*mKate*~ssrA*	*sagB*::pSpy0C4-P_{23}_*mKate*~ssrA*, Cm^R	this study	n/a
EC3492-3493	EC2514 *sagB*::pSpy0C4-*mKate*~ssrA*	*sagB*::pSpy0C4-*mKate*~ssrA*, Cm^R	this study	n/a
		**Other**		
EC3732-3733	EC2514 *attB*(T12)::p7INT-P_{veg}_*lacI*	*attB*(T12)::p7INT-P_{veg}_*lacI*, Erm^R	this study	LacI expression strain
EC3756-3757	EC2514 *ΔsagA*	*ΔsagA*	this study	*sagA* mutant

Supplementary Table 2. Oligonucleotides used in this study. Overhangs used for cloning purposes are underlined. ‘Code’ refers to the internal oligo numbering system used in our laboratory.

Code	Name	5'-3'-Sequence
OLEC290	*mga*_F	TTAACCTCTGTTTGATTCGC
OLEC291	*mga*_R	GTCGTACTGACTTAACGAAA
OLEC4854	*ropB*_F	AGCGACTATCATCCGAAACAT
OLEC4855	*ropB*_R	GCCCTGGAGCTGTTGAGATA
OLEC4856	*csrRS*_F	TCGCTAGAAGACTATTTGACCAT
OLEC4857	*csrRS*_R	AAGACATCGCGATTGACAGT
OLEC9562	*ffluc*_fwd	GATCGGTCTCAGCTGAATTCAAAAAAAAATCTAGAAGGAGG AATAAAAAATGGAAGACG
OLEC9563	*ffluc*_rev	GATCGGTCTCTTCACTGCAGTACTAGTATTACAATTTGGACT TTCCGCCC
OLEC9589	P_{23}_goldenbrick_fwd	GATCGGTCTCAGCTGAATTCAAAAAAAAATCTAGACTCGAA AAGCCCTGACAACCC
OLEC9591	P_{gyrA}_goldenbrick_fwd	GATCGGTCTCAGCTGAATTCAAAAAAAAATCTAGATTTTTGT GTTGTTTAGTTGC
OLEC9592	P_{gyrA}_goldenbrick_rev	GATCGGTCTCTTCACTGCAGTACTAGTATTCTAGTCAATACT AGATTTC
OLEC9595	P_{tet}_goldenbrick_fwd	GATCGGTCTCAGCTGAATTCAAAAAAAAATCTAGAGATAAT GCCGACTGTACTTTTTAC
OLEC9596	P_{tet}_goldenbrick_rev	GATCGGTCTCTTCACTGCAGTACTAGTACTTAACTAGACTCG AAGATC
OLEC9749	P_{veg}_GBOH_fwd	GATCGGTCTCAGCTGAATTCAAAAAAAAATCTAGAGAGTTC TGAGAATTGGTATGC
OLEC9750	P_{veg}_GBOH_rev	GATCGGTCTCTTCACTGCAGTACTAGTAACTACATTTATTGT ACAACACGAGC
OLEC9751	P_{23}_GBOH_rev	GATCGGTCTCTTCACTGCAGTACTAGTACCAACATCATTGTC ATTCATATTTTTC
OLEC9773	pIB166_GG_M13_fwd	GATCCGTCTCTATGTGTCATAGCTGTTTCCTGCAAAGAATGG TGATGACAATTG
OLEC9774	pIB166_GG_M13_rev	GATCCGTCTCTGCTGACTGGCCGTCGTTTTACCATAAACACC AATAGCCTTAAC
OLEC9775	MCS_GG_fwd	GATCCGTCTCCCAGCGAGACCAGCTGAATTCAAAAA
OLEC9776	MCS_GG_rev	GATCCGTCTCGACATGAGACCTTCACTGCAGTACTAG
OLEC9777	pIB184_GG_M13_fwd	GATCCGTCTCTATGTGTCATAGCTGTTTCCTGGACTAGCAAA TACTAACAACAAGACAC
OLEC9778	pIB184_GG_M13_rev	GATCCGTCTCTGCTGACTGGCCGTCGTTTTACGACGCTCTTC CGCTTCCTC
OLEC9779	pIB185_GG_M13_fwd	GATCCGTCTCTATGTGTCATAGCTGTTTCCTGGATTCTAGAC GCTCTTCCG
OLEC9780	pIB185_GG_M13_rev	GATCCGTCTCTGCTGACTGGCCGTCGTTTTACCAACGTTAAT AAGACGTTGTC

Code	Name	5'-3'-Sequence
OLEC9781	*mrfp*_GBOH_fwd	GATCGAGACCAGCTGAATTCAAAAAAAAATCTAGATGGCCG GCGATTCATTAATGCAGCTGGC
OLEC9782	*mrfp*_GBOH_rev	GATCGAGACCTTCACTGCAGTACTAGTATTAACCGGTTATAA ACGCAGAAAGGCCCAC
OLEC9799	pIB184_*Esp*3Imut_fwd	CTTTTTGAACCCGTCGCCTTACGGCTTTATTAGATATGTAATC
OLEC9800	pIB184_*Esp*3Imut_rev	CCGTAAGGCGACGGGTTCAAAAAGGTTTAAATAAAGGAGA
OLEC9801	pIB166_*Esp*3Imut_fwd	AACGTCACAGAAACGATTTTCAGACGTTTTAATAAAAAATC
OLEC9802	pIB166_*Esp*3Imut_rev	GTCTGAAAATCGTTTCTGTGACGTTTTAGCGTTTATTTCGTTT AG
OLEC9824	kanR_GG_fwd	GATCCGTCTCTATGTGTCATAGCTGTTTCCTGCCTAATAATTT CTAGGTACTAAAAC
OLEC9825	kanR_GG_rev	GATCCGTCTCGTTAGCCTTATAGCTTGTAAATTCTATC
OLEC9826	pIB166_GG2_fwd	GATCCGTCTCTCTAATATGAGATAATGCCGACTGTAC
OLEC9827	ermR_GG_fwd	GATCCGTCTCTATGTGTCATAGCTGTTTCCTGCGAAATGATA CACCAATCAG
OLEC9828	ermR_GG_rev	GATCCGTCTCGTTAGCTTGCATATGATCCGAGCTTC
OLEC9829	pBAV1K-T5_fwd	GATCCGTCTCTATGTGTCATAGCTGTTTCCTGCTAGACCTAG TGTCATTTTATTTC
OLEC9830	pBAV1K-T5_rev	GATCCGTCTCTGCTGACTGGCCGTCGTTTTACGTTATACGCC AACTTTGAAAAC
OLEC9831	pBAV1K-T5_mut_fwd	ACGTCTGAGAAACGATTTTCAGACGTTTTAATAAAAAATC
OLEC9832	pBAV1K-T5_mut_rev	ACGTCTGAAAATCGTTTCTCAGACGTTTTAGCGTTTATTTC
OLEC9833	pBAV1K-T5_GG2_ fwd	GATCCGTCTCCCAGCGTTTTCAAAGTTGGCGTATAAC
OLEC9834	pBAV1K-T5_GG2_rev	GATCCGTCTCGACATTACTGGATGAATTGTTTTAGTAC
OLEC9835	cmR_GG2_fwd	GATCCGTCTCTATGTCAAAGAATGGTGATGACAATTG
OLEC9836	cmR_GG2_rev	GATCCGTCTCTGCTGTTATAAAAGCCAGTCATTAGGC
OLEC9837	ermR_GG2_fwd	GATCCGTCTCTATGTCGAAATGATACACCAATCAG
OLEC9838	ermR_GG2_fwd	GATCCGTCTCTGCTGCTTGCATATGATCCGAGCTTC
OLEC9875	pIB185_*Esp*3Imut_fwd	CTGGATTACATATCTAATAAAGCCGTAAGAAGACGGGTTC
OLEC9876	pIB185_*Esp*3Imut_rev	CTTTATTTAAACCTTTTTGAACCCGTCTTCTTACGGCTTTA
OLEC9877	pIB185new_fwd	GATCCGTCTCCCAGCGAAGCTCGGATCATATGCAAG
OLEC9878	pIB185new_rev	GATCCGTCTCGACATCTGATTGGTGTATCATTTCG
OLEC10024	*lux*_new*Bsa*IOH_fwd	GATCGGTCTCAGTGAAATTCAAAAAAAAATC
OLEC10026	P_{23}_new*Bsa*IOH_fwd	GATCGGTCTCATCAGAATTCAAAAAAAAATC
OLEC10027	P_{23}_new*Bsa*IOH_rev	GATCGGTCTCTTCACTGCAGTACTAG
OLEC10182	*ffluc*_new*Bsa*IOH_rev	GATCGGTCTCTCACACTGCAGTACTAGTA
OLEC10311	MCS+CmR_fwd	GATCGATCCGTCTCCCAGCGAACGAATATTGGATAAATATG
OLEC10312	MCS+CmR_rev	GATCGATCCGTCTCGACATATCTGGAGCTGTAATATAAAAAC
OLEC10315	pBR322ori_fwd	GATCGATCCGTCTCTCTAACTGTCAGACCAAGTTTACTCATA T

**Code**	**Name**	**5'-3'-Sequence**
OLEC10316	pBR322ori_rev	GATCGATCCGTCTCTAGGTCTTTCCAGTCGGGAAACCTGTC
OLEC10930	*amyA*_up_fwd	GATCGATCCGTCTCGACCTGAATCAAGATTATTCTAGACGC
OLEC10931	*amyA*_up_rev	GATCGATCCGTCTCTGCTGTTAGTTACTAAACACAATATATCC
OLEC10932	*amyA*_down_fwd	GATCGATCCGTCTCTATGTATGGTAGAGAACCAAAGACAACAG
OLEC10933	*amyA*_down_rev	GATCGATCCGTCTCGTTAGCTGTCGCACCATCAACGGTTG
OLEC11104	pSpy1C+tt0_OH_fwd	CCGGCGGCAACCGAGCGTTCTGAATTATGGGGATGATGTTAAGGC
OLEC11105	pSpy1C+tt0_OH_rev	CGCCGGGCGTTTTTTATGTTATCCAATATTCGTTCCTTA
OLEC11463	pSpy0C3MCS_CmR_f	CAACTTTTTTGGATTTGATTATTTGGAACGAATATTGGATAAATATGGG
OLEC11464	pSpy0C3MCS_CmR_r	CCTTAATTCTAACATAGTTTTTATTCCATCTGGAGCTGTAATATAAAAACCT
OLEC11465	pSpy0C3*mazEF*_dw_f	TTTTTATATTACAGCTCCAGATGGAATAAAAACTATGTTAGAATTAAGG
OLEC11466	pSpy0C3*mazEF*_dw_r	GAGTAAACTTGGTCTGACAGACTGGAATTTGTTTTAGGGGT
OLEC11467	pSpy0C3pBR322ori_f	CCCTAAAACAAATTCCAGTCTGTCAGACCAAGTTTACTC
OLEC11468	pSpy0C3pBR322ori_r	AATGTTGAAGCTATCGTTCCTTTCCAGTCGGGAAAC
OLEC11469	pSpy0C3*mazEF*_up_f	GTTTCCCGACTGGAAAGGAACGATAGCTTCAACATTTAATCAG
OLEC11470	pSpy0C3*mazEF*_up_r	CCCATATTTATCCAATATTCGTTCCAAATAATCAAATCCAAAAAAGTTGAC
OLEC11471	pSpy0C4MCS_CmR_f	AGGTACTAGATAGTACCTGCGAACGATAAAAAACGCCCG
OLEC11472	pSpy0C4MCS_CmR_r	GATATTTCATTGAGACTCCTTAGATCTGGAGCTGTAATATAAAAACC
OLEC11473	pSpy0C4F_dw_f	GTTTTTATATTACAGCTCCAGATCTAAGGAGTCTCAATGAAATATC
OLEC11474	pSpy0C4_dw_r	GCGTTTTTTATGTCTGACAGGGTTACTCGTCAAGGAG
OLEC11475	pSpy0C4pBR322ori_f	AACTCCTTGACGAGTAACCCTGTCAGACATAAAAAACGCC
OLEC11476	pSpy0C4pBR322ori_r	CAAGGAGGAAGTCCACTGCTTTCCAGTCGGGAAACCTG
OLEC11477	pSpy0C4_up_f	GGTTTCCCGACTGGAAAGCAGTGGACTTCCTCCTTGG
OLEC11478	pSpy0C4_up_r	CGGGCGTTTTTTATCGTTCGCAGGTACTATCTAGTACCTGC
OLEC11479	pSpy0C4_dw_r	GAGTAAACTTGGTCTGACAGGGTTACTCGTCAAGGAG
OLEC11480	pSpy0C4pBR322ori_f	AACTCCTTGACGAGTAACCCTGTCAGACCAAGTTTACTC
OLEC11535	pSpy0C4_*sagB*_up_r	CGGGCGTTTTTTATCGTTCCGCGAGTCGTTTATTTTTAACC
OLEC12102	*mKate2*_GibsOH_F	TCGCATGCTCCTCTAGACTCGAGCCAGTGAATTCATGGTTTCAGAACT
OLEC12103	*mKate2*_GibsOH_R	CGCGTTGGGAGCTCTCCGGATCCCATGATTACGCCAAGCTTGCA
OLEC12104	*mNeon*_GibsOH_F	TCGCATGCTCCTCTAGACTCGAGCAGTGAATTCATGGTTTCAAAAGGTG

Code	Name	5'-3'-Sequence
OLEC12105	p7INT_*mKate2*_OH_F	TGCAAGCTTGGCGTAATCATGGGATCCGGAGAGCTCCCAACGC
OLEC12106	p7INT_*mKate2*_OH_R	AGTTCTGAAACCATGAATTCACTGGCTCGAGTCTAGAGGAGCATGCGA
OLEC12107	p7INT_*mNeon*_OH_R	CACCTTTTGAAACCATGAATTCACTGCTCGAGTCTAGAGGAGCATGCGA
OLEC12108	*mKate2*_F	CTTACCAGTGAATTCATGGTTTCAG
OLEC12109	*mNeongreen*_F	CTTACAGTGAATTCATGGTTTCAAAAG
OLEC12110	*mKate2_mNeon*_R	CTTACATGATTACGCCAAGCTTGC
OLEC12196	pSpy0C4_*mKate2*_F	GTTTCAGAACTTATCAAAGAAAAC
OLEC12197	pSpy0C4_R	GAATTCAGCTGGTCTCGCT
OLEC12198	pSpy0C4_*mNg*_F	GTTTCAAAAGGTGAAGAAGAC
OLEC12228	p7INT_*mNg*_F	GTTTCAAAAGGTGAAGAAGACAA
OLEC12229	p7INT_*mNg_mK2*_R	CCTATAGTGAGTCGTATTACAAT
OLEC12230	p7INT_*mK2*_F	GTTTCAGAACTTATCAAAGAAAA
OLEC12261	P$_{23}$-5'UTR_0C4OH_F	ATATGGGGATGATGTTAAGGCTACGAATTGGAGCTCTCGAAAAGC
OLEC12262	P$_{23}$-5'UTR_*mNg*_OH_R	TCTTCTTCACCTTTTGAAACCATTTTTTATTCCTCCTTAGATCGCCG
OLEC12263	P$_{23}$-5'UTR_*mK2*_OH_R	TCTTTGATAAGTTCTGAAACCATTTTTTATTCCTCCTTAGATCGCCG
OLEC12264	P$_{23}$-5'UTR_p7INT_OH_F	ACGACTCACTATAGGGCGAATTGCGAATTGGAGCTCTCGAAAAGC
OLEC12265	*mNg*_P$_{23}$Gibs_F	ATGGTTTCAAAAGGTGAAGAAG
OLEC12266	*mKate2*_P$_{23}$Gibs_F	ATGGTTTCAGAACTTATCAAAG
OLEC12267	p7INT_Gibs_R	CAATTCGCCCTATAGTGAGTC
OLEC12268	pSpy0C4_Gibs_R	TAGCCTTAACATCATCCCCA
OLEC13040	Fmuta *ssrA* LAA to ASV	CAAACTCTTACGCTGCCTCTGTTTAAGATCGATCTGG
OLEC13041	Rmuta *ssrA* LAA to ASV	CCAGATCGATCTTAAACAGAGGCAGCGTAAGAGTTTG
OLEC13042	Fmuta *ssrA* LAA to LDD	CAAACTCTTACGCTTTAGATGATTAAGATCGATCTGG
OLEC13043	Rmuta *ssrA* LAA to LDD	CCAGATCGATCTTAATCATCTAAAGCGTAAGAGTTTG
OLEC13044	Fmuta STOP *mNeon*	CGAACTTTACAAATAAAATACAAACTC
OLEC13045	Rmuta STOP *mNeon*	GAGTTTGTATTTTATTTGTAAAGTTCG
OLEC13046	Fmuta STOP *mKate2*	GGTCACCGTGCATAAAATACAAACTC
OLEC13047	Rmuta STOP *mKate2*	GAGTTTGTATTTTATGCACGGTGACC
OLEC13445	P$_{nisA}$_F	AGTCTTATAACTATACTGACAAT
OLEC13446	P$_{nisA}$_R	TTTGAGTGCCTCCTTATAATT
OLEC13447	*nisRK*_F	ATTGTCAGTATAGTTATAAGACTTATAGTGTGTATCTCAATCC
OLEC13448	*nisRK*_R	GAAGCTAGAGTAAGTAGTTC

Code	Name	5'-3'-Sequence
OLEC13449	1C_*ffluc*_P_{nisA}OH_F	AATTATAAGGAGGCACTCAAAATGGAAGACGCCAAAAACAT
OLEC13450	1C_*ffluc*_*nisRK*OH_R	GAACTACTTACTCTAGCTTCGACTGGCCGTCGTTTTACC
OLEC13456	P_{Tre}_F	AGGGTAAATTACCTTTCCAT
OLEC13457	P_{Tre}_R	GTGACATCCCCTAGTCCTTT
OLEC13458	1C_*ffluc*_P_{Tre}OH_F	AGGACTAGGGGATGTCACATGGAAGACGCCAAAAACAT
OLEC13459	1C_*ffluc*_P_{Tre}OH_R	ATGGAAAGGTAATTTACCCTGACTGGCCGTCGTTTTACC
OLEC13460	Pzn_F	ATCGGACACTTTCTAAATG
OLEC13461	Pzn_R	AATTTTCTCCTTTTGTTATAC
OLEC13462	1C_*ffluc*_P_{Zn}OH_F	AGTATAACAAAAGGAGAAAATTATGGAAGACGCCAAAAACAT
OLEC13463	1C_*ffluc*_P_{Zn}OH_R	AACATTTAGAAAGTGTCCGATGACTGGCCGTCGTTTTACC
OLEC13464	*lacI*_F	TTATTGTCCACTTTCCAAACG
OLEC13465	*lacI*_P_{lacI}OH_R	CTCACAATTACTAGTGAATTCGACTGTACGTCATCAGAAGTT
OLEC13466	P_{lacI}_F	GAATTCACTAGTAATTGTGAG
OLEC13467	P_{lacI}_R	AGATCCTTTCTCCTCTTTAGA
OLEC13468	1C_*ffluc*_P_{lacI}OH_F	TAAAGAGGAGAAAGGATCTATGGAAGACGCCAAAAACAT
OLEC13469	1C_*ffluc*_P_{lacI}OH_R	TTTGGAAAGTGGACAATAAGACTGGCCGTCGTTTTACC
OLEC13494	*ffluc*_0C4GibsOH_F	GTAAAACGACGGCCAGTCGAATTCAAAAAAAAATCTAGAAG
OLEC13495	*ffluc*_0C4GibsOH_R	CAGGAAACAGCTATGACCACTGCAGTACTAGTATTAC
OLEC13496	pSpy0C4_Gibs_F	GTCATAGCTGTTTCCTGC
OLEC13497	pSpy0C4_Gibs_R	GACTGGCCGTCGTTTTAC
OLEC14306	MCS_F	CGAATTATTAACGCTTACGGCTCACCTTCGGGTGGGCCTTTCTGCGTTTATACCTAGGGATATAGTAAAACGACGGCCAG
OLEC14307	MCS_R	GGAAGAGCGCCCAATACCAGGAAACAGCTATGAC
OLEC14308	p7INT_F	GTATTGGGCGCTCTTCC
OLEC14309	p7INT_R	GTAAGCGTTAATAATTCGAGC
OLEC14310	Mut_*int*_F	GAACTTGTTGAAAAAACTGACAACATTGAAAAAATAG
OLEC14311	Mut_*int*_R	CAACAAGTTCAAGGAATTGTTTAAGTTGATCAAGTG
OLEC14312	P_{veg}_F	GCGGGTTTTTTATTTTAC
OLEC14313	P_{veg}_R	GTTTCATTTTTTATTCCTCCTTTAACTACATTTATTGTACAAC
OLEC14314	*lacI*_F	AAGGAGGAATAAAAAATGAAACCAGTCACCCTTTAC
OLEC14315	*lacI*_R	CTATAGAATACTCAAGCTATGAAAAAAGGCCCACTTTTGTGGGCCTTTTTTGATCGATCGATCTTATTGTCCACTTTCCAAACG
OLEC14316	p7INT(*lacI*)_F	CATAGCTTGAGTATTCTATAGTG
OLEC14317	p7INT(P_{veg})_R	ATGTAAAATAAAAAACCCGCCCCTATAGTGAGTCGTATTAC
OLEC14322	0C5_Frag3_F	CTGTCAGACCAAGTTTACTC
OLEC14323	0C5_Frag3_R	CTTTCCAGTCGGGAAACC
OLEC14326	0C5_LFH_R	CTATATCCCTAGGTATAAACG

Code	Name	5'-3'-Sequence
OLEC14327	0K6_MCS_F	GTTTGGATATAGTAAAACGACGGCCAGTC
OLEC14328	0K6_MCS_R	CAAGTTTTGGGATTGTTAAGCAGGAAACAGCTATGAC
OLEC14329	0K6_kanR_F	CTTAACAATCCCAAAACTTG
OLEC14330	0K6_kanR_R	AAGTCAATGGTTAGATTCACCCTATCTAGCGAACTTTTAG
OLEC14331	0K6_HRup_F	TGAAAAAGACTGAGGGTC
OLEC14332	0K6_HRup_R	GTCGTTTTACTATATCCAAACGCAGAAAGGCCCACCCGAAGGTGAGCCCATAATTCCATATCAATTTTTTA
OLEC14333	0K6_ori_F	CTGTCAGACCAAGTTTACTC
OLEC14334	0K6_ori_R	GACCCTCAGTCTTTTTCACTTTCCAGTCGGGAAACC
OLEC14335	0K6_HRdown_F	GTGAATCTAACCATTGACTT
OLEC14336	0K6_HRdown_R	GTAAACTTGGTCTGACAGGTCAAGTTCATCTTGGCAT
OLEC14337	0K6_LFH_R	GTCGTTTTACTATATCCAAAC
OLEC14357	1C-tt0_*ffluc*_F	ATGGAAGACGCCAAAAAC
OLEC14358	1C-tt0_*ffluc*_R	GACTGGCCGTCGTTTTAC
OLEC14359	P_{Mn}_F	GTAAAACGACGGCCAGTCCATCTCACCTACTTCCGTA
OLEC14360	P_{Mn}_R	GTTTTTGGCGTCTTCCATAATAACTCCTTTGATTTCAAC
OLEC14363	P_{Cu}_F	GTAAAACGACGGCCAGTCCATAAGGTCAGTAATAGACC
OLEC14364	P_{Cu}_R	GTTTTTGGCGTCTTCCATAACGTCACCTTCATTCAC
OLEC14365	pSpy2C_bb_F	CATATTCACCAATAGCCTTAAGTAAAACGACGGCCAGTC
OLEC14366	pSpy2C_bb_R	TTATAAAAGCCAGTCATTAGGC
OLEC14367	pSpy2C_pAMb1_F	CTAATGACTGGCTTTTATAAATGTCTGGTCCCTAGCGCTTAGAA
OLEC14368	pSpy2C_pAMb1_R	TTAAGGCTATTGGTGAATATGGTACCGAGCTCGAATTCAG
OLEC14369	pSpy2E_bb_R	AAGCGACTCATAGAATTAT
OLEC14370	pSpy2E_pAMb1_F	ATAATTCTATGAGTCGCTTTGGTCCCTAGCGCTTAGAA
OLEC14377	P_{ermB}_F	GCCTATCATTTTCAATGAAAG
OLEC14380	1C-tt0-*ffluc*2_R	CTTTCATTGAAAATGATAGGCGACTGGCCGTCGTTTTAC
OLEC14398	Ori_1_F	CTGTCAGACCAAGTTTACTCATATATAC
OLEC14399	Ori_1_R	GTTAAAATACAATTACTATAACCAAAAATGAGCACCTGTTCAATAGTGAACAACATTTAGAAAGTGTCCGATGAGAGGCGGTTTGCGTATTG
OLEC14402	Ery_3_F	ATCCCCCACAAAAAGAAAAAC
OLEC14403	Ery_3_R	CTGGCCGTCGTTTTACGTTTTAGATTTACCTAATAATTTATCTAC
OLEC14404	MRFP1_4_F	GTAAAACGACGGCCAGTCAGC
OLEC14405	MRFP1_4_R	GAGTAAACTTGGTCTGACAGCAGGAAACAGCTATGACACATGAG
OLEC14522	ErmB_3_F_new	AAACGAAATGATACACCAATCAG
OLEC14523	Ori_1_R_new	aaagagaggcggtttgcgtattg

Code	Name	5'-3'-Sequence
OLEC14524	P_{veg}_frag_2_F	caatacgcaaaccgcctctc
OLEC14525	Pterm_frag_2_R	ATTGGTGTATCATTTCG
OLEC14502	OC5_F1_R_new	CCCATTTAAGAGCGCATAGATCTGGAGCTGTAATATAAAAA C
OLEC14503	OC5_F2_F_new	CTATGCGCTCTTAAATGGG
OLEC14504	OC5_F2_R_new	GTAAACTTGGTCTGACAGGTAGCATGACTGTATGTATCAAG
OLEC14505	OC5_F4_F_new	GGTTTCCCGACTGGAAAGATGAGTTCGGGCTGTTTAGTAG
OLEC14506	OC5_F4_R_new	CTATATCCCTAGGTATAAACGCAGAAAGGCCCACCCGAAGG TGAGCCGTGCGTAGTGCTTGTAAGG
OLEC14548	P_{lac}_mut_F	GTAAAACGACGGCCAGTCTTATAGAATTCACTAGTAATTG
OLEC14549	P_{lac}_mut1_R	GTTTTTGGCGTCTTCCATATGTTCCTCCTTAGATCTTTTGAAT TCG
OLEC14550	P_{lac}_mut2_R	GTTTTTGGCGTCTTCCATATGTTTCCTCCTTAGATCTTTTGAA TTCG
OLEC14551	P_{lac}_mut3_R	GTTTTTGGCGTCTTCCATATGTTCCTCTTTAGATCTTTTGAAT TCG
OLEC14582	*ermB*_UTR_new_R	GTTTTGAGAATATTTTATATTTTTG
OLEC14583	1C-*ffluc*_*ermB*OH_F	CAAAAATATAAAATATTCTCAAAACGAAGACGCCAAAAACA TAAAG
OLEC14584	P_{xylS2}_F	GAATTAGATATTTAAAAGTATCAT
OLEC14585	P_{xylS2}_R	TGATTTAAGTGAACAAGTTTATC
OLEC14586	1C-tt0-*ffluc*_P_{xylS2}_F	AAACTTGTTCACTTAAATCAAGAATTAAATTAAAAAGGGAG GCC
OLEC14587	1C-tt0-*ffluc*_P_{xylS2}_R	ATGATACTTTTAAATATCTAATTCCTGACTGGCCGTCGTTTTA
OLEC14589	P_{veg}_mut1_F	CTTATTAACATGGATATAATTTAAATTTTATTTGAC
OLEC14590	P_{veg}_mut1_R	GTTAATAAGAAAATGTCAATAAAATTATTTTGAC
OLEC14591	P_{veg}_mut2_F	GACAAAACGTCTTATTAACGTTGATATAATTTAAA
OLEC14592	P_{veg}_mut2_R	GACGTTTTGTCAATAAAATTATTTTGACAAAATTC
OLEC14593	P_{veg}_mut3_F	CAACGTCATTAACGTTGATATAATTTAAATTTTATTTG
OLEC14594	P_{veg}_mut3_R	GTTAATGACGTTGTCAATAAAATTATTTTGAC
OLEC14717	P_{23}_*mKate2*_F	CGAGACCAGCTGAATTCCTCGAAAAGCCCTGACAACCC
OLEC14718	*mKate2*_R	GACACATGAGACCTTCAGATCCGAGAAAAAAGGCCCAC
OLEC14719	0K6_Gibs_F	TGAAGGTCTCATGTGTCATAG
OLEC14720	0K6_Gibs_R	GAATTCAGCTGGTCTCGCTG
OLEC14721	*mKate2*_F	CGAGACCAGCTGAATTCATGGTTTCAGAACTTATCAAAGAA AAC
OLEC14726	RBS_F	AAGGAGGAATAAAAAATGGACCTTCAAGCTCAATTG
OLEC14727	RBS_R	TTTTTATTCCTCCTTTAACTACATTTATTGTACAAC
OLEC14875	*sagA*_up_F	GATCCTTCCTCCTTGGTTTTAAG
OLEC14876	*sagA*_up_R	GATCCAACTATCTAGTTCTTATCAC

Code	Name	5'-3'-Sequence
OLEC14877	*sagA*_do_F	GATCCTATTTAGCATCTCTATGTG
OLEC14878	*sagA*_do_R	GATCCAATTTTCAGCATTAATAGC
OLEC14879	*sagA*_up_OH_F	CTGAATTCAAAAAAAATCTAGATGCTTCCTCCTTGGTTTTAAG
OLEC14880	*sagA*_up_OH_R	CAACTATCTAGTTCTTATCAC
OLEC14881	*sagA*_do_OH_F	GTGATAAGAACTAGATAGTTGCTATTTAGCATCTCTATGTG
OLEC14882	*sagA*_do_OH_R	CTATGACACATGAGACCTTCCAATTTTCAGCATTAATAGC
OLEC14883	pERASE_F	GAAGGTCTCATGTGTCATAG
OLEC14884	pERASE_R	CATCTAGATTTTTTTTGAATTCAG

Supplementary Table 3. Plasmids used in this study. Information on all plasmids used either for cloning purposes or for *in vivo* experiments including the plasmid names ('designation'), their encoded resistance genes ('marker') and a short description of their most important characteristics ('features'). 'Code' refers to the internal plasmid numbering system used in our laboratory. Symbols: (~) indicates translational fusions; TA = Toxin-Antitoxin; (*) indicates stop codon (TAA), except for *pheS**, where (*) highlights the introduction of two mutations.

Code	Designation	Marker	Reference	Features
		**Plasmids used for cloning purposes**		
pEC536	pEU8517	Kan^R, Cm^R	Bugrysheva & Scott 2010 (3)	$P_{tet}(O)_3$ cassette, *cat86*, Ω-Km2 element, pUC ori
pRDN18	pMSP3535	Erm^R	Bryan *et al.* 2000 (4)	*ermB*, *nisRK* operon, P*nisA* promoter, pAMβ1 replicon, pUC ori
pEC2173	pLZ12Km2-P*23*R:TA:*ffluc*	Kan^R	Loh & Proft 2013 (5)	*aph(3')-IIIa*, ω-ε-ζ (TA cassette of pBT286), pSH71 replicon, *ffluc* under control of P_{23}
pEC2192	pBAV1K-T5-*gfp*	Kan^R	Bryksin *et al.* 2010 (6)	*aph(3')-IIIa*, P_{T5}_*gfp*, modified pWV01 replicon
pEC2466	pIB166	Cm^R	Biswas *et al.* 2008 (7)	*cat*, pSH71 replicon, P_{23} promoter
pEC2467	pIB167	Cm^R	Biswas *et al.* 2008 (7)	*cat*, pSH71 replicon, P_{spac} promoter, coding regions for N-terminal His-tag and C-terminal Strep-tag
pEC2468	pIB184-Km	Kan^R	Biswas *et al.* 2008 (7)	*aph(3')-IIIa*, pAMβ1 replicon, P_{23} promoter, pUC ori
pEC2469	pIB185	Erm^R	Biswas *et al.* 2008 (7)	*ermB*, pAMβ1 replicon, P_{veg} promoter, pUC ori
pEC2471	pBS3C*lux*	Cm^R	Radeck *et al.* 2013 (8)	*bla*, *cat*, *luxABCDE* operon, pUC ori, MCS with $P_{lac(Eco)}$_*mrfp*, integrates into *sacA* in *B. subtilis*
pEC3003	pJWV102-P_{lac}_*dcas9sp*	Amp^R, Tet^R	Liu *et al.* 2017 (9)	*dcas9* under control of IPTG inducible promoter (P_{lac}), *bla*, integrates into *bgaA* in *S. pneumoniae*, *tetM*
pEC3004	pPEPY-PF6_*lacI*	Kan^R, Gm^R	Liu *et al.* 2017 (9)	*lacI-aacC1* under control of P_{F6} promoter, *aph(3')-IIIa*, integrates into *prsA* in *S. pneumoniae*
pEC3045	pLZ12Km2-TA:$P_{gyrA(Sag)}$-*ermBL*-*ermB'*:A2I:*ffluc*-tt3	Kan^R	Wulff *et al.* 2023 (10)	*aph(3')-IIIa*, ω-ε-ζ (TA cassette of pBT286), *ffluc* under control of erythromycin-inducible riboswitch $P_{gyrA(Sag)}$-*ermBL-ermB'*, pSH71 replicon
		**Replicative backbones**		
pEC2483	pSpy1C	Cm^R	This study	pSH71 replicon, *cat*, $P_{lac(Eco)}$_*mrfp* in MCS
pEC2485	pSpy1K	Kan^R	This study	pSH71 replicon, *aph(3')-IIIa*, $P_{lac(Eco)}$_*mrfp* in MCS
pEC2484	pSpy1E	Erm^R	This study	pSH71 replicon, *ermB*, $P_{lac(Eco)}$_*mrfp* in MCS

Code	Designation	Marker	Reference	Features
pEC3112	pSpy2C	Cm^R	This study	pAMβ1 replicon, *cat*, P$_{lac(Eco)}$_*mrfp* in MCS, pUC ori
pEC3113	pSpy2E	Erm^R	This study	pAMβ1 replicon, *ermB*, P$_{lac(Eco)}$_*mrfp* in MCS, pUC ori
pEC2498	pSpy3C	Cm^R	This study	modified pWV01 replicon, *cat*, P$_{lac(Eco)}$_*mrfp* in MCS
pEC2488	pSpy3K	Kan^R	This study	modified pWV01 replicon, *aph(3')-IIIa*, P$_{lac(Eco)}$_*mrfp* in MCS
pEC2506	pSpy3E	Erm^R	This study	modified pWV01 replicon, *ermB*, P$_{lac(Eco)}$_*mrfp* in MCS
**Integrative backbones**				
pEC2735	pSpy0C2	Cm^R	This study	pUC ori, *cat*, flanking regions for allelic replacement of *amyA* locus, MCS with P$_{lac(Eco)}$_*mrfp*
pEC2794	pSpy0C3	Cm^R	This study	pUC ori, *cat*, flanking regions for allelic replacement of *SPy_0938-0939*, MCS with P$_{lac(Eco)}$_*mrfp*
pEC2796	pSpy0C4	Cm^R	This study	pUC ori, *cat*, flanking regions for allelic replacement of *sagB*, MCS with P$_{lac(Eco)}$_*mrfp*
pEC3077	pSpy0C5 V1	Cm^R	This study	pUC ori, *cat*, flanking regions for allelic replacement of *SPy_1930*, MCS with P$_{lac(Eco)}$_*mrfp*
pEC3138	pSpy0C5 V1	Cm^R	This study	pUC ori, *cat*, adapted flanking regions for allelic replacement of *SPy_1930*, MCS with P$_{lac(Eco)}$_*mrfp*
pEC3078	pSpy0K6	Kan^R	This study	pUC ori, *aph(3')-IIIa*, flanking regions for allelic replacement of *SPy_1078*, MCS with P$_{lac(Eco)}$_*mrfp*
pEC2825	p7INT	Erm^R	McShan et al. 1998 (11)	pUC ori, *ermB*, *int* and *att*P from T12 bacteriophage, MCS with P$_{lacZ}$_*lacZα*
pEC3103	p7INT.1	Erm^R	This study	pUC ori, *ermB*, *int* and *att*P from T12 bacteriophage, MCS with P$_{lac(Eco)}$_*mrfp*
**Gene deletion plasmids**				
pEC3135	pERASE	Erm^R	This study	pUC ori, MCS with P$_{lac(Eco)}$_*mrfp*, *pheS** under control of P$_{veg}$, *ermB*
pEC3136	pERASE-*ΔsagA*	Erm^R	This study	pUC ori, *pheS** under control of P*veg*, *ermB*, *sagA* flanking regions
**Reporter plasmids**				
pEC2858	pSpy1C-P$_{23}$_*ffluc*	Cm^R	This study	*ffluc* reporter under control of P$_{23}$, *cat*, pSH71 replicon
pEC2860	pSpy1C-P$_{veg}$_*ffluc*	Cm^R	This study	*ffluc* reporter under control of P$_{veg}$, *cat*, pSH71 replicon
pEC2861	pSpy1C-P$_{gyrA}$_*ffluc*	Cm^R	This study	*ffluc* reporter under control of P$_{gyrA}$, *cat*, pSH71 replicon

Code	Designation	Marker	Reference	Features
pEC3105	pSpy1C-P$_{xylS2}$_*ffluc*	Cm^R	This study	*ffluc* reporter under control of P$_{xylS2}$, *cat*, pSH71 replicon
pEC3106	pSpy1C-P$_{veg(1)}$_*ffluc*	Cm^R	This study	*ffluc* reporter under control of P$_{veg(1)}$, *cat*, pSH71 replicon
pEC3107	pSpy1C-P$_{veg(2)}$_ *ffluc*	Cm^R	This study	*ffluc* reporter under control of P$_{veg(2)}$, *cat*, pSH71 replicon
pEC3108	pSpy1C-P$_{veg(3)}$_ *ffluc*	Cm^R	This study	*ffluc* reporter under control of P$_{veg(3)}$, *cat*, pSH71 replicon
pEC2862	pSpy1C-*tetR*-P$_{tet}$_*ffluc*	Cm^R	This study	*ffluc* reporter under control of tetracycline-inducible cassette *tetR*-P$_{Tet}$, *cat*, pSH71 replicon
pEC2863	pSpy1C-P$_{gyrA(Spy)}$_*xylR*-P$_{xylS2}$_*ffluc*	Cm^R	This study	*ffluc* reporter under control of xylose-inducible cassette P$_{gyrA(Spy)}$_*xylR*-P$_{xylS2}$, *cat*, pSH71 replicon
pEC3005	pSpy1C-P$_{nisRK}$_*nisRK*-P$_{nisA}$_*ffluc*	Cm^R	This study	*ffluc* reporter under control of nisin-inducible cassette P$_{nisRK}$_*nisRK*-P$_{nisA}$, *cat*, pSH71 replicon
pEC3006	pSpy1C-P$_{Zn}$_*ffluc*	Cm^R	This study	*ffluc* reporter under control of zinc-inducible promoter P$_{czcD}$, *cat*, pSH71 replicon
pEC3007	pSpy1C-P$_{tre}$_*ffluc*	Cm^R	This study	*ffluc* reporter under control of zinc-inducible promoter P$_{SPy_2097}$, *cat*, pSH71 replicon
pEC3020	pSpy1C-PF6_*lacI*-P$_{lac(Spn)}$_*ffluc*	Cm^R	This study	*ffluc* reporter under control of IPTG-inducible promoter P$_{lac(Spn)}$, *lacI* under control of P$_{F6}$, *cat*, pSH71 replicon
pEC3081	pSpy1C-P$_{Cu}$_*ffluc*	Cm^R	This study	*ffluc* reporter under control of copper-inducible promoter P$_{copA}$, *cat*, pSH71 replicon
pEC3080	pSpy1C-P$_{Mn}$_*ffluc*	Cm^R	This study	*ffluc* reporter under control of manganese-inducible promoter P$_{mntE}$, *cat*, pSH71 replicon
pEC3109	pSpy1C-P$_{lac(1)}$_*ffluc*	Cm^R	This study	*ffluc* reporter under control of IPTG-inducible promoter P$_{lac(1)}$, *cat*, pSH71 replicon
pEC3110	pSpy1C-P$_{lac(2)}$_*ffluc*	Cm^R	This study	*ffluc* reporter under control of IPTG-inducible promoter P$_{lac(2)}$, *cat*, pSH71 replicon
pEC3111	pSpy1C-P$_{lac(3)}$_*ffluc*	Cm^R	This study	*ffluc* reporter under control of IPTG-inducible promoter P$_{lac(t3)}$, *cat*, pSH71 replicon
pEC3104	pSpy1C- P$_{gyrA(Sag)}$-*ermBL_ermB'~ffluc*	Erm^R	This study	*ffluc* reporter under control of erythromycin-inducible riboswitch P$_{gyrA(Sag)}$-*ermBL_ermB'*, *cat*, pSH71 replicon
pEC2826	pUC19-*mKate2~ssrA*	Amp^R	This study	*bla*, *mKate2* fused to native *ssrA* tag (codon optimized for *S. pyogenes*), pUC ori
pEC2827	pUC19-*mNeongreen~ssrA*	Amp^R	This study	*bla*, *mKate2* fused to native *ssrA* tag (codon optimized for *S. pyogenes*), pUC ori

Code	Designation	Marker	Reference	Features
pEC2828	p7INT-*mKate2~ssrA*	Erm^R	This study	pUC ori, *ermB*, *int* and *att*P from T12 bacteriophage, *mKate2* fused to native *ssrA* tag
pEC3137	p7INT-*mKate2~*ssrA*	Erm^R	This study	pUC ori, *ermB*, *int* and *att*P from T12 bacteriophage, *mKate2* fused to native *ssrA* tag with a preceding stop codon
pEC2829	p7INT-*mNeongreen~ssrA*	Erm^R	This study	pUC ori, *ermB*, *int* and *att*P from T12 bacteriophage, *mNeongreen* fused to native *ssrA* tag
pEC2968	p7INT-*mNeongreen~*ssrA*	Erm^R	This study	pUC ori, *ermB*, *int* and *att*P from T12 bacteriophage, *mNeongreen* fused to native *ssrA* tag with a preceding stop codon
pEC2931	p7INT-P_{23}_*mNeongreen ~ssrA*	Erm^R	This study	pUC ori, *ermB*, *int* and *att*P from T12 bacteriophage, *mNeongreen* fused to native *ssrA* tag under control of P_{23}
pEC2934	p7INT-P_{23}_*mKate2~*ssrA*	Erm^R	This study	pUC ori, *ermB*, *int* and *att*P from T12 bacteriophage, *mKate2* fused to native *ssrA* tag with a preceding stop codon under control of P_{23}
pEC2936	p7INT-P_{23}_*mNeongreen~* ssrA*	Erm^R	This study	pUC ori, *ermB*, *int* and *att*P from T12 bacteriophage, *mNeongreen* fused to native *ssrA* tag with a preceding stop codon under control of P_{23}
pEC2935	p7INT-P_{23}_*mNeongreen~ ssrA*(LDDmut)	Erm^R	This study	pUC ori, *ermB*, *int* and *att*P from T12 bacteriophage, *mNeongreen* fused to native *ssrA* tag with introduced LDD mutation under control of P_{23}
pEC2938	p7INT-P_{23}_*mNeongreen~ ssrA*(ASVmut)	Erm^R	This study	pUC ori, *ermB*, *int* and *att*P from T12 bacteriophage, *mNeongreen* fused to native *ssrA* tag with introduced ASV mutation under control of P_{23}
pEC3115	pSpy0K6-P_{23}_*mKate2~*ssrA*	Kan^R	This study	pUC ori, *aph(3')-IIIa*, flanking regions for allelic replacement of *SPy_1078*, *mKate2* fused to native *ssrA* tag with a preceding stop codon under control of P_{23}
pEC3116	pSpy0K6-*mKate2~*ssrA*	KanR	This study	pUC ori, *aph(3')-IIIa*, flanking regions for allelic replacement of *SPy_1078*, *mKate2* fused to native *ssrA* tag with a preceding stop codon
pEC2939	pSpy0C4-P_{23}_*mKate2~*ssrA*	Cm^R	This study	pUC ori, *cat*, flanking regions for allelic replacement of *sagB*, *mKate2* fused to native *ssrA* tag with a preceding stop codon under control of P_{23}
pEC3114	pSpy0C4-*mKate2~*ssrA*	Cm^R	This study	pUC ori, *cat*, flanking regions for allelic replacement of *sagB*, *mKate2* fused to native *ssrA* tag with a preceding stop codon
		**Other**		
pEC3079	p7INT-P_{veg}_*lacI*	Erm^R	This study	pUC ori, *ermB*, *int* and *att*P from T12 bacteriophage, *lacI* under control of P_{veg}

Supplementary Table 4. Information on the DNA sequence of the reporters, promoters and other genetic parts used in this study. Modifications to the original sequences are highlighted in red. The start codons (ATG) as well as the sequence part of the erythromycin-inducible riboswitch are underlined. The -10 and -35 motifs of the promoter sequences were predicted using BPROM and are highlighted in italics (12). The truncated *ermB'* coding sequence that is translationally fused to the downstream reporter is marked in yellow. For the constitutive promoters, we added the optimized 5'UTR of pLZ12Km2-P*23*R:TA:*ffluc* that is colored in grey. Putative binding sites of regulatory proteins are highlighted in bold. The *ssrA* tag sequence attached to the fluorescent reporter genes is marked in blue. $P_{gyrA(Sag)}$ – P_{gyrA} promoter from *Streptococcus agalactiae*.

Designation	5'-3' DNA sequence
	**Reporter genes**
ffluc	ATGGAAGACGCCAAAAACATAAAGAAAGGCCCGGCGCCATTCTATCCTCTAGAGGATGGAACCGCTGGAGAGCAACTGCATA<br>AGGCTATGAAGAGATACGCCCTGGTTCCTGGAACAATTGCTTTTACAGATGCACATATCGAGGTGAACATCACGTACGCGGAA<br>TACTTCGAAATGTCCGTTCGGTTGGCAGAAGCTATGAAACGATATGGGCTGAATACAAATCACAGAATCGTCGTATGCAGTGA<br>AAACTCTCTTCAATTCTTTATGCCGGTGTTGGGCGCGTTATTTATCGGAGTTGCAGTTGCGCCCGCGAACGACATTTATAATGA<br>ACGTGAATTGCTCAACAGTATGAACATTTCGCAGCCTACCGTAGTGTTTGTTTCCAAAAAGGGGTTGCAAAAAATTTTGAACG<br>TGCAAAAAAAATTACCAATAATCCAGAAAATTATTATCATGGATTCTAAAACGGATTACCAGGGATTTCAGTCGATGTACACG<br>TTCGTCACATCTCATCTACCTCCCGGTTTTAATGAATACGATTTTGTACCAGAGTCCTTTGATCGTGACAAAACAATTGCACTG<br>ATAATGAATTCCTCTGGATCTACTGGGTTACCTAAGGGTGTGGCCCTTCCGCATAGAACTGCCTGCGTCAGATTCTCGCATGCC<br>AGAGATCCTATTTTTGGCAATCAAATCATTCCGGATACTGCGATTTTAAGTGTTGTTCCATTCCATCACGGTTTTGGAATGTTTA<br>CTACACTCGGATATTTGATATGTGGATTTCGAGTCGTCTTAATGTATAGATTTGAAGAAGAGCTGTTTTTACGATCCCTTCAGG<br>ATTACAAAATTCAAAGTGCGTTGCTAGTACCAACCCTATTTTCATTCTTCGCCAAAAGCACTCTGATTGACAAATACGATTTAT<br>CTAATTTACACGAAATTGCTTCTGGGGGCGCACCTCTTTCGAAAGAAGTCGGGGAAGCGGTTGCAAAACGCTTCCATCTTCCA<br>GGGATACGACAAGGATATGGGCTCACTGAGACTACATCAGCTATTCTGATTACACCCGAGGGGGATGATAAACCGGGCGCGG<br>TCGGTAAAGTTGTTCCATTTTTTGAAGCGAAGGTTGTGGATCTGGATACCGGGAAAACGCTGGGCGTTAATCAGAGAGGCGAA<br>TTATGTGTCAGAGGACCTATGATTATGTCCGGTTATGTAAACAATCCGGAAGCGACCAACGCCTTGATTGACAAGGATGGATG<br>GCTACATTCTGGAGACATAGCTTACTGGGACGAAGACGAACACTTCTTCATAGTTGACCGCTTGAAGTCTTTAATTAAATACA<br>AAGGATATCAGGTGGCCCCCGCTGAATTGGAATCGATATTGTTACAACACCCCAACATCTTCGACGCGGGCGTGGCAGGTCTT<br>CCCGACGATGACGCCGGTGAACTTCCCGCCGCCGTTGTTGTTTTGGAGCACGGAAAGACGATGACGGAAAAAGAGATCGTGG<br>ATTACGTCGCCAGTCAAGTAACAACCGCGAAAAAGTTGCGCGGAGGAGTTGTGTTTGTGGACGAAGTACCGAAAGGTCTTACC<br>GGAAAACTCGACGCAAGAAAAATCAGAGAGATCCTCATAAAGGCCAAGAAGGGCGGAAAGTCCAAATTGTAA
mKate2~ssrA (codon-optimized for *S. pyogenes*)	ATGGTTTCAGAACTTATCAAAGAAAACATGCACATGAAACTTTACATGGAAGGTACTGTTAACAACCACCACTTCAAATGTAC<br>TTCAGAAGGTGAAGGTAAACCATACGAAGGTACTCAAACTATGCGTATCAAAGCTGTTGAAGGTGGTCCACTTCCATTCGCTT<br>TCGACATCCTTGCTACTTCATTCATGTACGGTTCAAAAACTTTCATCAACCACACTCAAGGTATCCCAGACTTCTTCAAACAAT<br>CATTCCCAGAAGGTTTCACTTGGGAACGTGTTACTACTTACGAAGACGGTGGTGTTCTTACTGCTACTCAAGACACTTCACTTC<br>AAGACGGTTGTCTTATCTACAACGTTAAAATCCGTGGTGTTAACTTCCCATCAAACGGTCCAGTTATGCAAAAAAAAACTCTTG<br>GTTGGGAAGCTTCAACTGAAACTCTTTACCCAGCTGACGGTGGTCTTGAAGGTCGTGCTGACATGGCTCTTAAACTTGTTGGTG<br>GTGGTCACCTTATCTGTAACCTTAAAACTACTTACCGTTCAAAAAAACCAGCTAAAAACCTTAAAATGCCAGGTGTTTACTACG

Designation	5'-3' DNA sequence
	TTGACCGTCGTCTTGAACGTATCAAAGAAGCTGACAAAGAAACTTACGTTGAACAACACGAAGTTGCTGTTGCTCGTTACTGT<br>GACCTTCCATCAAAACTTGGTCACCGTGCAAAAAATACAAACTCTTACGCTTTAGCTGCCTAA
mNeongreen~ssrA (codon-optimized for *S. pyogenes*)	ATGGTTTCAAAAGGTGAAGAAGACAACATGGCTTCACTTCCAGCTACTCACGAACTTCACATCTTCGGTTCAATCAACGGTGTT<br>GACTTCGACATGGTTGGTCAAGGTACTGGTAACCCAAACGACGGTTACGAAGAACTTAACCTTAAATCAACTAAAGGTGACCT<br>TCAATTCTCACCATGGATCCTTGTTCCACACATCGGTTACGGTTTCCACCAATACCTTCCATACCCAGACGGTATGTCACCATTC<br>CAAGCTGCTATGGTTGACGGTTCAGGTTACCAAGTTCACCGTACTATGCAATTCGAAGACGGTGCTTCACTTACTGTTAACTAC<br>CGTTACACTTACGAAGGTTCACACATCAAAGGTGAAGCTCAAGTTAAAGGTACTGGTTTCCCAGCTGACGGTCCAGTTATGAC<br>TAACTCACTTACTGCTGCTGACTGGTGTCGTTCAAAAAAAACTTACCCAAACGACAAAACTATCATCTCAACTTTCAAATGGTC<br>ATACACTACTGGTAACGGTAAACGTTACCGTTCAACTGCTCGTACTACTTACACTTTCGCTAAACCAATGGCTGCTAACTACCT<br>TAAAAACCAACCAATGTACGTTTTCCGTAAAACTGAACTTAAACACTCAAAAACTGAACTTAACTTCAAAGAATGGCAAAAAG<br>CTTTCACTGACGTTATGGGTATGGACGAACTTTACAAAGCAAAAAATACAAACTCTTACGCTTTAGCTGCCTAA
	**Inducible promoters**
P_{czcD} (P_{Zn})	ATCGGACACTTTCTAAATGT**TGTTCACTA*TTGAACA***GGTGCTCATTTTT*GGTTATAGT*AATTGTATTTTAACTTTTAGATAATAG<br>AATAAAGCATTCTACTATACATATTACCAGTATAACAAAAGGAGAAAATT
P_{MntE} (P_{Mn})	CATCTCACCTACTTCCGTATTGTCTTTATCTTAATGGTAGGCCTAGGAGGCTTCCTTAAGTTAGAACTAATTTGGGTTTTGGCTG<br>ATATTGTCAATGGTTTAATGGCACTTCCTAACCTGATTGCCCTCCTAGCTCTCTCGCCAGTTGTTATTTTAGAAACCAAGCATTA<br>CTTTATTAAGTAAACATGACGTTAGTACTACCCAGTTATACCTAGTCTTCTTCTGCCAAGTTCAAGACGCTAGGCTT*TTTTCT*TA<br>AGCCCCTTTTTA*TGGTATAAT*ATAAAGTTGAAATCAAAGGAGTTATT
P_{copA} (P_{Cu})	CATAAGGTCAGTAATAGACCTAAGGTGACAAGTGTTTTTCGAATAGGATGTTTCAAGGGTATCTCCTTAATGTAAATGTTATTT<br>TTATTGCAATAATATTATAGTAAAAACGATTCATTTGTCAATATAAGGATAGAAAAATGAGAAAAGTTATTTA*TTTAC*AAATGT<br>AGTTAAATT*CGTTATAAT*TAATTTACAAACGTAAATATATTGTGAATGAAGGTGACGTT
P_{SPy_2097} (P_{tre})	AGGGTAAATTACCTTTCCATTTGTCTATT*TTTTTA*CAGACAACACC*TTTTATTTT*ACCAAAATAATGGCTTTTTTGGCACAA**ACAA**<br>**CTTGTAGACAAATTTG**CAATCGATTACAATAAAGGTGTCAGTTAGATTCTATCTAAGAATTTGAATATTTCATTTTGACGTTG<br>CTTGTTAAAAGCAACTAGAACAAAGGACTAGGGGATGTCAC
$P_{lac(Spn)}$	GAATTCACTAGTAATTGTGAGGCGGATAACAATTCTCACTCATTCTACAGTTTATTC*TTGACA*TTGCACTGTCCCCCTGG*TATAAT*<br>AACT**AATTGTGAGCGCTCACAATT**AAGATCCCCGGGGCGGCCGCGAATTCAAAAGATCTAAAGAGGAGAAAGGATCT
$P_{lac(1)}$	GAATTCACTAGTAATTGTGAGGCGGATAACAATTCTCACTCATTCTACAGTTTATTC*TTGACA*TTGCACTGTCCCCCTGG*TATAAT*<br>AACTAATTGTGAGCGCTCACAATTAAGATCCCCGGGGCGGCCGCGAATTCAAAAGATCTAAGGAGGAACAT
$P_{lac(2)}$	GAATTCACTAGTAATTGTGAGGCGGATAACAATTCTCACTCATTCTACAGTTTATTC*TTGACA*TTGCACTGTCCCCCTGG*TATAAT*<br>AACTAATTGTGAGCGCTCACAATTAAGATCCCCGGGGCGGCCGCGAATTCAAAAGATCTAAGGAGGAAACAT
$P_{lac(3)}$	GAATTCACTAGTAATTGTGAGGCGGATAACAATTCTCACTCATTCTACAGTTTATTC*TTGACA*TTGCACTGTCCCCCTGG*TATAAT*<br>AACTAATTGTGAGCGCTCACAATTAAGATCCCCGGGGCGGCCGCGAATTCAAAAGATCTAAAGAGGAACAT
$P_{gyrA(Sag)}$_*ermBL*_*ermB'*	GCCTATCATTTTCAATGAAAGAAGTCACTAATAAAATGTGAAAAAATTTGCAAACAAATTGAAATATCATAGAAAACTATTGA<br>AAACGCTGAGTGTC*TTGATA*TTTTTGATACCG*ATTTATCAT*CGCAATTTTACCTAAATTATGGTACAATGTAAGAGGAAGTTAAA<br>TTAGATGCTAAAAATTTGTAATTAAGAAGGAGGGATTCGTCATGTTGGTATTCCAAATGCGTAATGTAGATAAAACATCTACT<br>GTTTTGAAACAGACTAAAAACAGTGATTACGCAGATAAATAAATACGTTAGATTAATTCCTACCAGTGACTAATCTTATGACT<br>TTTTAAACAGATAACTAAAATTACAAACAAATCGTTTAACTTCTGTATTTATTTATAGATGTAATCACTTCAGGAGTGATTACA<br>TGAACAAAAATATAAAATATTCTCAAAAC
	**Constitutive promoters**

Designation	5'-3' DNA sequence
$P_{gyrA(Spy)}$_5'UTR	TTTTTGTGTTGTTTAGTTGCAGTCATTTCTAAACATCTCCTTAATTTTATTAGAGCAAAAGCTCATACGGTCTTATTTTAACATA*T*<br>*TGATA*ATTGTTTGTCATTGTATTCAG*AAAAAT*AATGAAAATAGTTTACTGATAACGCTTCCAATAAGAAAAAAATAAGGAATTTAT<br>GGTATAATGAAATCTAGTATTGACTAGAATACTAGTACTGCAGTGAAATTCAAAAAAATGGCCGGCAGAATTAAATTAAAAA<br>GGGAGGCCAAATATAATGAAAAATATGAATGACAATGATGTTCCGCGGTGGCGGCGATCTAAGGAGGAATAAAAA
P_{23}_5'UTR	CTCGAAAAGCCCTGACAACCCTTGTTCCTAAAAAGGAATAAGCGTTCGGTCAGTAAATAATAGAAATAAAAAATCAGACCTA<br>AGACTG*ATGACA*AAAAGAGAAAATTTTGA*TAAAAT*AGTCTTAGAATTAAATTAAAAAGGGAGGCCAAATATAATGAAAAATAT<br>GAATGACAATGATGTTCCGCGGTGGCGGCGATCTAACCGGTTAATACTAGAATTAAATTAAAAAGGGAGGCCAAATATAATG<br>AAAAATATGAATGACAATGATGTTCCGCGGTGGCGGCGATCTAAGGAGGAATAAAAA
P_{xylS2}_5'UTR	AGCGAGACCAGCTGAATTCAATTAGATATTTAAAAGTATCATATCTAATATTATAACTAAATTTTCTAAAAAAAAACA*TTGAAA*T<br>AAACATTTATTTTGTA*TATGAT*GAGATAAAGTTAGTTTATTGGATAAACAAACTAACTCAATTAAGATAGTTGATGGATAAACT<br>TGTTCACTTAAATCAGAATTAAATTAAAAAGGGAGGCCAAATATAATGAAAAATATGAATGACAATGATGTTCCGCGGTGGC<br>GGCGATCTAAGGAGGAATAAAAA
P_{veg}_5'UTR	GGGAGTTCTGAGAATTGGTATGCCTTATAAGTCCAATTAACAGTTGAAAACCTGCATAGGAGAGCTATGCGGGTTTTTTATTTT<br>ACATAATGATACATAATTTACCGAAACTTGCGGAACATAATTGAGGAATCATAGAATTTTGTCAAAATAATTTTA*TTGACA*ACG<br>TCTTATTAACGTTGA*TATAAT*TTAAATTTTATTTGACAAAAATGGGCTCGTGTTGTACAATAAATGTAGTTACTAGTACTGCAGT<br>GAAATTCAAAAAAATGGCCGGCAGAATTAAATTAAAAAGGGAGGCCAAATATAATGAAAAATATGAATGACAATGATGTTCC<br>GCGGTGGCGGCGATCTAAGGAGGAATAAAAA
$P_{veg(1)}$_5'UTR	GGGAGTTCTGAGAATTGGTATGCCTTATAAGTCCAATTAACAGTTGAAAACCTGCATAGGAGAGCTATGCGGGTTTTTTATTTT<br>ACATAATGATACATAATTTACCGAAACTTGCGGAACATAATTGAGGAATCATAGAATTTTGTCAAAATAATTTTA*TTGACA*ACG<br>TCTTATTAACATGGA*TATAAT*TTAAATTTTATTTGACAAAAATGGGCTCGTGTTGTACAATAAATGTAGTTACTAGTACTGCAGT<br>GAAATTCAAAAAAATGGCCGGCAGAATTAAATTAAAAAGGGAGGCCAAATATAATGAAAAATATGAATGACAATGATGTTCC<br>GCGGTGGCGGCGATCTAAGGAGGAATAAAAA
$P_{veg(2)}$_5'UTR	GGGAGTTCTGAGAATTGGTATGCCTTATAAGTCCAATTAACAGTTGAAAACCTGCATAGGAGAGCTATGCGGGTTTTTTATTTT<br>ACATAATGATACATAATTTACCGAAACTTGCGGAACATAATTGAGGAATCATAGAATTTTGTCAAAATAATTTTA*TTGACA*AAA<br>CGTCTTATTAACGTTGA*TATAAT*TTAAATTTTATTTGACAAAAATGGGCTCGTGTTGTACAATAAATGTAGTTACTAGTACTGCA<br>GTGAAATTCAAAAAAATGGCCGGCAGAATTAAATTAAAAAGGGAGGCCAAATATAATGAAAAATATGAATGACAATGATGTT<br>CCGCGGTGGCGGCGATCTAAGGAGGAATAAAAA
$P_{veg(3)}$_5'UTR	GGGAGTTCTGAGAATTGGTATGCCTTATAAGTCCAATTAACAGTTGAAAACCTGCATAGGAGAGCTATGCGGGTTTTTTATTTT<br>ACATAATGATACATAATTTACCGAAACTTGCGGAACATAATTGAGGAATCATAGAATTTTGTCAAAATAATTTT*ATTGACA*ACG<br>TC**ATTAACGTTGA*TATAAT*TTAAATTTTATTTGACAAAAATGGGCTCGTGTTGTACAATAAATGTAGTTACTAGTACTGCAGT<br>GAAATTCAAAAAAATGGCCGGCAGAATTAAATTAAAAAGGGAGGCCAAATATAATGAAAAATATGAATGACAATGATGTTCC<br>GCGGTGGCGGCGATCTAAGGAGGAATAAAAA
**Other**	
P_{f6}_*lacI*	GACTGTACGTCATCAGAAGTTTCAGCGACCCTAGATCCTGCTCGAAGATTTCAGC*TTGACA*TTGCACTGTCCCCCTGG*TATAAT*A<br>ACTATACATGCAAGGATCTAAATAAATAAGGAGGAAAATTAATGAAACCAGTCACCCTTTACGATGTCGCAGAATACGCAGG<br>AGTCTCTTACCAAACAGTCAGTCGTGTAGTCAATCAGGCTAGTCATGTATCAGCAAAAACTCGTGAAAAGGTTGAAGCTGCAA<br>TGGCTGAACTTAATTACATCCCAAATCGTGTTGCCCAACAGTTGGCTGGTAAACAAAGTTTGCTTATCGGAGTTGCTACCTCTT<br>CATTGGCACTTCATGCCCCTTCTCAAATTGTTGCCGCTATCAAATCTCGTGCAGATCAATTGGGTGCAAGTGTTGTCGTATCTAT<br>GGTTGAACGTAGTGGAGTCGAAGCCTGTAAGGCAGCCGTCCACAATTTATTGGCTCAACGTGTATCTGGTTTAATTATCAATTA<br>CCCATTGGATGACCAAGATGCCATTGCTGTCGAAGCTGCATGTACCAATGTACCAGCATTATTTTGGATGTTTCAGATCAAAC

Designation	5'-3' DNA sequence
	ACCTATCAATTCAATCATCTTTAGTCATGAAGATGGTACTCGTTTGGGAGTCGAACATTTGGTAGCTCTTGGTCACCAACAGAT<br>TGCTCTTTTAGCAGGACCACTTAGTTCTGTTTCAGCCCGTCTTCGTTTAGCTGGTTGGCACAAATACTTAACTCGTAATCAAATT<br>CAGCCTATCGCTGAACGTGAGGGTGATTGGTCTGCAATGTCAGGATTTCAACAGACAATGCAAATGTTGAATGAAGGTATTGT<br>ACCAACTGCAATGTTGGTTGCCAATGACCAAATGGCCCTTGGAGCTATGCGTGCAATTACCGAATCAGGTTTGCGTGTTGGAG<br>CTGATATTAGTGTTGTCGGTTACGATGATACAGAAGATTCAAGTTGTTACATTCCACCTTTGACAACTATCAAGCAAGATTTTC<br>GTTTGCTTGGACAGACAAGTGTTGATCGTTTATTGCAACTTTCTCAGGGTCAGGCTGTCAAGGGAAATCAGCTTTTACCTGTAT<br>CTCTTGTTAAGCGTAAGACCACATTAGCACCAAATACTCAAACCGCCTCACCTCGTGCCTTGGCAGATAGTCTTATGCAGCTTG<br>CACGTCAGGTCTCTCGTTTGGAAAGTGGACAATAA
P_{veg}_*lacI*	GCGGGTTTTTTATTTTACATAATGATACATAATTTACCGAAACTTGCGGAACATAATTGAGGAATCATAGAATTTTGTCAAAAT<br>AATTTTA*TTGACA*ACGTCTTATTAACGTTGA*TATAAT*TTAAATTTTATTTGACAAAAATGGGCTCGTGTTGTACAATAAATGTAGT<br>TAAAGGAGGAATAAAAAATGAAACCAGTCACCCTTTACGATGTCGCAGAATACGCAGGAGTCTCTTACCAAACAGTCAGTCG<br>TGTAGTCAATCAGGCTAGTCATGTATCAGCAAAAACTCGTGAAAAGGTTGAAGCTGCAATGGCTGAACTTAATTACATCCCAA<br>ATCGTGTTGCCCAACAGTTGGCTGGTAAACAAAGTTTGCTTATCGGAGTTGCTACCTCTTCATTGGCACTTCATGCCCCTTCTCA<br>AATTGTTGCCGCTATCAAATCTCGTGCAGATCAATTGGGTGCAAGTGTTGTCGTATCTATGGTTGAACGTAGTGGAGTCGAAGC<br>CTGTAAGGCAGCCGTCCACAATTTATTGGCTCAACGTGTATCTGGTTTAATTATCAATTACCCATTGGATGACCAAGATGCCAT<br>TGCTGTCGAAGCTGCATGTACCAATGTACCAGCATTATTTTTGGATGTTTCAGATCAAACACCTATCAATTCAATCATCTTTAG<br>TCATGAAGATGGTACTCGTTTGGGAGTCGAACATTTGGTAGCTCTTGGTCACCAACAGATTGCTCTTTTAGCAGGACCACTTAG<br>TTCTGTTTCAGCCCGTCTTCGTTTAGCTGGTTGGCACAAATACTTAACTCGTAATCAAATTCAGCCTATCGCTGAACGTGAGGG<br>TGATTGGTCTGCAATGTCAGGATTTCAACAGACAATGCAAATGTTGAATGAAGGTATTGTACCAACTGCAATGTTGGTTGCCA<br>ATGACCAAATGGCCCTTGGAGCTATGCGTGCAATTACCGAATCAGGTTTGCGTGTTGGAGCTGATATTAGTGTTGTCGGTTACG<br>ATGATACAGAAGATTCAAGTTGTTACATTCCACCTTTGACAACTATCAAGCAAGATTTTCGTTTGCTTGGACAGACAAGTGTTG<br>ATCGTTTATTGCAACTTTCTCAGGGTCAGGCTGTCAAGGGAAATCAGCTTTTACCTGTATCTCTTGTTAAGCGTAAGACCACAT<br>TAGCACCAAATACTCAAACCGCCTCACCTCGTGCCTTGGCAGATAGTCTTATGCAGCTTGCACGTCAGGTCTCTCGTTTGGAAA<br>GTGGACAATAA
*pheS**	ATGGACCTTCAAGCTCAATTGGAAGAACTTAAAACTAAAACCTTGGAAACTCTTCAAAGTCTTACTGGTAACCATACAAAAGA<br>ATTGCAAGATTTGCGTGTTGCAGTTCTTGGTAAGAAGGGTTCACTTACTGATTTGCTTAAAGGTCTTAAGGATTTGTCAAACGA<br>TTTGCGTCCTGTGGTAGGCAAACAAGTAAACGAAGTTCGTGACTTATTGACAAAAGCTTTCGAAGAGCAAGCTAAGATCGTTG<br>AAGCCGCTAAAATTCAAGCTCAATTGGATGCTGAATCAATCGACGTTACTCTTCCTGGACGTCAAATGACTCTGGGGCATCGT<br>CACGTTCTTACTCAAACATCAGAAGAAATCGAAGACATCTTCCTTGGTATGGGTTTCCAAATTGTAGATGGTTTCGAAGTTGAA<br>AAAGATTATTATAACTTCGAACGTATGAACCTTCCAAAAGACCACCCTGCTCGTGACATGCAAGACACTTTCTACATTACTGA<br>AGAGATCTTGTTACGTACTCACACTTCACCAGTACAAGCTCGTACTCTTGATCAACACGACTTCTCTAAGGGCCCTTTAAAAAT<br>GGTTTCACCAGGCCGTGTATTCCGCCGCGACACTGACGACGCTACTCACTCACACCAATTCCACCAAATCGAAGGTCTTGTAGT<br>TGGCAAAAATATTTCAATGGGTGACTTGAAAGGCACTTTGGAAATGATCATTAAAAAAATGTTCGGTGACGAACGTAGCATCC<br>GTCTTCGCCCTTCTTATTTCCCTTTCTCAGAACCATCAGTAGAAGTTGACGTATCATGTTTCAAATGTGGAGGTAAAGGCTGTA<br>ACGTTTGTAAAAAAACTGGTTGGATCGAAATCTTGGGTGCTGGTATGGTTCACCCAAGTGTTCTTGAAATGTCAGGTGTTGATG<br>CAAAAGAGTACTCAGGTTTCGGTTTCGGCTTGGGACAAGAGCGTATCGCTATGTTGCGCTACGGAATTAACGATATTCGTGGC<br>TTCTACCAAGGCGACCAACGTTTCTCAGAACAATTTAACTAA

Supplementary Table 5. Details of the construction of reporter plasmids featuring different constitutive promoters upstream of the firefly luciferase reporter *ffluc*. The first column ('Plasmid') indicates the name of the plasmid that was constructed. The second column ('Assembly method') defines which assembly strategy was used. The amplicons or fragments required for plasmid assembly were obtained by PCR and the amplicons, respective oligonucleotides and the templates used are specified in the following columns. Abbreviations: TA = Toxin-Antitoxin

Plasmid	Assembly Method	Amplicon	Oligo Code	Template
pSpy1C-P_{23}_*ffluc*	Golden Gate Assembly	P_{23} promoter	OLEC10026	pIB166
			OLEC10027	
		ffluc reporter (+ Cloning overhangs)	OLEC10182	pLZ12Km2-P23R:TA:*ffluc*
			OLEC10024	
pSpy1C-P_{veg}_*ffluc*	Golden Gate Assembly	P_{veg} promoter	OLEC9749	*Bacillus subtilis* genomic DNA
			OLEC9750	
		P_{veg} promoter (+ *Eco*31I recognition sites)	OLEC10026	P_{veg} amplicon
			OLEC10027	
		ffluc reporter	OLEC9562	pLZ12Km2-P23R:TA:*ffluc*
			OLEC9563	
		ffluc reporter (+ Cloning overhangs)	OLEC10182	*ffluc* amplicon
			OLEC10024	
pSpy1C-P_{gyrA}_*ffluc*	Golden Gate Assembly	P_{gyrA} promoter	OLEC9591	*Streptococcus pyogenes* SF370 genomic DNA
			OLEC9592	
		P_{gyrA} promoter (+ *Eco*31I recognition sites)	OLEC10026	P_{gyrA} amplicon
			OLEC10027	
		ffluc reporter	OLEC9562	pLZ12Km2-P23R:TA:*ffluc*
			OLEC9563	
		ffluc reporter (+ Cloning overhangs)	OLEC10182	*ffluc* amplicon
			OLEC10024	
pSpy1C-P_{xylS2}_*ffluc*	Gibson Assembly	P_{xylS2} promoter	OLEC14584	gene synthesis fragment (GenScript services)
			OLEC14585	
		pSpy1C-*ffluc*	OLEC14586	pSpy1C-*ffluc*
			OLEC14587	
pSpy1C-$P_{veg(1)}$_*ffluc*	Gibson Assembly	PCR mutagenesis	OLEC14589	pSpy1C-P_{veg}_*ffluc*
			OLEC14590	
pSpy1C-$P_{veg(2)}$_*ffluc*	Gibson Assembly	PCR mutagenesis	OLEC14591	pSpy1C-P_{veg}_*ffluc*
			OLEC14592	
pSpy1C-$P_{veg(3)}$_*ffluc*	Gibson Assembly	PCR mutagenesis	OLEC14593	pSpy1C-P_{veg}_*ffluc*
			OLEC14594	

Supplementary Table 6. Details of the construction of reporter plasmids featuring different inducible promoters upstream of the firefly luciferase reporter *ffluc*. The first column ('Plasmid') indicates the name of the plasmid that was constructed. The second column ('Assembly method') defines which assembly strategy was used to ligate the fragments together to create the plasmid. The amplicons or fragments required for plasmid assembly were obtained by PCR and the amplicons, respective oligonucleotides and the templates used are specified in following columns. Symbols: (~) indicates translational fusions; TA = Toxin-Antitoxin

Plasmid	Assembly Method	Amplicon	Oligo Name	Template
pSpy1C-*nisRK*-P_{nisA}_*ffluc*	Gibson Assembly	P_{nisRK}_*nisRK*	OLEC13447	pMSP3535
			OLEC13448	
		P_{nisA} promoter	OLEC13445	pMSP3535
			OLEC13446	
		pSpy1C-*ffluc*	OLEC13449	pSpy1C-P_{23}_*ffluc*
			OLEC13450	
pSpy1C-*tetR*-P_{tet}_*ffluc*	Golden Gate Assembly	*tetR*-P_{tet}	OLEC9595	pEU8517
			OLEC9596	
		tetR-P_{tet} (+*Eco*31I recognition sites)	OLEC10026	*tetR*-P_{tet} amplicon
			OLEC10027	
		ffluc reporter	OLEC10024	pLZ12Km2-P23R:TA:*ffluc*
			OLEC10182	
pSpy1C-P_{tre}_*ffluc*	Gibson Assembly	P_{SPy_2097} (P_{tre})	OLEC13456	*Streptococcus pyogenes* SF370 genomic DNA
			OLEC13457	
		pSpy1C-*ffluc*	OLEC13458	pSpy1C-P_{23}_ffluc
			OLEC13459	
pSpy1C-P_{Zn}_*ffluc*	Gibson Assembly	P_{czcD} (P_{Zn})	OLEC13460	*Streptococcus pyogenes* SF370 genomic DNA
			OLEC13461	
		pSpy1C-*ffluc*	OLEC13462	pSpy1C-P_{23}_*ffluc*
			OLEC13463	
pSpy1C-P_{Cu}_*ffluc*	Gibson Assembly	P_{copA} (P_{Cu})	OLEC14363	*Streptococcus pyogenes* SF370 genomic DNA
			OLEC14364	
		pSpy1C-*ffluc*	OLEC14357	pSpy1C-P_{Zn}_*ffluc*
			OLEC14358	
pSpy1C-P_{Mn}_*ffluc*	Gibson Assembly	P_{mntE} (P_{Mn})	OLEC14359	*Streptococcus pyogenes* SF370 genomic DNA
			OLEC14360	
		pSpy1C-*ffluc*	OLEC14357	pSpy1C-P_{Zn}_*ffluc*
			OLEC14358	
pSpy1C-P_{F6}_*lacI*-$P_{lac(Spn)}$_*ffluc*	Gibson Assembly	P_{F6}_*lacI*	OLEC13464	pPEPY-P_{F6}_*lacI*
			OLEC13465	
		$P_{lac(Spn)}$ promoter	OLEC13466	pJW102-PL-dCas9

Plasmid	Assembly Method	Amplicon	Oligo Name	Template
			OLEC13467	
		pSpy1C-*ffluc*	OLEC13468	pSpy1C-P$_{23}$_*ffluc*
			OLEC13469	
p7INT-P$_{veg}$_*lacI*	Gibson Assembly	P$_{veg}$ promoter	OLEC14312	pSpy1C-P$_{veg}$_*ffluc*
			OLEC14313	
		lacI repressor	OLEC14314	pSpy1C-P$_{F6}$_*lacI*-P$_{lac}$_*ffluc*
			OLEC14315	
		p7INT backbone	OLEC14316	p7INT
			OLEC14317	
pSpy1C-P$_{lac(1)}$_*ffluc*	Gibson Assembly	P$_{lac(1)}$	OLEC14548	pSpy1C-P$_{F6}$_*lacI*-P*lac*_*ffluc*
			OLEC14549	
		pSpy1C-*ffluc*	OLEC13457	pSpy1C-P$_{Zn}$_*ffluc*
			OLEC13458	
pSpy1C-P$_{lac(2)}$_*ffluc*	Gibson Assembly	P$_{lac(2)}$	OLEC14548	pSpy1C-P$_{F6}$_*lacI*-P*lac*_*ffluc*
			OLEC14550	
		pSpy1C-*ffluc*	OLEC13457	pSpy1C-P$_{Zn}$_*ffluc*
			OLEC13458	
pSpy1C-P$_{lac(3)}$_*ffluc*	Gibson Assembly	P$_{lac(3)}$	OLEC14548	pSpy1C-P$_{F6}$_*lacI*-P*lac*_*ffluc*
			OLEC14551	
		pSpy1C-*ffluc*	OLEC13457	pSpy1C-P$_{Zn}$_*ffluc*
			OLEC13458	
pSpy1C-P$_{gyrA}$_*ermBL-ermB'~ffluc*	Gibson Assembly	P$_{gyrA}$_*ermBL-ermB'*	OLEC14377	pLZ12-P$_{gyrA}$-*ermBL-ermB'*:A2I:*ffluc*
			OLEC14582	
		pSpy1C-*ffluc*	OLEC14583	pSpy1C-P$_{Zn}$_*ffluc*
			OLEC14380	

Supplementary Table 7. Details of the construction of reporter plasmids featuring the fluorescent reporter genes *mKate2* and *mNeongreen*. The first column ('Plasmid') indicates the name of the plasmid that was constructed. The second column ('Assembly method') defines which assembly strategy was used to ligate the fragments together to create the plasmid. The amplicons or fragments required for plasmid assembly were obtained by PCR and the amplicons, respective oligonucleotides and the templates used are specified in following columns. Symbols: (~) indicates translational fusions; (*) indicates stop codon (TAA)

Plasmid	Assembly Method	Amplicon	Oligo Name	Template
p7INT-*mNeongreen-ssrA*	Gibson Assembly	*mNeongreen-ssrA*	OLEC12109	pUC19-*mNeongreen-ssrA*
			OLEC12110	
		mNeongreen-ssrA (+ Cloning overhangs)	OLEC12103	mNeongreen-ssrA amplicon
			OLEC12104	
		p7INT backbone	OLEC12105	p7INT
			OLEC12107	
p7INT-*mKate2~ssrA*	Gibson Assembly	*mKate2~ssrA*	OLEC12108	pUC19-*mKate2~ssrA*
			OLEC12110	
		mKate2~ssrA (+ Cloning overhangs)	OLEC12102	*mKate2~ssrA* amplicon
			OLEC12103	
		p7INT backbone	OLEC12105	p7INT
			OLEC12106	
p7INT-P_{23}_*mNeongreen~ssrA*	Gibson Assembly	P_{23} promoter	OLEC12264	pSpy1C-P_{23}_*ffluc*
			OLEC12262	
		p7INT-*mNeongreen~ssrA*	OLEC12265	p7INT-*mNeongreen~ssrA*
			OLEC12267	
p7INT-P_{23}_*mKate2~ssrA*	Gibson Assembly	P_{23} promoter	OLEC12263	pSpy1C-P_{23}_*ffluc*
			OLEC12264	
		p7INT-*mKate2~ssrA*	OLEC12266	p7INT-*mKate2~ssrA*
			OLEC12267	
pSpy0C4-P_{23}_*mNeongreen~ssrA*	Gibson Assembly	P_{23} promoter	OLEC12261	pSpy1C-P_{23}_*ffluc*
			OLEC12262	
		pSpy0C4-*mNeongreen~ssrA*	OLEC12265	pSpy0C4-*mNeongreen~ssrA*
			OLEC12268	
pSpy0C4-P_{23}_*mKate2~ssrA*	Gibson Assembly	P_{23} promoter	OLEC12261	pSpy1C-P_{23}_*ffluc*
			OLEC12263	
		pSpy0C4-*mKate2~ssrA*	OLEC12266	pSpy0C4-*mKate2~ssrA*
			OLEC12268	
pSpy0K6-*mKate2~*ssrA*	Gibson Assembly	*mKate2~*ssrA*	OLEC14721	p7INT-*mKate2~*ssrA*
			OLEC14718	
		pSpy0K6 backbone	OLEC14719	pSpy0K6

**Plasmid**	**Assembly Method**	**Amplicon**	**Oligo Name**	**Template**
			OLEC14720	
pSpy0K6-P$_{23}$_*mKate2~*ssrA*	Gibson Assembly	P$_{23}$_*mKate2~*ssrA*	OLEC14717	p7INT-P$_{23}$_*mKate2~*ssrA*
			OLEC14718	
		pSpy0K6 backbone	OLEC14719	pSpy0K6
			OLEC14720	

Supplementary Table 8. Initial compositions for the development of an RPMI1640-based chemically defined medium. Specification of the components and their concentrations supplemented to the RPMI4Spy interim media RPMImod version 1 (V1) and RPMImod version 2 (V2). The recipe in the third column of RPMImod V1 resembles the final RPMI4Spy recipe (with 1x Niacinamide and 1x BME vitamin solution).

**Component**	**RPMImod V1**			**RPMImod V2**		
2 M Glucose	7 g/L	7 g/L	7 g/L	7 g/L	7 g/L	7 g/L
100x Nucleobase Mix (2 g/L A, U and G)	1x	1x	1x	1x	1x	1x
10 mM HEPES Buffer, pH 7.4	0.02 mM	0.02 mM	0.02 mM	0.02 mM	0.02 mM	0.02 mM
10 mg/mL Niacinamide (1000x)	1x / 4x	1x / 4x	1x / 4x	1x / 4x	1x / 4x	1x / 4x
50x RPMI1640 Amino Acid Solution (AA)	1x	1x	1x	n.a.	n.a.	n.a.
40 mM Amino Acid mix (lab recipe)	n.a.	n.a.	n.a.	100 µM	100 µM	100 µM
DL-Methionine	n.a.	n.a.	n.a.	100 µM	100 µM	100 µM
100x RPMI Vitamin Solution	1x / without	n.a.	n.a.	1x / without	n.a.	n.a.
100x MEM Vitamin Solution	n.a.	1x / without	n.a.	n.a.	1x / without	n.a.
100x BME Vitamin Solution	n.a.	n.a.	1x / without	n.a.	n.a.	1x / without
RPMI1640 with glutamine, without phenol red	1x	1x	1x	1x	1x	1x

Supplementary Table 9. Transcriptionally silent sites in the genome of *S. pyogenes* SF370. The start indicates the start coordinate of the transcriptionally silent region in the genome of the target strain, while the end respectively marks the last coordinate of this region. The different region types are differentiated as follows: (1) not_expressed_genes: regions annotated as genes including the promoter region upstream, but not expressed above threshold and not within annotated prophage region; (2) not_expressed_intergenic: regions not annotated as genes, not expressed above threshold and not within annotated prophage region. For each region, the average normalized read coverage is listed.

Start	End	Length	Region Type	Average Norm. Coverage
102432	104957	2526	not_expressed_genes	0.016931121523403325
1800683	1803073	2391	not_expressed_genes	0.015151034098748165
958923	961224	2302	not_expressed_genes	0.007905311763261175
1620601	1622746	2146	not_expressed_genes	0.01827871995969429
1166936	1168979	2044	not_expressed_genes	0.012705785045752685
710427	712411	1985	not_expressed_genes	0.010902720300538214
506187	508155	1969	not_expressed_genes	0.018604086811677006
884387	886269	1883	not_expressed_genes	0.0038432372393893974
1281009	1282630	1622	not_expressed_genes	0.0113646590107065
124013	125222	1210	not_expressed_genes	0.01327014526837671
1595608	1596627	1020	not_expressed_genes	0.015906317658427017
447282	448135	854	not_expressed_genes	0.009422351236340117
360546	361116	571	not_expressed_genes	0.015460012804892526
890337	890843	507	not_expressed_genes	0.013401895442700288
1803549	1804033	485	not_expressed_genes	0.012248901120239897
597998	598364	367	not_expressed_genes	0.0076555940892690045
449773	450134	362	not_expressed_genes	0.006159199007649874
1709968	1710315	348	not_expressed_genes	0.016581919802625255
1625956	1626263	308	not_expressed_genes	0.016164889027133717
1755584	1755858	275	not_expressed_genes	0.01799136215746988
971336	971594	259	not_expressed_genes	0.01574077903543419
270237	270596	360	not_expressed_intergenic	0.012492034248109662
1336434	1336658	225	not_expressed_intergenic	0.011190354406972709
1803104	1803326	223	not_expressed_intergenic	0.007972241466287083
390806	391013	208	not_expressed_intergenic	0.015926178855598355
263746	263953	208	not_expressed_intergenic	0.016675393034894626
1665879	1666082	204	not_expressed_intergenic	0.013026521027912227
153837	154037	201	not_expressed_intergenic	0.009320644543472298
396287	396476	190	not_expressed_intergenic	0.013234815957621913
263452	263638	187	not_expressed_intergenic	0.01262922746677333
361133	361289	157	not_expressed_intergenic	0.010517298835780842
168047	168202	156	not_expressed_intergenic	0.01373345329790649
597845	597997	153	not_expressed_intergenic	0.01342972821309218

Start	End	Length	Region Type	Average Norm. Coverage
450135	450281	147	not_expressed_intergenic	0.0063777400406086595
422383	422526	144	not_expressed_intergenic	0.013825369110658483
1755440	1755573	134	not_expressed_intergenic	0.016500749179115624
429323	429435	113	not_expressed_intergenic	0.015345124650857998
662817	662924	108	not_expressed_intergenic	0.016308723798256586
1493100	1493200	101	not_expressed_intergenic	0.009454247295459012

Supplementary Table 10. Transcriptionally silent sites in the genome of *S. pyogenes* M1T1 5448. The start indicates the start coordinate of the non-transcribed region in the genome of the target strain, while the end respectively marks the last coordinate of this region. The different region types are differentiated as follows: (1) not_expressed_genes: regions annotated as genes including the promoter region upstream, but not expressed above threshold and not within annotated prophage region; (2) not_expressed_intergenic: regions not annotated as genes, not expressed above threshold and not within annotated prophage region. For each region, the average normalized read coverage is listed.

Start	End	Length	Region type	Average Norm. Coverage
96786	99311	2526	not_expressed_genes	0.015717676329681903
1140397	1142440	2044	not_expressed_genes	0.018527613542344997
820392	821774	1383	not_expressed_genes	0.008018699105305255
466315	467168	854	not_expressed_genes	0.012071768732113186
377155	377899	745	not_expressed_genes	0.019622565568269714
576128	576494	367	not_expressed_genes	0.01062323039617106
149480	149934	455	not_expressed_intergenic	0.011283169309065798
289281	289621	341	not_expressed_intergenic	0.011835205232508523
575950	576127	178	not_expressed_intergenic	0.010403985602063976
441416	441559	144	not_expressed_intergenic	0.01024269522951294
1215297	1215429	133	not_expressed_intergenic	0.011895730748578531
448352	448475	124	not_expressed_intergenic	0.008256789618203633
1829716	1829837	122	not_expressed_intergenic	0.01747821937253233
415279	415389	111	not_expressed_intergenic	0.015494377002127614

Supplementary Table 11. Identification of transcriptionally silent sites showing homology between *S. pyogenes* SF370 and M1T1 5448. The start indicates the start coordinate of the transcriptionally silent region in the genome of the target strain, while the end respectively marks the last coordinate of this region. While the target strain shows the strain in which the transcriptionally silent region was identified, the reference strain is the strain to be compared against. For each region, the average read coverage is listed. Regions that are homologous between reference and target strain are indicated by 'true', while non-homologous sequences are indicated by 'false'. Conserved expression of the regions is marked by 'true', while differences in expression are indicated by 'false'.

Target Strain	Region Start	Region End	Target_Cov	Ref Strain	Ref Start	Ref End	Alignment Score Norm.	Homolog	Ref Cov	Expression Conserved
**SF370**	102431	104956	0.01692441878329113	5448	96786	99311	100.0	TRUE	0.01571145397167332	TRUE
**SF370**	1800682	1803072	0.015144697405273156	5448	1777827	1780217	100.0	TRUE	0.11822973164217583	FALSE
**SF370**	958922	961223	0.007901877657369228	5448	894428	896729	100.0	TRUE	0.02875329957213702	FALSE
**SF370**	1620600	1622745	0.01827020238282584	5448	243742	245887	64.4	FALSE	0.03202424476452032	FALSE
**SF370**	1166935	1168978	0.012699568908254764	5448	1140397	1142440	100.0	TRUE	0.018518549152157948	TRUE
**SF370**	710426	712410	0.010897227746230639	5448	688557	690541	99.9	TRUE	0.029349018400147385	FALSE
**SF370**	506186	508154	0.018594638316597436	5448	525219	527187	99.9	TRUE	0.06169221625124542	FALSE
**SF370**	884386	886268	0.0038411962212059722	5448	819892	821774	99.9	TRUE	0.012549518468624307	TRUE
**SF370**	1281008	1282629	0.011357652439183253	5448	1254343	1255964	100.0	TRUE	0.02955715063034292	FALSE
**SF370**	124012	125221	0.013259178206171441	5448	118367	119576	100.0	TRUE	0.07616098714840958	FALSE
**SF370**	1595607	1596626	0.015890723229350128	5448	269867	270886	66.7	FALSE	0.037698408991480144	FALSE
**SF370**	447281	448134	0.0094113180381711	5448	466315	467168	100.0	TRUE	0.01205763317153694	TRUE
**SF370**	360545	361115	0.015432937475987287	5448	379579	380149	100.0	TRUE	0.020822473022778263	FALSE
**SF370**	890336	890842	0.013375461723878395	5448	825842	826348	100.0	TRUE	0.03791765663422581	FALSE
**SF370**	1803548	1804032	0.0122236456540126	5448	1780693	1781177	100.0	TRUE	0.08617077108755264	FALSE
**SF370**	597997	598363	0.00763473415987045	5448	576128	576494	100.0	TRUE	0.010594284264301384	TRUE
**SF370**	449772	450133	0.006142184645750289	5448	468806	469167	100.0	TRUE	0.018761988756782807	TRUE
**SF370**	1709967	1710314	0.016534270607790124	5448	1700641	1700988	100.0	TRUE	0.04497696285272824	FALSE
**SF370**	1625955	1626262	0.01611240562120146	5448	240225	240532	63.0	FALSE	0.037063036076846925	FALSE
**SF370**	1755583	1755857	0.01792593902235181	5448	1746257	1746531	100.0	TRUE	0.06719254670818217	FALSE
**SF370**	971335	971593	0.015680003826803162	5448	906841	907099	100.0	TRUE	0.03363872223879218	FALSE

Target Strain	Region Start	Region End	Target_Cov	Ref Strain	Ref Start	Ref End	Alignment Score Norm.	Homolog	Ref Cov	Expression Conserved
**SF370**	270236	270595	0.012457334152976025	5448	289262	289621	100.0	TRUE	0.012332801130031166	TRUE
**SF370**	1336433	1336657	0.011140619498497276	5448	1308505	1308729	100.0	TRUE	0.03328478649100947	FALSE
**SF370**	1803103	1803325	0.007936491504554855	5448	1780248	1780470	100.0	TRUE	0.027655154604246012	FALSE
**SF370**	390805	391012	0.01584961068802336	5448	409839	410046	100.0	TRUE	0.020757886124931547	FALSE
**SF370**	263745	263952	0.016595222876073016	5448	1597680	1597887	60.6	FALSE	0.04564855355173125	FALSE
**SF370**	1665878	1666081	0.012962665532677364	5448	200406	200609	66.7	FALSE	0.023479065415544193	FALSE
**SF370**	153836	154036	0.009274273177584376	5448	148232	148403	79.6	FALSE	0.026017710789881875	FALSE
**SF370**	396286	396475	0.013165159031529166	5448	415320	415509	100.0	TRUE	0.02083540296653639	FALSE
**SF370**	263451	263637	0.012561691491015183	5448	1597995	1598181	62.0	FALSE	0.04077800987449826	FALSE
**SF370**	361132	361288	0.010450309671221729	5448	380166	380322	100.0	TRUE	0.033433110818197664	FALSE
**SF370**	168046	168201	0.013645418340868628	5448	162457	162612	99.4	TRUE	0.024988144409190127	FALSE
**SF370**	597844	597996	0.013341952211699419	5448	575975	576127	100.0	TRUE	0.009700551619978964	TRUE
**SF370**	450134	450280	0.006334354053937852	5448	469168	469314	100.0	TRUE	0.02209334145262602	FALSE
**SF370**	422382	422525	0.013729359602945577	5448	441416	441559	100.0	TRUE	0.010171565401530212	TRUE
**SF370**	1755439	1755572	0.016377609259868493	5448	1746113	1746246	100.0	TRUE	0.04063210108949035	FALSE
**SF370**	429322	429434	0.01520932708757607	5448	448356	448468	100.0	TRUE	0.00783594089547085	TRUE
**SF370**	662816	662923	0.016157717096420877	5448	640947	641054	100.0	TRUE	0.08769146438524542	FALSE
**SF370**	1493099	1493199	0.009360640886593082	5448	1505973	1506073	100.0	TRUE	0.011315965669465576	TRUE
**5448**	96785	99310	0.01571145397167332	SF370	102432	104957	100.0	TRUE	0.01692441878329113	TRUE
**5448**	1140396	1142439	0.018518549152157948	SF370	1166936	1168979	100.0	TRUE	0.012699568908254764	TRUE
**5448**	820391	821773	0.008012901058229835	SF370	884887	886269	99.9	TRUE	0.003157348477179127	TRUE
**5448**	466314	467167	0.01205763317153694	SF370	447282	448135	100.0	TRUE	0.0094113180381711	TRUE
**5448**	377154	377898	0.019596226554084117	SF370	358122	358866	100.0	TRUE	0.021847510452131447	FALSE
**5448**	576127	576493	0.010594284264301384	SF370	597998	598364	100.0	TRUE	0.00763473415987045	TRUE
**5448**	149479	149933	0.01125837113476016	SF370	155240	155571	71.2	FALSE	0.020826826742172695	FALSE
**5448**	289280	289620	0.011800497885785624	SF370	270256	270596	100.0	TRUE	0.012104571593766784	TRUE
**5448**	575949	576126	0.01034553624474901	SF370	597820	597997	100.0	TRUE	0.01525247166468627	TRUE
**5448**	441415	441558	0.010171565401530212	SF370	422383	422526	100.0	TRUE	0.013729359602945577	TRUE

Target Strain	Region Start	Region End	Target_Cov	Ref Strain	Ref Start	Ref End	Alignment Score Norm.	Homolog	Ref Cov	Expression Conserved
**5448**	1215296	1215428	0.011806289164002752	SF370	1241948	1242080	100.0	TRUE	0.020715668882553055	FALSE
**5448**	448351	448474	0.008190202605153605	SF370	429319	429442	100.0	TRUE	0.016255511663884777	TRUE
**5448**	1829715	1829836	0.017334955279314855	SF370	109	230	100.0	TRUE	0.12158330937543402	FALSE
**5448**	415278	415388	0.015354788020126466	SF370	396246	396356	100.0	TRUE	0.02114353605087216	FALSE

Supplementary Table 12. Nucleotide sequence conservation of the *amyA* locus between different serotypes of *S. pyogenes*. For this analysis, we selected the genomic region of *S. pyogenes* SF370 including the regions of homologous recombination as well as the sequence that is deleted by allelic replacement (coordinates used: start 1,078,523, end: 1,082,749) and performed a multiple sequence alignment. Sequence identity shown in % was analyzed using the integrated MUSLE alignment tool using standard parameters within the geneious software.

	M28 GAS6180	M4 GAS10750	M1 SF370	M1T1 5448
M28 GAS6180		99.882	99.882	99.882
M4 GAS10750	99.882		99.953	99.953
M1 SF370	99.882	99.953		100.000
M1T1 5448	99.882	99.953	100.000	

Supplementary Table 13. Nucleotide sequence conservation of the *sagB* locus between different serotypes of *S. pyogenes*. For this analysis, we selected the genomic region of *S. pyogenes* SF370 including the regions of homologous recombination as well as the sequence that is deleted by allelic replacement (coordinates used: start 598,115, end: 599,750) and performed a multiple sequence alignment. Sequence identity shown in % was analyzed using the integrated MUSLE alignment tool using standard parameters within the geneious software.

	M49 NZ131	M12 GAS9429	M28 GAS6180	M1 SF370	M1T1 5448	M4 GAS10750	M44 STAB901	M3 GAS315	M53 Alab49	M18 GAS8232	M5 Manfredo	M6 GAS10394
M49 NZ131		99.450	99.511	99.511	99.511	99.511	99.328	84.674	83.136	81.591	81.924	81.980
M12 GAS9429	99.450		99.572	99.572	99.572	99.572	99.389	84.792	83.249	81.813	82.147	82.202
M28 GAS6180	99.511	99.572		100.000	100.000	99.756	99.572	84.733	83.192	81.646	81.980	82.036
M1 SF370	99.511	99.572	100.000		100.000	99.756	99.572	84.733	83.192	81.646	81.980	82.036
M1T1 5448	99.511	99.572	100.000	100.000		99.756	99.572	84.733	83.192	81.646	81.980	82.036
M4 GAS10750	99.511	99.572	99.756	99.756	99.756		99.694	84.850	83.305	81.758	82.091	82.147
M44 STAB901	99.328	99.389	99.572	99.572	99.572	99.694		84.792	83.136	81.803	81.924	81.980
M3 GAS315	84.674	84.792	84.733	84.733	84.733	84.850	84.792		93.078	92.677	92.620	92.620
M53 Alab49	83.136	83.249	83.192	83.192	83.192	83.305	83.136	93.078		98.800	99.086	99.086
M18 GAS8232	81.591	81.813	81.646	81.646	81.646	81.758	81.803	92.677	98.800		99.268	99.155
M5 Manfredo	81.924	82.147	81.980	81.980	81.980	82.091	81.924	92.620	99.086	99.268		99.887
M6 GAS10394	81.980	82.202	82.036	82.036	82.036	82.147	81.980	92.620	99.086	99.155	99.887	

Supplementary Table 14. Nucleotide sequence conservation of the *Spy_1078* locus between different serotypes of *S. pyogenes*. For this analysis, we selected the genomic region of *S. pyogenes* SF370 including the regions of homologous recombination as well as the sequence that is deleted by allelic replacement (coordinates used: start 884,129, end: 886,197) and performed a multiple sequence alignment. Sequence identity shown in % was analyzed using the integrated MUSLE alignment tool, using standard parameters within the geneious software.

	M44_STAB901	M12_MGAS9429	M1 SF370	M1T1 5448
M44 STAB901		99.228	99.275	99.227
M12 MGAS9429	99.228		99.372	99.324
M1 SF370	99.275	99.372		99.952
M1T1 5448	99.227	99.324	99.952	

References

1. Hansen S, Vulić M, Min J, Yen TJ, Schumacher MA, Brennan RG, *et al*. Regulation of the *Escherichia coli* HipBA toxin-antitoxin system by proteolysis. PLoS ONE (2012) 7(6):e39185. doi: 10.1371/journal.pone.0039185.

2. Youngman P, Perkins JB, Losick R. Construction of a cloning site near one end of Tn917 into which foreign DNA may be inserted without affecting transposition in *Bacillus subtilis* or expression of the transposon-borne *erm* gene. Plasmid (1984) 12(1):1–9.

3. Bugrysheva JV, Scott JR. The ribonucleases J1 and J2 are essential for growth and have independent roles in mRNA decay in *Streptococcus pyogenes*. Mol Microbiol (2010) 75(3):731–43.

4. Bryan EM, Bae T, Kleerebezem M, Dunny GM. Improved vectors for nisin-controlled expression in Gram-positive bacteria. Plasmid (2000) 44(2):183–90.

5. Loh JMS, Proft T. Toxin–antitoxin-stabilized reporter plasmids for biophotonic imaging of Group A Streptococcus. Appl Microbiol Biotechnol (2013) 97(22):9737–45.

6. Bryksin AV, Matsumura I. Rational design of a plasmid origin that replicates efficiently in both Gram-positive and Gram-negative bacteria. PLoS ONE (2010) 5(10):e13244. doi: 10.1371/journal.pone.0013244.

7. Biswas I, Jha JK, Fromm N. Shuttle expression plasmids for genetic studies in *Streptococcus mutans*. Microbiol Read Engl (2008) 154(Pt 8):2275–82.

8. Radeck J, Kraft K, Bartels J, Cikovic T, Dürr F, Emenegger J, *et al*. The *Bacillus* BioBrick Box: generation and evaluation of essential genetic building blocks for standardized work with *Bacillus subtilis*. J Biol Eng (2013) 7(1):29. doi: 10.1186/1754-1611-7-29.

9. Liu X, Gallay C, Kjos M, Domenech A, Slager J, van Kessel SP, *et al*. High-throughput CRISPRi phenotyping identifies new essential genes in *Streptococcus pneumoniae*. Mol Syst Biol (2017) 13(5):931. doi: 10.15252/msb.20167449.

10. Wulff TF, Hahnke K, Lécrivain AL, Schmidt K, Ahmed-Begrich R, Finstermeier K, *et al*. Dynamics of diversified A-to-I editing in *Streptococcus pyogenes* is governed by changes in mRNA stability. bioRxiv [Preprint] (2023). Available at: http://biorxiv.org/lookup/doi/10.1101/2023.09.19.555891 (Accessed Sep 29, 2023)

11. McShan WM, McLaughlin RE, Nordstrand A, Ferretti JJ. Vectors containing streptococcal bacteriophage integrases for site-specific gene insertion. Methods Cell Sci (1998) 20(1):51–7.

12. Solovyev V, Salamov A. "Automatic Annotation of Microbial Genomes and Metagenomic Sequences 3" MATERIAL AND METHODS Learning Parameters and Prediction of Protein-Coding Genes. (2013).
